# Correlating functional near-infrared spectroscopy with underlying cortical regions of 0-, 1-, and 2-year-olds using theoretical light propagation analysis

**DOI:** 10.1117/1.NPh.8.2.025009

**Published:** 2021-05-31

**Authors:** Lin Cai, Eiji Okada, Yasuyo Minagawa, Hiroshi Kawaguchi

**Affiliations:** a Keio University, Department of Electronics and Electrical Engineering, Yokohama, Japan; b Keio University, Department of Psychology, Tokyo, Japan; c National Institute of Advanced Industrial Science and Technology, Human Informatics and Interaction Research Institute, Tsukuba, Japan

**Keywords:** functional near-infrared spectroscopy, light propagation, scalp-cortex correlation, early development

## Abstract

**Significance:** The establishment of a light propagation analysis-based scalp-cortex correlation (SCC) between the scalp location of the source–detector (SD) pair and brain regions is essential for measuring functional brain development in the first 2 years of life using functional near-infrared spectroscopy (fNIRS).

**Aim:** We aimed to reveal the optics-based SCC of 0-, 1-, and 2-year-olds (yo) and the suitable SD distance for this age period.

**Approach:** Light propagation analyses using age-appropriate head models were conducted on SD pairs at 10-10 fiducial points on the scalp to obtain optics-based SCC and its metrics: the number of corresponding brain regions ($N_{CBR}$), selectivity and sensitivity of the most likely corresponding brain region (MLCBR), and consistency of the MLCBR across developmental ages. Moreover, we assessed the suitable SD distances for 0-, 1-, and 2-yo by simultaneously considering the selectivity and sensitivity of the MLCBR.

**Results:** Age-related changes in the SCC metrics were observed. For instance, the $N_{CBR}$ of 0-yo was larger than that of 1- and 2-yo. Conversely, the selectivity of 0-yo was lower than that of 1- and 2-yo. The sensitivity of 1-yo was higher than that of 0-yo at 15- to 30-mm SD distances and higher than that of 2-yo at 10-mm SD distance. Notably, the MLCBR of the fiducial points around the longitudinal fissure was inconsistent across age groups. An SD distance between 15 and 25 mm was found to be appropriate for satisfying both sensitivity and selectivity requirements. In addition, this work provides reference tables of optics-based SCC for 0-, 1-, and 2-yo.

**Conclusions:** Optics-based SCC will be informative in designing and explaining child developmental studies using fNIRS. The suitable SD distances were between 15 and 25 mm for the first 2 years of life.

## 1 Introduction

The first two years of human life are characterized by the most dynamic growth in brain structures^1^^–^^5^ and remarkable cognitive and behavioral changes.^6^^,^^7^ Functional near-infrared spectroscopy (fNIRS; a list of abbreviations is provided in Table S1 in the Supplementary Material for the convenience of the reader) is an irreplaceable neuroimaging tool for studying early brain functional development, providing unprecedented opportunities for recording the hemodynamic response in awake, behaving infants because of its balanced temporal-spatial resolution and resilience to movement.^8^^–^^11^ Notably, although the Centers for Disease Control and Prevention defines 0- to 1-year-olds (yo) and 2- to 3-yo children as infants and toddlers, respectively, we have described 0- to 2-yo children as infants in this study for readability.

Despite the suitability of fNIRS in infant studies, a major limitation of this technique is the inability of fNIRS data to provide structural information of the head tissue. In fNIRS measurements, a source–detector (SD) pair positioned on the scalp surface measures local concentration changes in oxygenated and deoxygenated hemoglobin caused by neural activity.^12^^–^^15^ On the other hand, while neural activity tied to a specific human function originates in local brain regions. The absence of structural information in the fNIRS signal makes it impossible to correlate the signal response with the anatomical brain regions. Therefore, in fNIRS studies, the scalp location where the SD pair is attached to its underlying brain region where the fNIRS signal originates should be mapped. We call this mapping the scalp-cortex correlation (SCC).

Several methods for obtaining SCC have been proposed for studies on adults; however, only a few studies have provided SCC for the infant population. Similar to adults, infant SCC is mostly based on a simple geometrical technique, i.e., correlating the location of the SD pair on the scalp, typically, the midpoint of the SD pair, with cortical regions in a simple point-to-point geometrical manner. For instance, researchers often referred to the international 10-20 or 10-10 system^16^ when attaching SD pairs on the scalp and then inferred the anatomical locations^17^^–^^19^ according to the geometrical SCC of the adult head^20^^,^^21^ or infant head.^22^^–^^25^ Notably, by linearly reducing the size of the adult heads, the virtual registration method^26^ has also been employed to estimate SCC in infant studies.^27^^–^^29^

As described above, the point-to-point geometrical SCC provides a tolerable estimation of the underlying brain regions for the absorption change acquired by the SD pair. Nevertheless, the geometrical SCC is based on the assumption that the absorption change occurs at the cortical projection point below the midpoint between the SD pair, and light scattering in the head tissue is not considered. Mounting evidence from light propagation analysis in the adult head revealed light scattering in the head tissue could have a considerable influence on the partial pathlength (PPL) in the brain and the spatial sensitivity profile (SSP).^30^^–^^34^ Notably, a few studies have already demonstrated that light propagation in the infant heads is distinct from that in adults owing to structural differences.^35^^,^^36^ Furthermore, very recent studies on adults have started considering light propagation in turbid media when calculating the SCC.^37^^,^^38^ However, to date, no light propagation analysis-based SCC data are available for 0- to 2-yo infants. In addition to age, SD distance must have a significant influence on optics-based SCC during early development. Only a few studies have examined the effect of SD distance on fNIRS sensitivity in infant brain tissue.^35^^,^^39^ For example, Fukui et al.^35^ found that fNIRS sensitivity to gray matter (GM) and white matter (WM) of neonates was modulated by the SD distance. These threads of evidence revealed that it remains largely unknown how age and SD distance affect optics-based SCC in 0- to 2-yo infants and how to choose an appropriate SD distance to ensure both the sensitivity to cerebral hemodynamics and the selectivity of signals from a specific brain region of interest. To address these issues, the current study aimed to create a precise optics-based SCC between SD pairs on the scalp fiducial point and brain regions defined by a macro-anatomical atlas by considering the light scattering in 0-, 1-, and 2-yo infant heads. In addition, we quantitatively characterized the changes in SCC with age and SD distance and evaluated the suitable SD distances for each age. The optics-based SCC was obtained for each SD pair by solving the diffusion equation.

## 2 Materials and Methods

### 2.1 Infant Head Structure and AAL Atlas

The anatomical head structure of the infants and the corresponding brain atlas used in this study were obtained from publicly available data.^40^ In the present study, we used age-appropriate average structural images acquired with a 3T magnetic resonance imaging (MRI) scanner. A set of longitudinal images of 95 healthy infants [56 males and 39 females, gestational age at birth: 37.9±1.8 (mean ± standard deviation) weeks] were scanned three times when their postmenstrual age was 41.5±1.7, 94.2±3.4, and 146.2±4.9 weeks, respectively. Based on the difference in subtracting gestational age from postmenstrual age, the population used in this study could be divided into three age groups concentrating around 0, 1, and 2 years of age. All participants in our dataset had normal fetal ultrasound during pregnancy and were free of congenital anomalies, metabolic disease, and focal lesions after birth. T2-weighted images were obtained with a voxel size of $1.25\times 1.25\times 1.95\;{\mathrm{mm}}^{3}$ for 0-yo and T1-weighted images were obtained with a voxel size of $1\times 1\times 1\;{\mathrm{mm}}^{3}$ for 1- and 2-yo. The tissue probability maps of GM, WM, and cerebrospinal fluid (CSF) that exhibited similar geometry to the structural images were also used for head tissue segmentation (see Sec. 2.2.1).

To obtain the optics-based SCC between the fiducial points and brain regions, we chose the automated anatomical labeling (AAL) atlas^41^ to parcellate the infant’s brain. The AAL atlas is widely used in cognitive neuroscience. In addition, remarkably, it parcellates a human brain into multiple non-overlapping regions according to the identification of main sulci, which are already clearly visible from birth and preserved throughout normal brain development.^22^ We used the AAL atlas with 90 brain regions (Table S2 in the Supplementary Material) in the infant space, which maintains the consistency of the AAL map propagation from the adult Colin 27 brain to the infant images using indirect fusion approach and a feature-based groupwise registration algorithm (see Ref. 40). Infant atlases from 0-, 1-, and 2-yo were built using infant MRI segmentation and groupwise registration methods. The atlases are publicly available on the NITRC website.^42^

### 2.2 Light Propagation Analysis

#### 2.2.1 Construction of age-appropriate structural head models

**Fig. 1:**
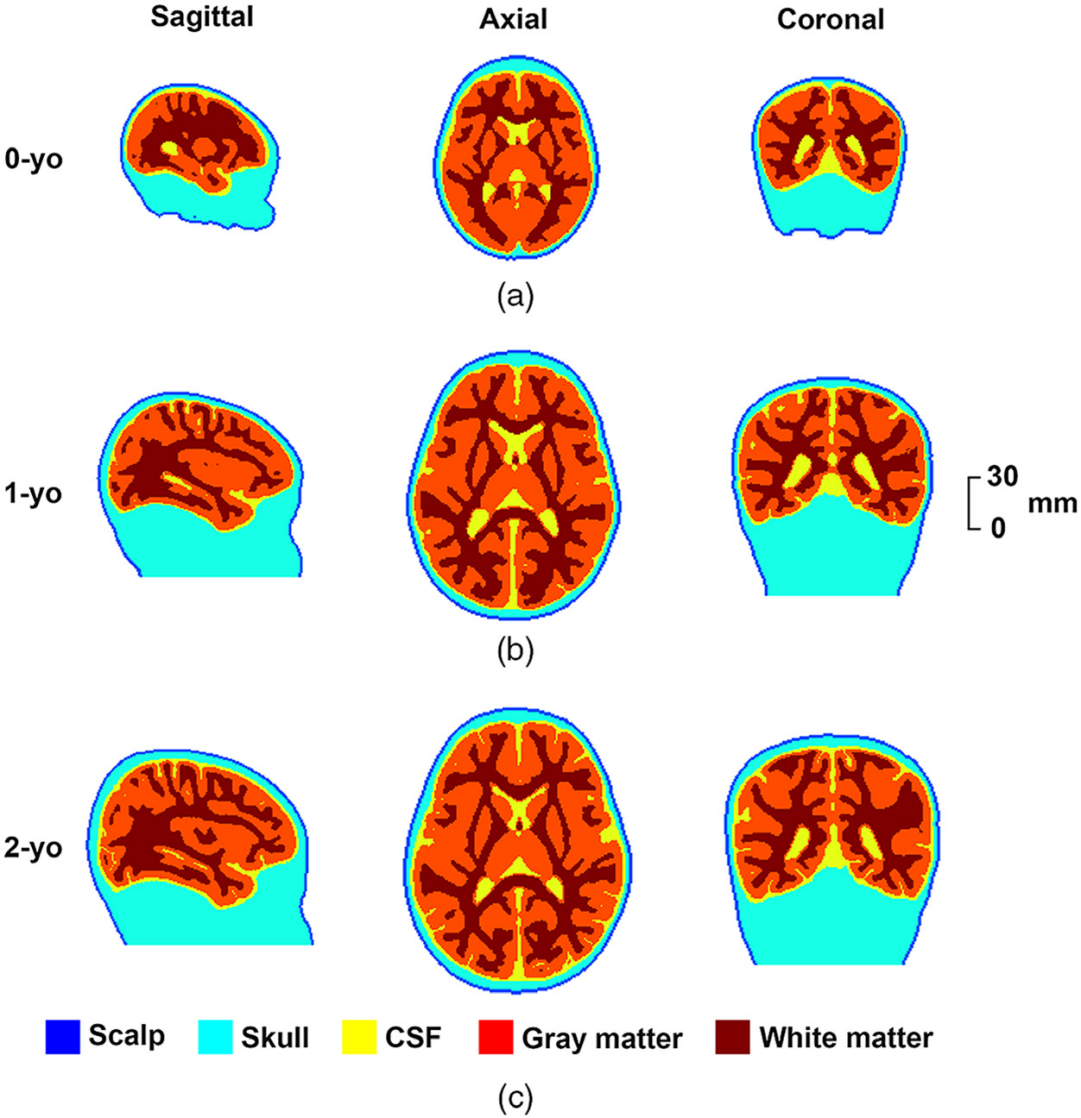
Age-appropriate five-layered head models for (a) 0-yo; (b) 1-yo; and (c) 2-yo infants, which comprise the scalp, skull, CSF, GM, and WM. The first, second, and third columns show the sagittal, axial, and coronal views of the head models, respectively.

We can use an age-appropriate template of the head to substitute the subject-specific head anatomy to localize the macroanatomical structure when individual infant MRIs are not available.^24^^,^^25^ Hence, we constructed three age-appropriate head models by segmenting the tissues of average MRI images from 0-, 1-, and 2-yo infants for further light propagation analysis. Specifically, a semiautomatic approach was used to segment the MRI images into air and five types of head tissues with different optical properties. First, the head masks, i.e., the air/scalp boundaries, were extracted from the average T2-weighted images for 0-yo, and T1-weighted images for 1- and 2-yo using a simple thresholding method. Second, the tissue probability maps of the CSF, GM, and WM were converted to binary intracranial regions in each image. It was difficult to identify the scalp/skull and skull/CSF boundaries in the average MRI images because of the thin structures of the scalp and skull and blurring caused by the averaging of slightly misaligned multi-subject images. Thus, we applied morphological operations to extract the scalp/skull and skull/CSF boundaries based on the representative thickness of the scalp (3.5, 4, and 4 mm for 0-, 1-, and 2-yo, respectively) and skull (2.2, 3, and 3.8 mm for 0-, 1-, and 2-yo, respectively), as described in the literature.^43^^,^^44^ Finally, five-layered head models of 0-, 1-, and 2-yo infants were created by integrating the above intracranial regions and boundaries of superficial tissues. Contradictions in the integration process were manually and/or automatically corrected. The age-appropriate five-layered models for the three age groups are shown in Fig. 1.

#### 2.2.2 Light propagation calculation and optical property

The volumetric tetrahedral mesh for each age-appropriate head model was generated using the iso2mesh toolbox^45^ for light propagation analysis by employing the diffusion equation along with the finite element method. Similar to a previous study,^46^ we confirmed that the quality of the created volumetric tetrahedral mesh was sufficient for conducting the light propagation analysis. Specifically, we calculated the number of nodes, elements, and faces in each mesh model and further computed the Joe–Lie quality index,^47^ $q_{vol}$, for every tetrahedron for all three ages using the following equation:

$$q_{vol}=\frac{12\times {\left(3\times vol\right)}^{\frac{2}{3}}}{\sum_{0\leq i\leq j\leq 3}l_{i,j}^{2}},$$

where *vol* is the tetrahedral volume, and $l_{i,j}$ are the lengths of the edges of the tetrahedron. This metric is equal to 1 for equilateral tetrahedra and tends to zero for degenerated tetrahedra. The higher the $q_{vol}$ value, the higher the quality of the mesh. In Table 1, we report the total number (N) of nodes, elements, and faces. The mean $q_{vol}$ (across all tetrahedrons) with its standard deviation is also shown. We also found the majority of volumetric tetrahedral meshes for each age group had a high Joe–Liu quality value (76.9%, 80.5%, and 79.2% of all meshes have Joe–Liu quality values higher than 0.7 for 0- to 2-yo infants). Light propagation in the head models was calculated using the Nirfast software,^48^ a finite element-based package that uses the diffusion approximation for modeling near-infrared light transport in tissue.^49^^,^^50^ The optical properties of each tissue type in the infant head models for a wavelength of 800 nm were specified as in previous studies,^35^^,^^51^ namely, the absorption coefficient $\mu_{a}$, scattering coefficient $\mu_{s}$, anisotropy factor g, and refractive index n (Table 2). Notably, the reduced scattering coefficient ${\mu_{s}}^{\prime }$ should be used to analyze light propagation using the diffusion equation, where ${\mu_{s}}^{\prime }=\mu_{s}(1-g)$.

**Table 1:** Properties of the volumetric mesh (number of nodes, elements, faces, and the Joe–Liu quality index) for every age.

	0-yo	1-yo	2-yo
N nodes	101056	212418	243541
N elements	594241	1260896	1451619
N faces	293208	519898	555656
Mean $q_{vol}\pm \mathrm{std}$	0.784±0.131	0.799±0.123	0.793±0.127

**Table 2:** Optical properties of tissue types for light propagation analysis.

	$\mu_{a}$ (${\mathrm{mm}}^{-1}$)	$\mu_{s}$ (${\mathrm{mm}}^{-1}$)	g	n
Scalp	0.018	19	0.9	1.4
Skull	0.016	16	0.9	1.4
CSF	0.0041	0.32	0.9	1.4
GM	0.048	5.0	0.9	1.4
WM	0.037	10	0.9	1.4

#### 2.2.3 Fiducial points and arrangement of SD pairs

**Fig. 2:**
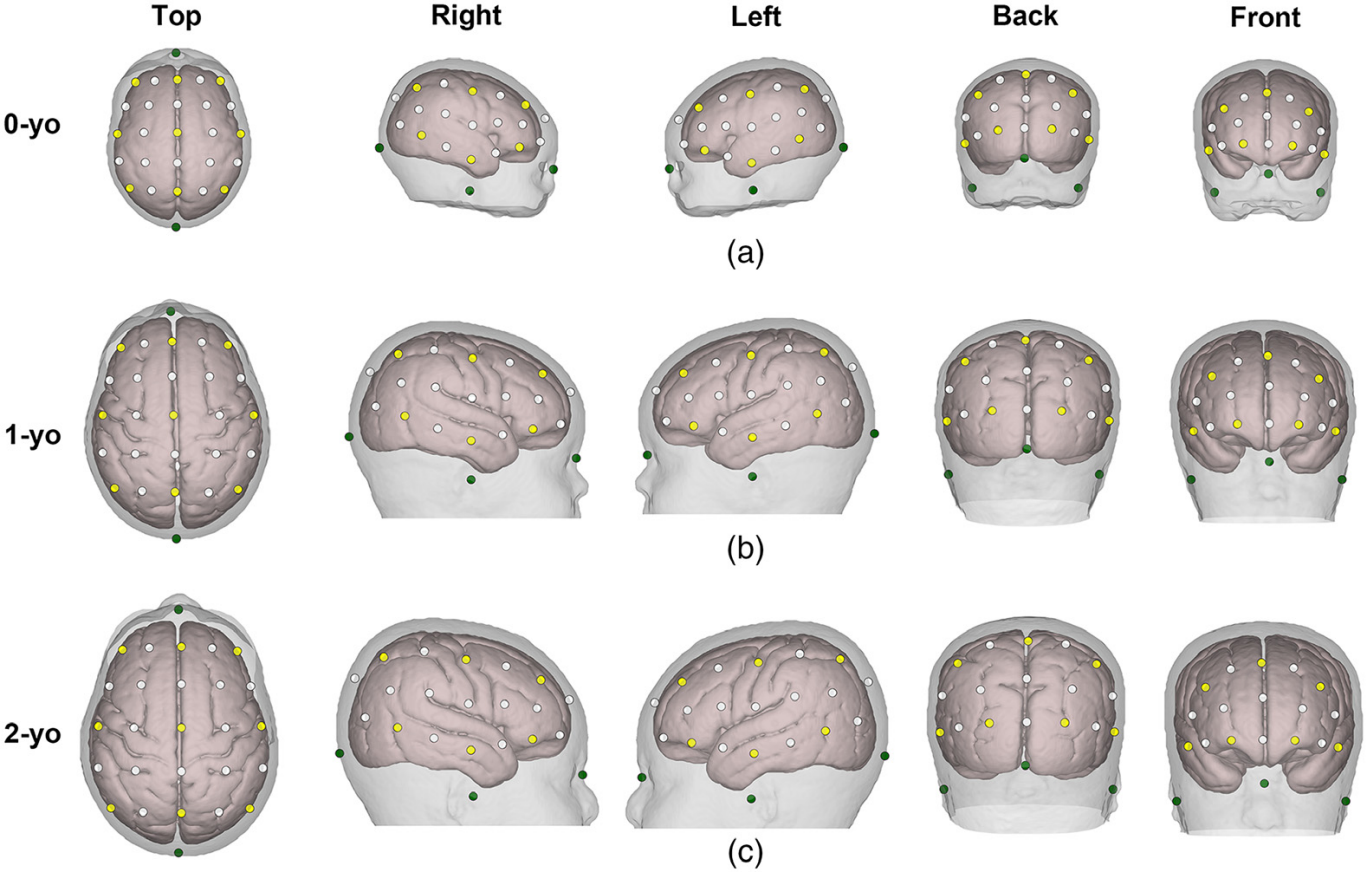
Superimposed anatomical landmarks (in dark green), 10-20 fiducial points (in yellow), and 10-10 fiducial points (in white) on the head models of (a) 0-yo; (b) 1-yo; and (c) 2-yo. For each row panel, five views of the head models are displayed.

The international 10-10 system positions were virtually set on the age-appropriate head models of 0-, 1-, and 2-yo using custom analysis scripts written in MATLAB (MathWorks, Natick, Massachusetts). First, we manually identified the four anatomical landmarks, including the inion, nasion, and left and right periauricular points on the scalp surface of the infant head models. Then, 61 fiducial points of the 10-10 system^16^^,^^52^ were automatically assigned to the scalp of the head model of every age, as depicted in Fig. 2.

**Fig. 3:**
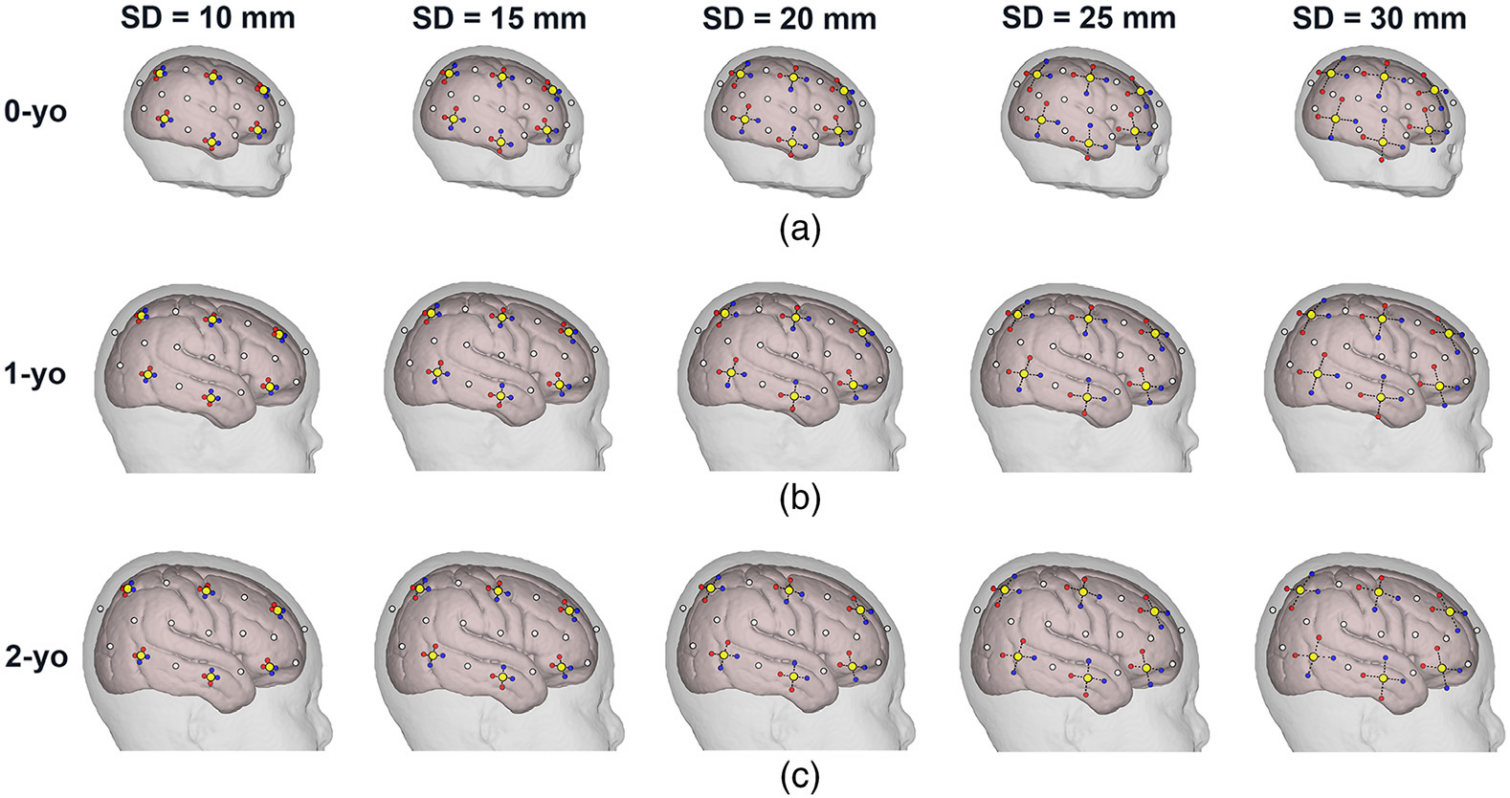
SD pair arrangements on head models for five different SD distances for (a) 0-yo; (b) 1-yo; and (c) 2-yo. The sources and detectors are indicated in red and blue dots, respectively. Two orientational SD pairs at the 10-20 fiducial points (yellow dots) are displayed only on the right hemisphere. Notably, SD pairs were also attached to the 10-10 fiducial points (white dots); however, the sources and detectors at these locations are not shown to avoid complications. The circumferential and vertical SD pairs at each fiducial point are indicated by two dashed black connections, respectively.

Because the SD distance significantly affects the sampling regions, the distances between sources and detectors were set as 10, 15, 20, 25, and 30 mm, where the midpoints of the SD pairs were set at the 10-10 fiducial points. Two SD pairs were placed according to the circumferential and vertical orientations at each fiducial point to examine whether the probe orientation influences the SCC for 0-, 1-, and 2-yo. The SD pair arrangements on the head models for five different SD distances for each age are shown in Fig. 3.

#### 2.2.4 Calculation of photon measurement density function and normalized PPL

Similar to our previous adult study,^38^ we adopted the optics-based method to analyze SCC. Based on the results of the light propagation analysis, the photon measurement density function (PMDF), which has the same spatial distribution as SSP,^53^ was calculated at each node of the mesh model.^54^ As the sum of the SSPs is equivalent to the PPL in a particular brain region, the PMDF sum is linearly related to the PPL for that region. Given that each node in the mesh model was labeled as a specific AAL brain region, we could define the normalized PPL ($L_{norm,M}$) in a given brain region M using the following equation to quantify the SCC:

$$L_{norm,M}=\frac{l_{M}}{\Sigma_{j=1}^{N}l_{j}},$$

where $l_{M}$ and $l_{j}$ are the sum of the PMDFs of all nodes within the brain regions M and j, respectively. N is the total number of brain regions within the whole brain tissue. According to the equation, $L_{norm,M}$ ranges from 0 to 1. Because the PMDF is a probability density function,^55^ we could use $L_{norm,M}$ as an index to represent the probability that the fNIRS signal is affected by the brain activation of region M. We calculated $L_{norm,M}$ of two orientational SD pairs (circumferential and vertical orientations) placed at every fiducial point for the 0-, 1-, and 2-yo head models.

### 2.3 Characterization of SCC

#### 2.3.1 Evaluation metrics

For fNIRS users who are involved in developmental neuroscience, the following four questions are foremost: (1) how many brain regions are associated with an SD pair; (2) which brain region is the most likely corresponding brain region (MLCBR) for a given SD pair and its probability; (3) whether the sensitivity is sufficient to measure brain activity in the MLCBR, and (4) whether the MLCBR for the same scalp location is consistent across early development. Therefore, we defined four metrics, namely, the number of corresponding brain regions ($N_{CBR}$), the selectivity of the MLCBR, the sensitivity of the MLCBR, and the consistency of the MLCBR across physical development, to further characterize the SCC and to systematically investigate the effects of age and SD distance on the SCC.

The detailed definitions of the four metrics are described below. Due to the strong scattering of near-infrared light passing through head tissues, a fiducial point usually projects to more than one brain region. Hence, we simply counted the number of brain regions that were correlated to a given fiducial point as *the* $N_{CBR}$. The current study used $L_{norm,M}=0.05$, as a threshold to calculate $N_{CBR}$. Moreover, we chose the brain region with the largest $L_{norm,M}$, as the MLCBR for a given fiducial point. $L_{norm,MLCBR}$ were defined as the metrics of selectivity at the fiducial point. For the sensitivity metric, we calculated the absolute PPL of MLCBR. The absolute PPL in the MLCBR was obtained using the following equation:

$$L_{abs,MLCBR}=\frac{\ln(I_{base}/I_{pert})}{0.001\mu_{a,MLCBR}},$$

where $I_{base}$ and $I_{pert}$ are the detected intensities when the absorption coefficient of the MLCBR ($\Delta \mu_{a,MLCBR}$) is the baseline and perturbed states (0.1% increase), respectively. Finally, to examine whether the MLCBR at the same fiducial point was consistent from 0- to 2-yo, all fiducial points were classified into five categories according to consistency: (1) completely consistent, i.e., the MLCBR was the same for the brain region among the three ages; (2) consistent between 0- and 1-yo; (3) consistent between 0- and 2-yo; (4) consistent between 1- and 2-yo; and (5) inconsistent between any age groups.

#### 2.3.2 Statistical analysis

Statistical analyses were performed using R. Almost all analyses were performed using R version 3.6.3 in R Studio Version 1.2.5033 (RStudio Team, 2019). The following packages were used for data manipulation, visualization, and statistical tests: dplyr v1.0.2, tidyverse v1.3.0, rstatix v0.6.0, magrittr v1.5, nparLD v2.1, and ggpubr v0.4.0. Only $N_{CBR}$ was assessed with R 3.5.1, using the nparLD package. The $N_{CBR}$, selectivity, and sensitivity values at each 10-10 fiducial point (61 in total) were treated as dependent variables. Provided that the dependent variable $N_{CBR}$ deviated from normality and equal variability, we applied a rank-based non-parametric mixed model statistical method, nparLD^56^ with an F1-LD-F1 design, to investigate the effects of age and SD distance on $N_{CBR}$. We reported the Wald-type statistic (WTS) to assess the statistical significance of age, SD distance, and their interaction. If the interaction or main effects were significant, comparisons between two conditions were conducted using the nonparametric Mann-Whitney U test (independent samples) or Wilcoxon signed-rank test (dependent samples), followed by the Bonferroni method for multiple comparison adjustment. For the dependent variables of sensitivity or selectivity, a mixed-design ANOVA was conducted to examine the effects of age and SD distance. Specifically, the sensitivity and selectivity were subjected to two two-way mixed ANOVAs with the SD distance (10, 15, 20, 25, and 30 mm) as a within-subjects factor and age (0-, 1-, and 2-yo) as a between-subjects factor. If the interaction or main effects were significant, comparisons between two conditions were conducted using a two-sample t-test (independent samples) or paired t-test (dependent samples), followed by the Bonferroni method for multiple comparison adjustment. In all ANOVA analyses, Greenhouse–Geisser corrections were applied on violation of the sphericity assumption. The generalized eta squared^57^^,^^58^ served as estimates of the effect sizes.

## 3 Results

### 3.1 Optics-Based SCC for a Representative Fiducial Point

**Fig. 4:**
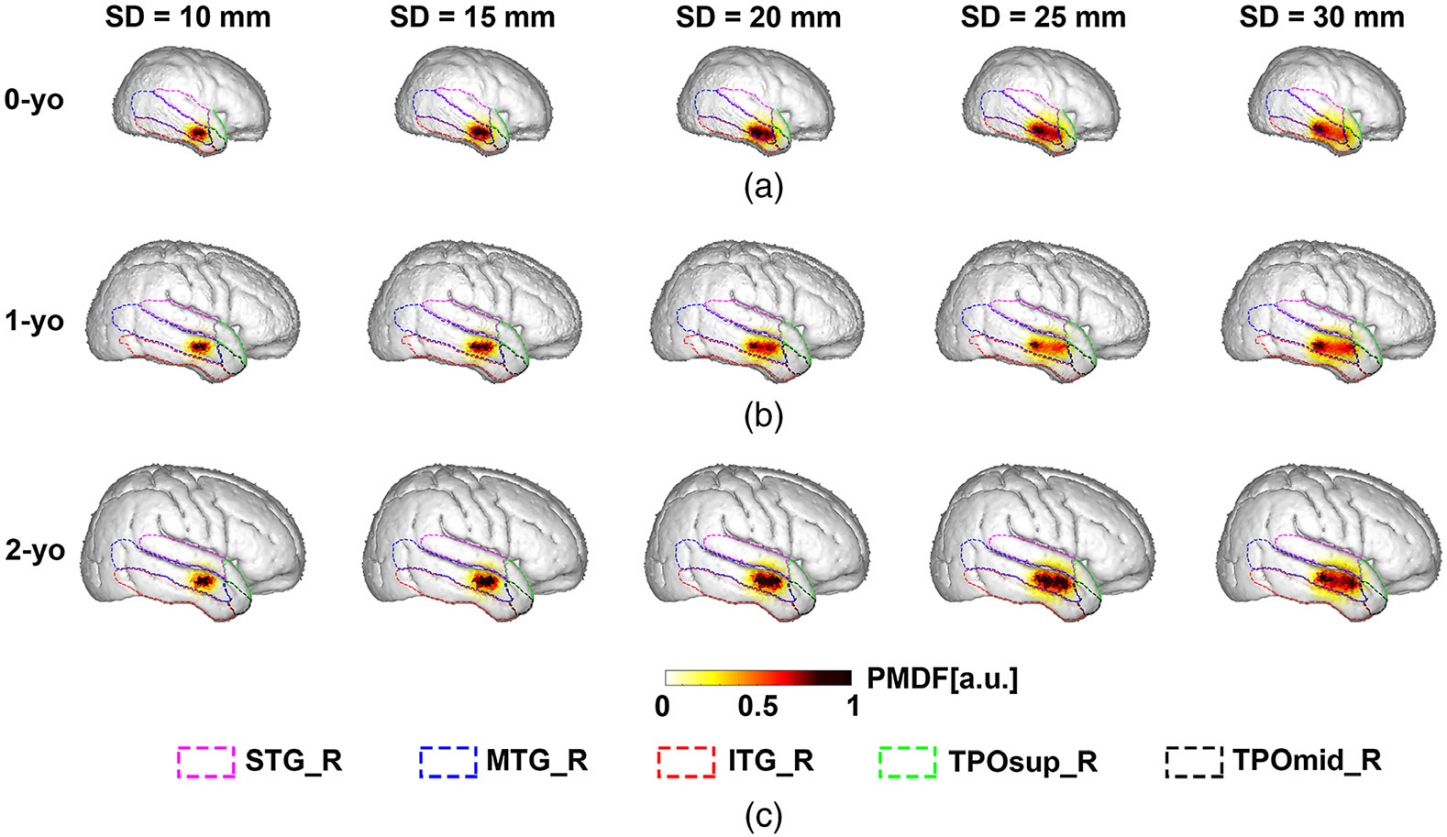
PMDF for a given fiducial point T4 at five SD distances for 0-yo, 1-yo, and 2-yo when the SD pair was attached in a circumferential orientation. Dashed lines in different colors indicate the AAL brain region boundaries. The PMDF superimposed on age-appropriate brain structures of (a) 0-yo; (b) 1-yo; and (c) 2-yo.

To illustrate the changes in SSP due to SD distance and age, the PMDF of the circumferential SD pair at the fiducial point T4 is shown in Fig. 4. The spatial distribution of the PMDF broadened as the SD distance increased, regardless of age. The values of $L_{norm,M}$ higher than 0.01, at the fiducial point T4 are shown in Table 3. According to the definition of the $N_{CBR}$ (i.e., the number of brain regions whose $L_{norm,M}$ was >0.05), $N_{CBR}$ was 2 at the fiducial point T4, which depicts the right middle and inferior temporal gyrus (MTG-R and ITG-R, respectively). These two brain regions had dominant $L_{norm,M}$ at the fiducial point T4 for any age and SD distance (indicated in boldface in Table 3), where the MLCBR was MTG_R. In addition to MTG_R and ITG_R, other brain regions, the superior temporal gyrus (STG-R) and right temporal pole of the superior and middle temporal gyrus (TPOsup-R and TPOmid-R, respectively), were also associated with T4, and their $L_{norm,M}$ increased as the SD distance increased. Considering the STG_R of 0-yo as an example, T4 had a $L_{norm,STG\_R}$ of 0.009 for a 10-mm SD distance, whereas the $L_{norm,STG\_R}$ reached 0.022 when the SD distance was 30 mm (see Table 3). For the same SD distance, the $L_{norm,MTG\_R}$ of 1- or 2-yo was greater than that of 0-yo. For instance, the $L_{norm,MTG\_R}$ values for a 20-mm SD distance were 0.593, 0.786, and 0.861 for 0-, 1-, and 2-yo, respectively. The PMDF of the vertical SD pair set at fiducial point T4 is shown in Fig. S1 in the Supplementary Material. Likewise, we found that the vertical SD pair at T4 was mainly correlated with the two brain regions, that is, MTG_R and ITG_R. The maximum $L_{norm,STG\_R}$ of the vertical SD pair was only 0.060, 0.070, and 0.094 for 0-, 1-, and 2-yo, respectively (SD distance=30  mm). As shown in Table 3, the fiducial point T4 had the same MLCBR, that is, MTG_R, for any age and SD distance; therefore, the MLCBR was completely consistent among the three ages. The sensitivity of MLCBR ($L_{abs,MLCBR}$) is also shown in Table 3. We also observed an increase in the $L_{abs,MLCBR}$ at each age with increasing SD distance. The $L_{abs,MLCBR}$ of 0-yo was smaller than that of 1- and 2-yo, while $L_{abs,MLCBR}$ of 1-yo was almost similar or slightly larger than that of 2-yo.

**Table 3:** Normalized PPL of corresponding brain regions ($L_{norm,M}$) and the sensitivity of MLCBR ($L_{abs,MLCBR}$) for the circumferential SD pair set at the fiducial point T4 at five SD distances for 0-yo, 1-yo, and 2-yo.

Age	Brain region	SD distance				
		10 mm	15 mm	20 mm	25 mm	30 mm
0-yo	MTG_R	**0.636**	**0.651**	**0.593**	**0.611**	**0.562**
	ITG_R	**0.346**	**0.318**	**0.366**	**0.332**	**0.366**
	STG_R	0.009	0.013	0.014	0.021	0.022
	TPOsup_R	0.004	0.007	0.009	0.013	0.017
	TPOmid_R	0.003	0.006	0.010	0.013	0.020
	$L_{abs,MLCBR}$ (mm)	1.9	4.6	5.9	7.9	9.1
1-yo	MTG_R	**0.836**	**0.818**	**0.786**	**0.786**	**0.746**
	ITG_R	**0.153**	**0.168**	**0.196**	**0.188**	**0.224**
	STG_R	0.009	0.011	0.014	0.020	0.022
	TPOsup_R	0.001	0.001	0.001	0.002	0.003
	TPOmid_R	0.000	0.000	0.001	0.001	0.002
	$L_{abs,MLCBR}$ (mm)	4.6	6.2	8.0	10.4	11.3
2-yo	MTG_R	**0.890**	**0.869**	**0.861**	**0.824**	**0.819**
	ITG_R	**0.078**	**0.097**	**0.094**	**0.124**	**0.117**
	STG_R	0.026	0.027	0.034	0.037	0.046
	TPOsup_R	0.002	0.003	0.004	0.007	0.009
	TPOmid_R	0.001	0.001	0.001	0.003	0.003
	$L_{abs,MLCBR}$ (mm)	3.9	5.8	7.6	9.8	11.3

Note: The $L_{norm,M}$ value higher than 0.05, is shown in bold. The MLCBR at T4 was MTG_R for all ages and SD distances.

The brain regions correlated with the two orientational SD pairs at all 10-10 fiducial points and their normalized PPL from 0-, 1-, and 2-yo infants are provided in Tables S3-7 in the Supplementary Material for SD distances of 10, 15, 20, 25, and 30 mm, respectively.

### 3.2 Evaluation of SCC at All Fiducial Points

**Fig. 5:**
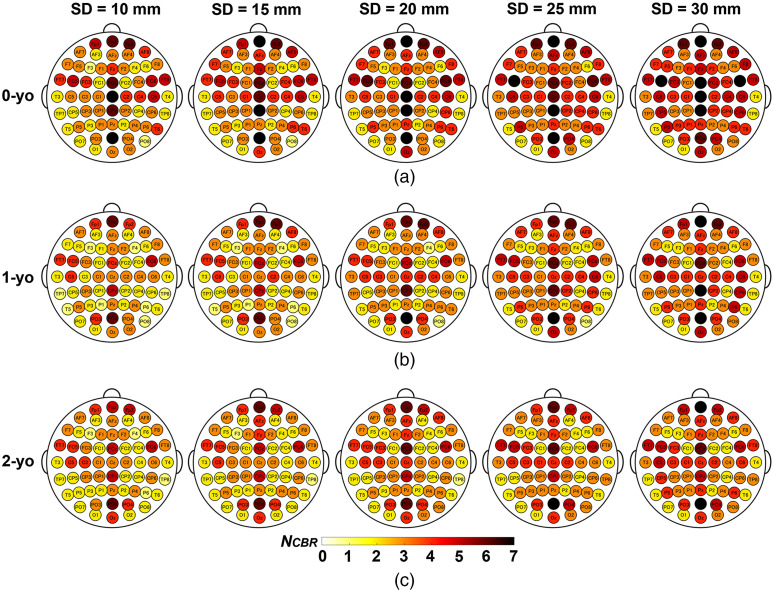
$N_{CBR}$ for all 10-10 fiducial points at five different SD distances for (a) 0-yo; (b) 1-yo; and (c) 2-yo. Darker red regions indicate larger $N_{CBR}$.

**Fig. 6:**
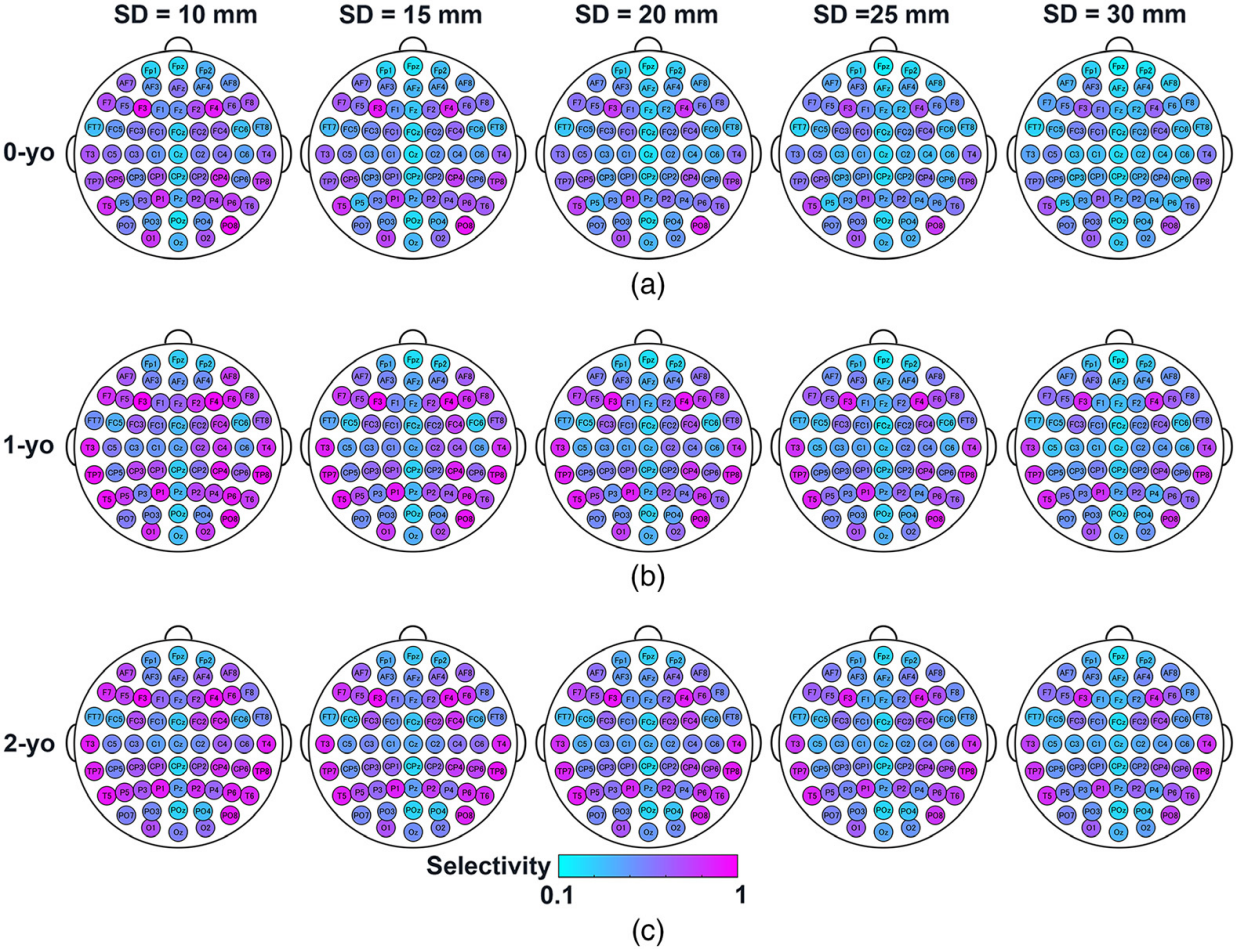
Selectivity of MLCBR for all 10-10 fiducial points at five different SD distances for (a) 0-yo; (b) 1-yo; and (c) 2-yo. Areas in magenta and blue indicate higher and lower selectivity, respectively.

**Fig. 7:**
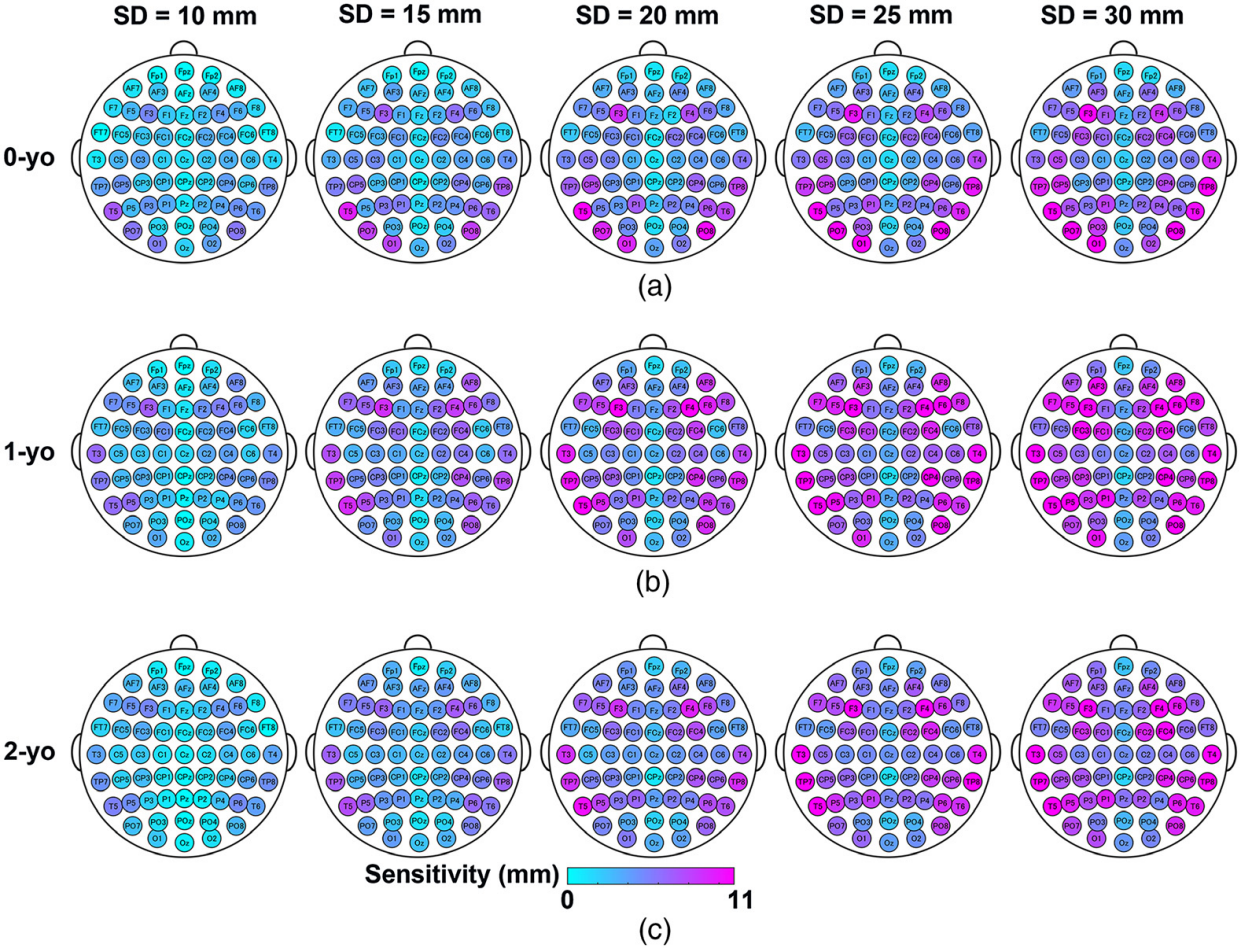
Sensitivity of MLCBR for all 10-10 fiducial points at five different SD distances for (a) 0-yo; (b) 1-yo; and (c) 2-yo. Area in magenta and blue indicate higher and lower sensitivity, respectively.

**Fig. 8:**
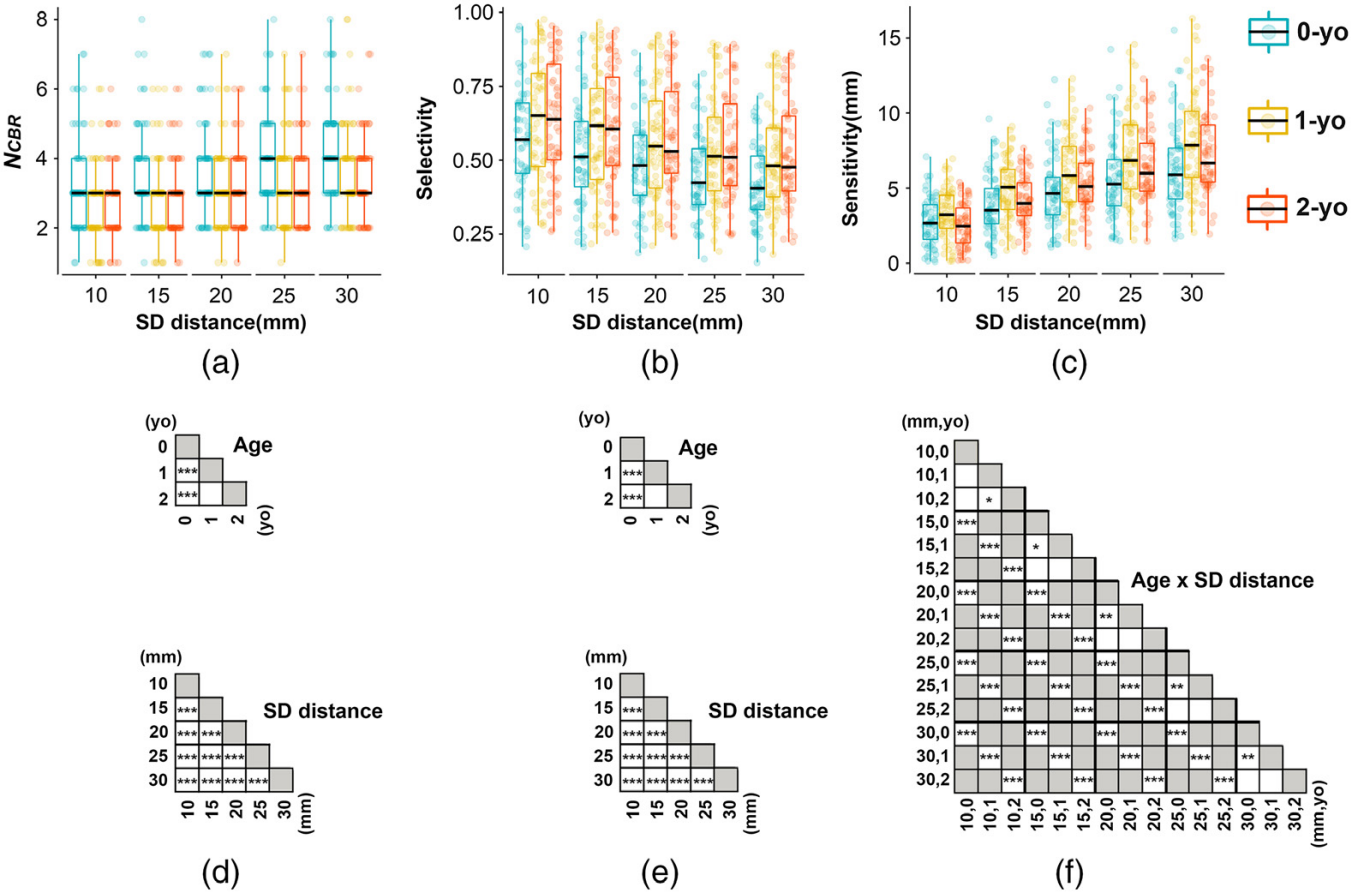
Box plots summarizing the SCC metrics, (a) $N_{CBR}$; (b) selectivity; and (c) sensitivity, at all scalp fiducial points of the 10-10 system for each age and SD distance. The individual colored dots indicate the SCC metrics of each fiducial point. Boxes indicate the interquartile range. The black horizontal line within the boxes indicates the median. Whiskers extend 1.5 times above and below the interquartile range limits. Statistical significance of post-hoc test for (d) $N_{CBR}$; (e) selectivity; and (f) sensitivity are indicated with matrices. * p<0.05, ** p<0.01, *** p<0.001, white and gray blanks are not significant and not applicable, respectively.

The SCC metrics $N_{CBR}$, selectivity, and sensitivity at all 10-10 fiducial points are shown in Figs. 5–7, respectively. The summary statistics of the SCC metrics across all fiducial points are shown in Fig. 8.

We calculated the $N_{CBR}$ value for all 10-10 fiducial points over five SD distances at three ages. As shown in Fig. 5 for circumferential SD pairs, fiducial points neighboring the longitudinal fissure, for example, Fpz, FCz, Cz, CPz, and POz, were correlated with more brain regions for every SD distance and age. Additionally, the larger the SD distance, the larger the $N_{CBR}$ of the fiducial points for every age group. The $N_{CBR}$ of 0-yo was larger than that of 1- and 2-yo, while the $N_{CBR}$ of 1- and 2-yo was similar for each SD distance. According to a nonparametric statistical analysis, we found significant main effects of SD distance [WTS(4)=195.88, p<0.001] and age [WTS(2)=10.37, p<0.01], but no interaction [WTS(8)=9.01, p=0.34]. For the age factor [Figs. 8(a) and 8(d) upper panel], multiple comparisons with the Bonferroni adjustment showed that the $N_{CBR}$ of 0-yo was larger than that of 1- and 2-yo (p<0.001, corrected); however, no significant differences were found between 1- and 2-yo (p=0.92, corrected). For the SD distance factor [Figs. 8(a) and 8(d) lower panel], multiple comparisons between $N_{CBR}$ of any two SD distances produced significantly different values, and the $N_{CBR}$ values of larger SD distances were greater than those of smaller SD distances (p<0.001, corrected). Similar results for the $N_{CBR}$ of the vertical SD pairs are shown in Fig. S2 in the Supplementary Material.

The selectivity of the MLCBR shown in Fig. 6 shows the ratio of the PPL in the MLCBR to its sum in the 90 AAL brain region. In a sense, the selectivity represents the probability that the fNIRS signal originates from the MLCBR. For most fiducial points, higher selectivity was observed for shorter SD distances, regardless of age. Moreover, the selectivity of 1- and 2-yo was larger than that of 0-yo. These observable findings were supported by statistically significant main effects of age [F(2,180)=4.89, p<0.05, ${\eta_{G}}^{2}=0.049$] and SD distance [F(1.41,253.25)=266.11, p<0.001, ${\eta_{G}}^{2}=0.067$] on selectivity; however, no statistically significant two-way interactions were found between age and SD distance on the selectivity, F(2.81,253.25)=0.93, p=0.42, ${\eta_{G}}^{2}=0.001$ [Fig. 8(b)]. The results of the post-hoc multiple comparisons are shown in Fig. 8(e). For the age factor [Fig. 8(e), upper panel], multiple pairwise independent sample t-tests showed that the selectivity of 0-yo was lower than that of 1- and 2-yo for all fiducial points on average (p<0.001, Bonferroni-corrected). In contrast, multiple pairwise paired t-tests for the SD distance [Fig. 8(e), lower panel] showed that comparisons from any two SD distances were significantly different (p<0.001, corrected) and that the selectivity decreased with increasing SD distance. Similar results for the selectivity of vertical SD pairs are shown in Fig. S3 in the Supplementary Material.

With regard to the sensitivity of the MLCBR over all 10-10 fiducial points, we found an obvious increase in $L_{abs,MLCBR}$ with increasing SD distance for each age (Fig. 7). However, no differences in age were observed during the visual inspection. The 3 (age) × 5 (SD distance) mixed ANOVA revealed significant main effects of age [F(2,180)=4.87, p<0.01, ${\eta_{G}}^{2}=0.047$] and SD distance [F(1.37,247.25)=837.02, p<0.001, ${\eta_{G}}^{2}=0.306$]. However, these main effects were further qualified by the presence of a significant interaction between age and SD distance, F(2.75,247.25)=8.67, p<0.001, ${\eta_{G}}^{2}=0.009$ [Fig. 8(c)]. The simple main effect of age was significant for all SD distances, that is, 10, 15, 20, 25, and 30 mm (all p<0.05). The results of the post-hoc multiple comparisons are shown in Fig. 8(f). At a 10-mm SD distance, the mean sensitivity of 1-yo was significantly higher than that of 2-yo (p<0.05, Bonferroni-corrected). The mean sensitivity of 1-yo was significantly higher than that of 0-yo at SD distances of 15 mm (p<0.05, corrected), 20 mm (p<0.01, corrected), 25 mm (p<0.01, corrected), and 30 mm (p<0.01, corrected). The simple main effect of the SD distance was also significant for any of the 0-, 1-, and 2-yo infants (all p<0.001). At all years of age, the sensitivity of larger SD distances was significantly greater than that of smaller SD distances [Figs. 8(c) and 8(f), p<0.001, corrected]. Similar results for the sensitivity of the vertical SD pairs are presented in Fig. S4 in the Supplementary Material.

**Fig. 9:**
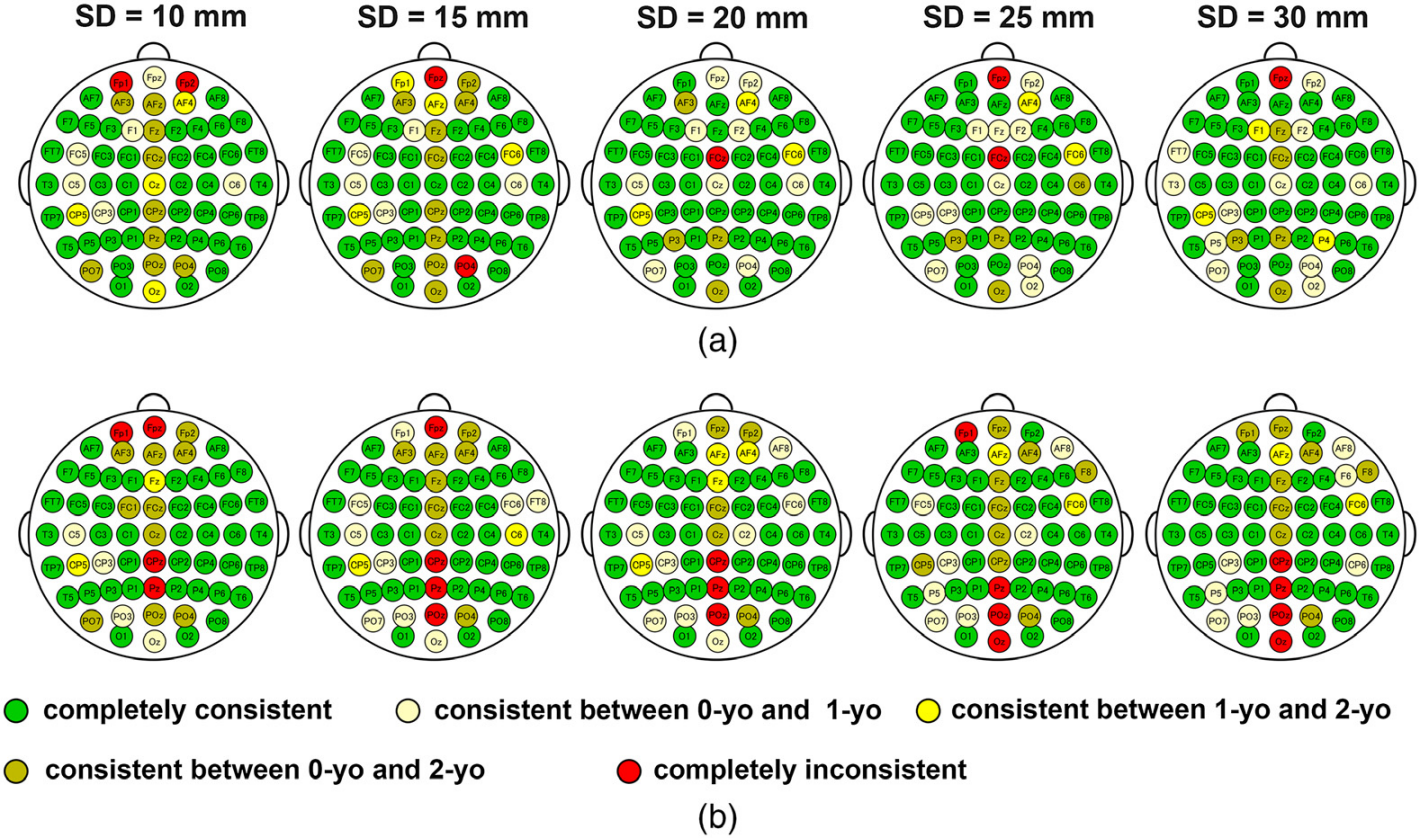
Consistency of MLCBR at five SD distances for (a) circumferential and (b) vertical SD pairs. The consistency of the MLCBR among 0-yo, 1-yo, and 2-yo for every fiducial point is indicated by circles with different colors.

We found that almost half of 10-10 fiducial points correlated with the completely consistent MLCBR across the three ages for each SD distance (Fig. 9). The average number of fiducial points correlated with the completely consistent MLCBR for all SD distances was 41.6±1.5 and 39.6±0.9 for circumferential and vertical SD pairs, respectively, as shown in green circles in Fig. 9. However, fiducial points correlated with inconsistent MLCBR across the three ages (yellow and red circles) were found around the longitudinal fissure for both SD pair orientations. To help fNIRS researchers examine longitudinal functional development from 0- to 2-yo, we have also provided fiducial points whose MLCBR was completely consistent at certain SD distances for the circumferential and vertical SD pairs across 0-, 1-, and 2-yo infants (Table 4).

**Table 4:** Summary of fiducial points whose MLCBR was completely consistent at some SD distances for the circumferential and vertical SD pairs across 0-yo, 1-yo, and 2-yo infants.

Fiducial point	Circumferential SD pairs		Vertical SD pairs	
	MLCBR	SD distance (mm)	MLCBR	SD distance (mm)
Cz	SMA-R	15	—	—
AFz	SFGmed-R	20, 25, 30	—	—
Fz	SFGmed-R	20	—	—
CPz	PoCG-L	20, 25, 30	—	—
POz	SPG-L	20, 25, 30	—	—
T3	MTG-L	10, 15, 20, 25	MTG-L	10, 15, 20, 25, 30
C5	STG-L	25, 30	PoCG-L	25, 30
C3	PoCG-L	10, 15, 20, 25, 30	PoCG-L	10, 15, 20, 25, 30
C1	PreCG-L	10, 15, 20, 25, 30	PreCG-L	10, 15, 20, 25, 30
C2	PreCG-R	10, 15, 20, 25, 30	PreCG-R	10, 15, 30
C4	PoCG-R	10, 15, 20, 25, 30	PoCG-R	10, 15, 20, 25, 30
C6	—	—	PoCG-R	10, 20, 25, 30
T4	MTG-R	10, 15, 20, 25, 30	MTG-R	10, 15, 20, 25, 30
FT7	TPOsup-L	10, 15, 20, 25	TPOsup-L	10, 15, 20, 25, 30
F7	ORBinf-L	10, 15, 20, 25, 30	ORBinf-L	10, 15, 20, 25, 30
AF7	ORBmid-L	10, 15, 20, 25, 30	ORBmid-L	10, 15, 20, 25, 30
Fp1	ORBmid-L	20, 25, 30	—	—
Fp2	—	—	SFGdor-R	25, 30
AF8	ORBmid-R	10, 15, 20, 25, 30	ORBmid-R	10, 15
F8	ORBinf-R	10, 15, 20, 25, 30	ORBinf-R	10, 15, 20
FT8	MTG-R	10, 15, 20, 25, 30	MTG-R	10, 20, 25, 30
TP7	MTG-L	10, 15, 20, 25, 30	MTG-L	10, 15, 20, 25, 30
T5	MTG-L	10, 15, 20, 25, 30	MTG-L	10, 15, 20, 25, 30
O1	MOG-L	10, 15, 20, 25, 30	MOG-L	10, 15, 20, 25, 30
O2	SOG-R	10, 15, 20	SOG-R	10, 15, 20, 25, 30
PO8	MOG-R	10, 15, 20, 25, 30	MOG-R	10, 15, 20, 25, 30
T6	MTG-R	10, 15, 20, 25, 30	MTG-R	10, 15, 20, 25, 30
TP8	MTG-R	10, 15, 20, 25, 30	MTG-R	10, 15, 20, 25, 30
FC5	IFGtriang-L	20, 25, 30	IFGoperc-L	10, 20, 30
FC3	MFG-L	10, 15, 20, 25, 30	MFG-L	10, 15, 20, 25, 30
FC1	MFG-L	10, 15, 20, 25, 30	MFG-L	15, 20, 25, 30
FC2	MFG-R	10, 15, 20, 25, 30	MFG-R	10, 15, 20, 25, 30
FC4	MFG-R	10, 15, 20, 25, 30	MFG-R	10, 15, 20, 25, 30
FC6	IFGoperc-R	10, 30	IFGoperc-R	10
F5	IFGtriang-L	10, 15, 20, 25, 30	IFGtriang-L	10, 15, 20, 25, 30
F3	MFG-L	10, 15, 20, 25, 30	MFG-L	10, 15, 20, 25, 30
F1	—	—	SFGdor-L	10, 15, 20, 25, 30
F2	SFGdor-R	10, 15	SFGdor-R	10, 15, 20, 25, 30
F4	MFG-R	10, 15, 20, 25, 30	MFG-R	10, 15, 20, 25, 30
F6	IFGtriang-R	10, 15, 20, 25, 30	IFGtriang-R	10, 15, 20, 25
AF3	MFG-L	25, 30	MFG-L	20, 25, 30
AF4	MFG-R	30	—	—
CP3	IPL-L	20	—	—
CP1	PoCG-L	10, 15, 20, 25, 30	PoCG-L	10, 15, 20, 25, 30
CP2	PoCG-R	10, 15, 20, 25, 30	PoCG-R	10, 15, 20, 25, 30
CP4	IPL-R	10, 15, 20, 25, 30	IPL-R	10, 15, 20, 25, 30
CP6	SMG-R	10, 15, 20, 25, 30	SMG-R	10, 15, 20, 25
P5	ANG-L	10, 15, 20, 25	ANG-L	10, 15, 20
P3	ANG-L	10, 15	ANG-L	10, 15, 20, 25, 30
P1	SPG-L	10, 15, 20, 25, 30	SPG-L	10, 15, 20, 25, 30
P2	SPG-R	10, 15, 20, 25, 30	SPG-R	10, 15, 20, 25, 30
P4	ANG-R	10, 15, 20, 25	ANG-R	10, 15, 20, 25, 30
P6	ANG-R	10, 15, 20, 25, 30	ANG-R	10, 15, 20, 25, 30
PO3	ANG-L	10, 15, 20, 25, 30	—	—

Note: the line ‘–’ indicates that the category is not applicable. See Table S2 in the Supplementary Material for abbreviations of brain regions.

### 3.3 Suitable SD Distances for infant fNIRS Based on SCC Metrics

Based on the results in Sec. 3.2, we found that the sensitivity and selectivity of the MLCBR increased and decreased, respectively, as the SD distance increased. In other words, there is a trade-off between the sensitivity and selectivity of the MLCBR in determining a suitable SD distance for targeting the brain regions of interest in infant fNIRS. Therefore, a suitable SD distance that achieves a good balance between the sensitivity and selectivity of the MLCBR in infant fNIRS was explored.

**Fig. 10:**
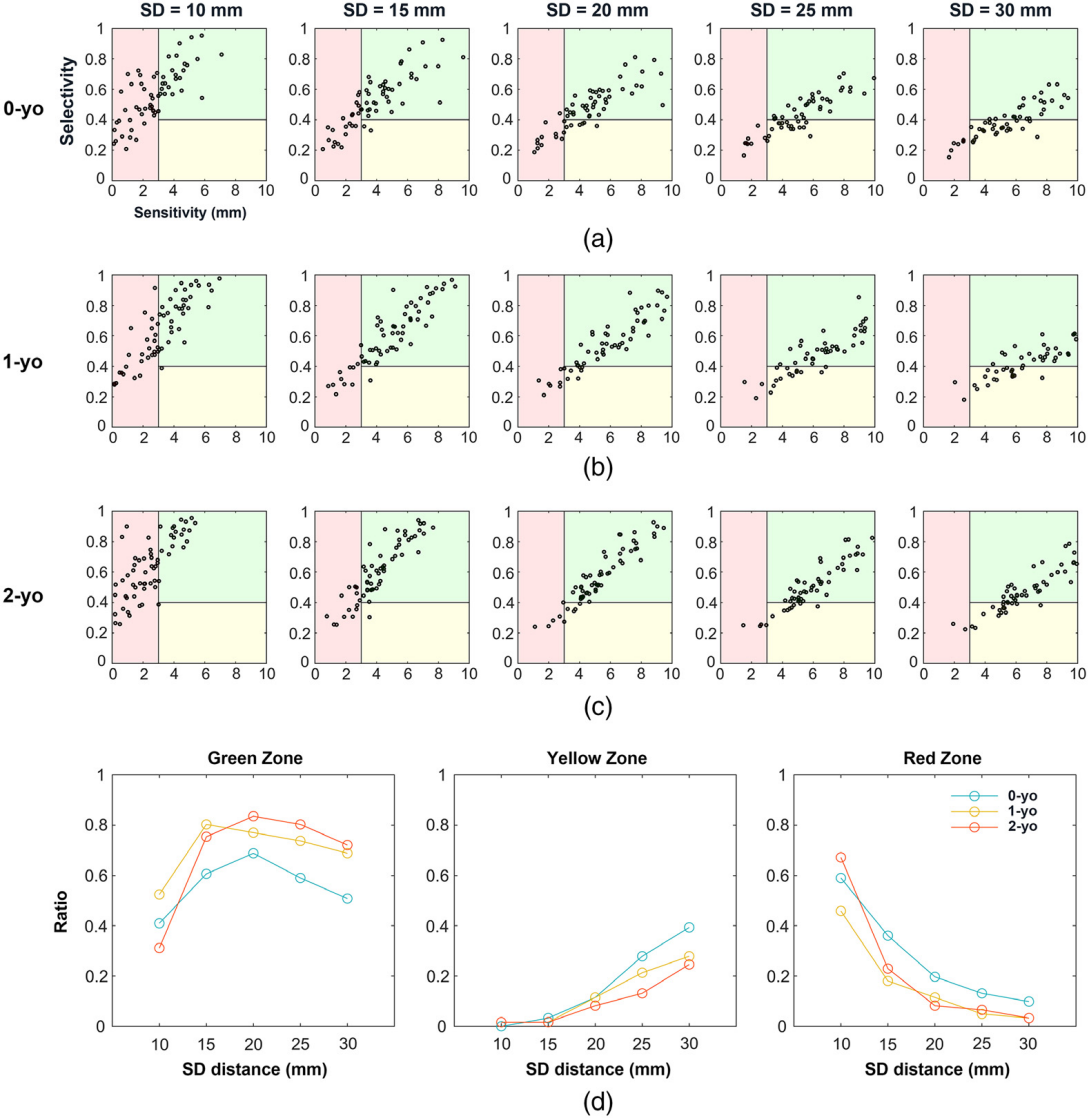
Relation between sensitivity and selectivity for each SD distance in (a) 0-yo; (b) 1-yo; and (c) 2-yo. Black circles in figures (a)–(c) indicate data from each 10-10 fiducial point. (d) Ratios of the number of fiducial points in the green, yellow, and red zones to the number of all 10-10 fiducial points.

First, we drew a scatter plot of the selectivity and sensitivity for all 10-10 fiducial points at five SD distances at each infant age [Figs. 10(a)–10(c)]. Then, we empirically chose thresholds 3 and 0.4 for the sensitivity and the selectivity, respectively, to categorize every point in the scatter plot into three zones. Because sensitivity takes precedence over selectivity in fNIRS, we defined the red zone as an area with a sensitivity <3. The remaining region was divided into two zones based on the selectivity threshold. Fiducial points with a sensitivity lower and >0.4, were classified into yellow and green zones, respectively. Therefore, the SD distance with a larger number of fiducial points categorized into the green zone indicates that both higher sensitivity and selectivity can be achieved.

As shown in Fig. 10, the distribution of points in the scatter plot varies with the SD distance for infants at all ages [Figs. 10(a)–10(c) for 0-, 1-, and 2-yo infants, respectively]. For shorter and longer SD distances, more fiducial points were observed in the red and yellow zones, respectively. The ratios of the number of fiducial points in each zone to all fiducial points are shown in Fig. 10(d). For the green zone, inverted U-shaped curves were observed for all three age groups. On the other hand, the ratio of fiducial points in the yellow and red zones showed a monotonic increase and decrease as the SD distance increased. These findings suggest that excessive short and long SD distances are unsuitable for infant fNIRS. Therefore, the SD distance between 15 mm and 25 mm could be suitable for 0-, 1-, and 2-yo infants. A 25 mm SD distance was more suitable if the sensitivity was emphasized, whereas a 15-mm SD distance was more appropriate if the selectivity was considered as a priority. A relatively balanced trade-off can be obtained when the SD distance is 20 mm. To validate the robustness of this finding, we examined the same problem with different thresholds 4 and 0.5 for sensitivity and selectivity, respectively. Although the thresholds changed modestly, the shapes of the graph, as shown in Fig. 10(d), were maintained (Fig. S5 in the Supplementary Material).

## 4 Discussion

fNIRS has contributed significantly to the advancement of developmental cognitive neuroscience; however, fNIRS data cannot provide any anatomical brain information, which is critical for data explanation and comparisons with other modalities. Although several methods have been proposed to obtain SCC in adults and infants, little is known about the influence of age and SD distance on SCC with substantial physical development of the infant’s head during the first two postnatal years. In the present study, we adopted light propagation analysis to establish a precise optics-based SCC between the fNIRS measurement channels, that is, SD pairs, set on the 10-10 system scalp positions and AAL brain regions in three age-appropriate head models of 0-, 1-, and 2-yo. Importantly, we provided four metrics: $N_{CBR}$, and the selectivity, sensitivity, and consistency of the MLCBR, to quantitatively evaluate the SCC for changes during a remarkable period of brain development. Moreover, we assessed the suitable SD distances for infant fNIRS by simultaneously considering the selectivity and sensitivity of the MLCBR.

### 4.1 Optics-Based SCC Derived from Age-Appropriate Sophisticated Head Models

Several studies have taken an important step toward the establishment of infant SCC;^22^^–^^25^ however, they only considered the head size of infants based on the simplified fNIRS principle that the signal comes from the cortical projection point below the midpoint of the SD pair. In fact, an fNIRS channel measures absorption changes in a broad cortical area, rather than at a single point. Thus, to establish a precise optics-based SCC that reflects light diffusion in the brains of 0-, 1-, and 2-yo infants, the key step is to construct sophisticated realistic head models that comprise multiple biological tissues with distinct optical properties such as scalp, skull, CSF, and brain tissues.

In this study, we constructed a five-layered head model for each age, distinguishable from a four-layered model where the skull and scalp constitute a single extracerebral layer.^36^^,^^46^^,^^59^ Separating the scalp and skull in infantile light propagation analysis could guarantee more accurate SCC compared with using the four-layered model, as these two types of infant tissues have their own optical properties^35^^,^^51^ and physical development.^43^^,^^44^

In addition, we employed template-based head models for light propagation analyses, because the acquisition of subject-specific MRI is generally challenging owing to the difficulty in controlling the motion of infants and undermines the intrinsic advantage of the fNIRS technique for facilitating functional brain measurements during early development. Notably, previous adult or infant studies have shown that the use of a template-based head model is useful for identifying the activation focus, although there are anatomical differences between subject-specific and template-based head models.^24^^,^^25^^,^^60^^–^^62^ Furthermore, the template-based model used in this study was derived from anatomical images of 95 healthy infants and reflects the general anatomical structure of infants. Therefore, the optics-based SCC obtained by analyzing light propagation in template-based infant head models of 0-, 1-, and 2-yo could provide a reliable database to guide fNIRS users in designing probe geometry, and to explain fNIRS data.

Light propagation analysis depends on the optical properties of head tissues. In this study, the same optical properties of the neonate were assigned to all three age models because no established optical properties of head tissues were available for each age group in this study. On the other hand, optical properties in these ages may change due to alterations in tissue composition, such as myelination and bone mineral density during the first 2 years of life. As the level of bone mineral density was almost constant during the first year^63^ and slightly increased in the second year,^64^ the optical properties of the skull may not change during those years. In contrast, a substantial amount of myelination occurs in a wide range of brains.^65^ Thus, the alteration of the optical properties of WM and GM will definitely be at a non-negligible level. Light propagation is affected by differences in optical properties between adults and infants.^35^ To address the effect of changes in optical properties on the SCC, we attempted to use the optical properties of the GM and WM of adults to calculate the SCC for the oldest 2-yo head model. Compared to the results with infants’ optical properties, additional simulation results revealed that the optical properties of adults applied to 2-yo had little effect on the SCC. Although a slightly broadened SSP was observed owing to the stronger scattering (Fig. S6 in the Supplementary Material for comparison between the optical properties of the neonate and adult), the consistency of the MLCBR among 0-, 1-, and 2-yo was unaffected by the difference in the optical properties (see Fig. S7 and Table S8 in the Supplementary Material for the optical properties of the adults). The actual changes in optical properties due to development from 0- to 2-yo would be smaller than those assumed here for neonates to adults. Therefore, we claim that our findings using the same optical properties for all three age groups could be used as a reference for developmental cognitive neuroscience. Meanwhile, the accuracy of optics-based SCC will be improved with the advance of in vivo measurement techniques for characterizing the optical properties of tissues.^66^ However, the measurement of accurate optical properties in vivo is still a challenging issue, as shown by the wide range of values reported by measurement techniques.^36^

### 4.2 Effect of SD Distance and Physical Development on the SCC

For a given fiducial point, the SSP was found to be a function of the SD distance, that is, the spatial range of the SSP broadened as the SD distance increased. This finding was consistent with previous studies, regardless of slab models^30^^,^^67^ or realistic head models^68^ being utilized. Intriguingly, the spatial distribution of the SSP values was also affected by age for a consistent SD distance (Fig. 4). Age-related alterations in SSP may be caused by changes in the local anatomical structures across the three age groups.

The optics-based SCC results (Table 4 and Table. S3-7 in the Supplementary Material) demonstrate that fNIRS signals interrogated by an SD pair always originate from multiple brain regions, even if the SD distance is 10 mm. The one-to-many relationship between an SD pair and cortical regions is due to strong light scattering in the head tissue. In addition, we found several aspects related to $N_{CBR}$. As for the spatial characteristics, fiducial points around the longitudinal fissure were correlated with more brain regions than other points (Fig. 5), which may be because the cortices around the longitudinal fissure were parcellated into a relatively large number of regions in the AAL atlas. From the perspective of age-related change, we found that although the $N_{CBR}$ of 0-yo was larger than that of 1- and 2-yo, there were no significant differences between the $N_{CBR}$ values of 1-o and 2-yo [Fig. 8(d)], which suggests that the similar spatial range of SSP for the same SD distance will occupy fewer brain regions when the head becomes larger owing to the physical development of the infant. In terms of the dependency on the SD distance, the finding that a larger SD distance had more corresponding brain regions [Figs. 5, 8(a), and 8(d)] could be clearly explained by the wider SSP at larger SD distances, as shown in Fig. 4.

We found that the selectivity of MLCBR decreased with increasing SD distance [Figs. 6 and 8(b)]. As mentioned before, the larger the SD distance, the wider the spatial range of the SSP in the brain. Hence, the increase in the normalized PPL of brain regions other than the MLCBR resulted in a relative decrease in the normalized PPL of the MLCBR. In addition, the selectivity of 1- and 2-yo was higher than that of 0-yo on average for all fiducial points (Fig. 6). The difference in the physical development of the head in each year would have determined the degree of age-dependent change in the normalized PPL of the MLCBR. The 0-yo head size is obviously smaller than that of 1- and 2-yo, while 1- and 2-yo have an almost similar head size (see Fig. 1 for example). In other words, the constant area of the brain surface is occupied by a small number of brain regions in the large brains of 1- and 2-yo, but by a large number in the small brains of 0-yo. Furthermore, the spatial range of the SSP was almost constant for the same SD distance, regardless of age. Thus, the constant spatial range of SSP across age was mainly occupied by the MLCBR in the larger brains of 1- and 2-yo, but distributed across several brain regions in the smaller brain of the 0-yo. Therefore, the normalized PPL in the MLCBR of the 0-yo infants was significantly smaller than that of the 1- and 2-yo infants.

We found that the sensitivity of the MLCBR significantly increased as the SD distance increased from 10 to 30 mm at 5-mm intervals for each age [Fig. 8(f)]. This finding is in accordance with evidence obtained from the change in GM sensitivity as a function of SD distance in the neonatal and adult head models.^35^ The sensitivity of 1-yo was significantly higher than that of 0-yo at 15-, 20-, 25-, and 30-mm SD distances; however, the sensitivity of 2-yo was significantly lower than that of 1-yo at a 10 mm SD distance [Fig. 8(f)]. One possible explanation for the increase or decrease in the sensitivity depending on the time of growth is that physical development differs between brain tissue and superficial layers such as the scalp and skull; the increase in the sensitivity from 0- to 1-yo may be caused by the considerable expansion of the cortical surface during this period. Conversely, from 1- to 2-yo, the decrease in sensitivity may be due to relatively smaller expansion of the cortical surface, although the thickness of the superficial layers continues to increase.

We also found that the MLCBR was completely consistent at almost half of 10-10 fiducial points of 0-, 1-, and 2-yo for each SD distance. In other words, almost half of the fiducial points were correlated with one specific brain region with the largest normalized PPL (Fig. 9 and Table 4). This finding indicates that we can measure identical MLCBRs in longitudinal studies from 0- to 2-yo with fNIRS without the subject’s own structural MRI when a probe of the same SD distance is attached at the scalp fiducial points. Thus, this finding is extremely uplifting for fNIRS users who are interested in the functional development of such MLCBRs. On the other hand, the MLCBR corresponding to fiducial points just above the longitudinal fissure was mostly inconsistent across the three ages (Fig. 9). The $N_{CBR}$ and $L_{norm,MLCBR}$ of the fiducial points were relatively larger and smaller than those of the other points, respectively (Figs. 5 and 6), which suggests that the MLCBR at a certain age may easily be replaced by another brain region at another age because of the slight change in the light path. One possibility for age-related changes in the light path is that the thickness of the skull in the frontal, parietal, and occipital regions along with the longitudinal fissure changes more drastically than in other locations from 0- to 2-yo.^44^ Another possibility is that the cortical surface expands relatively more in regions of the superior parietal, prefrontal, occipital cortices, and postcentral gyrus than in other regions during the first two years.^2^

The consistency of the MLCBR revealed in the present study could provide strong support for the reliability of findings from longitudinal developmental studies using fNIRS in which the probe was attached based on scalp fiducial points. In recent years, by executing the longitudinal cohort study projects [e.g., Brain Imaging for Global Health (BRIGHT) project], several laboratories worldwide have devoted themselves to investigating functional brain development during the first two postnatal years, including studies of visual working memory,^69^ social cognition,^70^^,^^71^ and resting-state functional networks.^72^ The present study will be especially useful for a large cohort of longitudinal or cross-sectional studies of early brain development, as structural MRI scanning, which is a demanding task for young populations, is not necessarily required.

### 4.3 Consideration of Suitable SD Distances for Infant fNIRS

The suitable SD distance for infants remains a matter of debate, as its choice is dependent on several factors. By considering a trade-off between the selectivity and sensitivity of the MLCBR, this study provided recommendations on suitable SD distances for 0-, 1-, and 2-yo. The most desirable SD distance is the one that has both the maximum number of fiducial points with high selectivity and sensitivity, and a minimum number of fiducial points with low selectivity and sensitivity. Based on this perspective, we determined that the suitable SD distances in fNIRS for infants during the first 2 years ranged from 15 to 25 mm. This finding supports the results of a previous study in which the highest sensitivity was obtained at an SD distance of 20 mm in 3-month-old infants.^39^ In practical applications, we suggest that fNIRS users choose the SD distance according to the criteria (sensitivity or selectivity) more important to them. If the users focus on selectivity, an SD distance of 15 mm could be a better choice, whereas 25 mm is suitable for sensitivity as the priority. In addition, if fNIRS users pursue a relatively balanced trade-off between selectivity and sensitivity, we recommend an SD distance of 20 mm. Our findings also revealed that the optimal SD distance for infants is different from that for adults. Previous adult studies have demonstrated that fNIRS probes should ideally be designed with 30- to 35-mm SD distances,^68^^,^^73^ as most light passes through the extra-cerebral superficial tissues at SD distances <20  mm.^67^ Compared with that for adults, and the most suitable SD distances for infants are smaller than 30 mm, which indicates that thin superficial layers allow much more photons to pass through the brain tissue.

### 4.4 Future Directions and Limitations

In the current study, we found that the orientation of SD pairs over 10-10 positions had no significant effect on our results, which was consistent with findings from photon propagation in the adult brain.^34^^,^^38^ Notably, only the AAL atlas was adopted to obtain the SCC in this study. The orientation of SD pairs may influence the SCC when other infant brain atlases with finer anatomical parcellation are available with advances in MRI techniques.

For the application of this work, the precise optics-based SCC for SD pairs at all 10-10 system scalp locations (Table S3-7 in the Supplementary Material) can be used as a reference table to guide fNIRS users in designing the probe arrangement for targeting specific brain regions, as well as to help them explain fNIRS data obtained according to the 10-10 system. Importantly, the summary of scalp positions whose MLCBR was completely consistent across 0-, 1-, and 2-yo infants (Table 4) is beneficial for the investigation of functional development for one specific brain region. However, the spatial density of the 10-10 fiducial points for analyzing the optics-based SCC would be relatively sparse because a few brain regions had only a small chance of being corresponded. Our future work will calculate the optics-based SCC by using a denser placement system,^74^ which can guarantee all brain regions with a high probability of being corresponded and estimate underlying brain regions for any arrangement of SD pairs. In addition, the established age-appropriate infant head models and the proposed method for calculating the optics-based SCC can be used to estimate brain regions at any scalp position by virtually placing the SD pairs on the scalp of 0-, 1-, and 2-yo infants, even without infant head structures.

The head tissues within the first postnatal year undergo dramatic growth, such as the total volume of the brain expanding up to double its size.^44^^,^^75^^,^^76^ Furthermore, several studies have reported the discovery of longitudinal functional brain development during this period.^71^^,^^77^^,^^78^ However, the 0-yo infant head model used in this study was constructed with a template-based MRI of an approximately 1-month-old infant. Establishing optics-based SCC at a fine temporal scale (every 3 months from birth to 12 months), which is in line with physical development during the first year after birth, it is expected to provide useful information for investigating the longitudinal development of brain functions using the fNIRS technique.

## 5 Conclusion

The present study adopted light propagation analysis to establish a precise optics-based SCC between fNIRS measurement channels (i.e., SD pairs) set on the 10-10 system scalp positions and AAL brain regions in three age-appropriate head models of 0-, 1-, and 2-yo. We found that age-related changes in the SCC metrics, particularly the fiducial points around the longitudinal fissure, correlated with different brain regions across the three age groups. In contrast, the MLCBR was consistent across the three ages for more than half of the fiducial points. Furthermore, we recommend SD distances between 15 and 25 mm as suitable for fNIRS in infants by simultaneously considering the selectivity and sensitivity of the MLCBR. We sincerely hope that age-appropriate SCC will be a useful reference to guide the design of fNIRS probes and provide convincing anatomical interpretations of fNIRS data for future infant developmental studies.

## Code, Data, and Materials Availability

Data supporting the results reported in the paper can be requested by contacting the corresponding author.
