# Supplementary material for: Correlating functional near-infrared spectroscopy with underlying cortical regions of 0-, 1-, and 2-year-olds using theoretical light propagation analysis
Source: Neurophotonics. 2021 May 31;8(2):025009. doi: 10.1117/1.NPh.8.2.025009 (PMC8166262; doi:10.1117/1.NPh.8.2.025009)
Supplement: Supplementary file 1 [file NPh_008_025009_SD001.pdf]

Supplementary materials

Fig. S1. PMDF for a given fiducial point T4 at 5 SD distances from 0-yo, 1-yo, and 2-yo when the SD pair was attached in a vertical orientation. Dashed lines in different colors indicate the boundaries of AAL brain regions. The PMDF superimposed on age-appropriate brain structures from 0-yo, 1-yo, and 2-yo are shown in (a), (b) and (c), respectively.

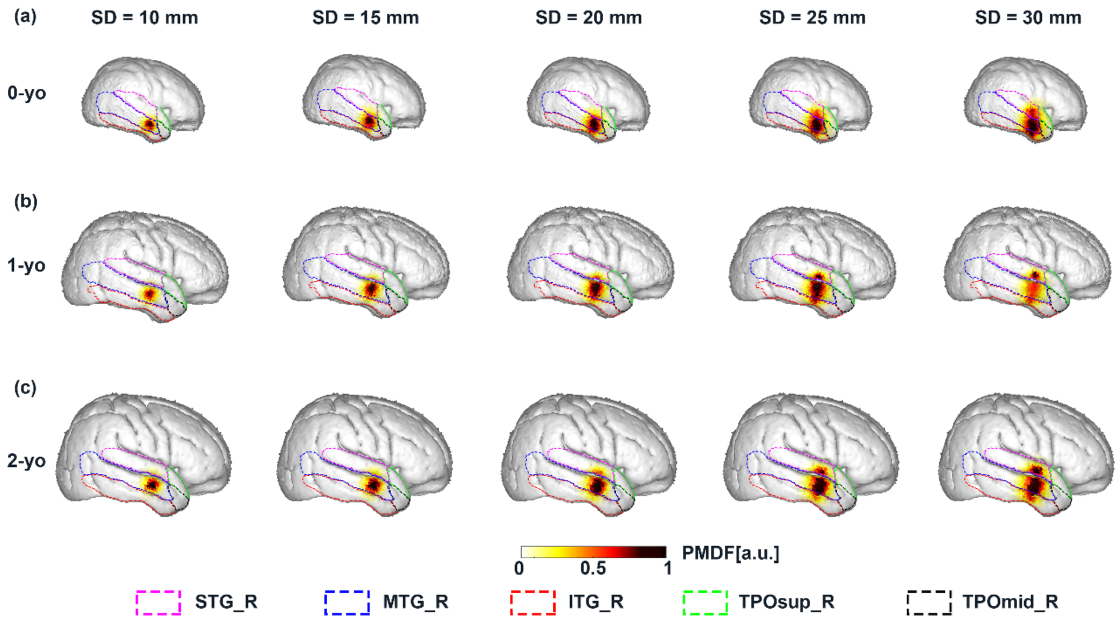

Fig. S2. The number of the corresponding brain region (N_{CBR}) for vertical SD pairs at all 10-10 fiducial points at 5 different SD distances for 0-yo (a), 1-yo (b) and 2-yo (c). The darker red indicates larger N_{CBR}. (d) Box plots of N_{CBR} at all scalp fiducial points of the 10-10 system for each condition of the age and the SD distance. The individual colored dots indicate the N_{CBR} of each fiducial point. Boxes indicate the interquartile range. The black horizontal line within the boxes indicates the median. Whiskers extend 1.5 times above and below the interquartile range limits. (e) Statistical significance of post-hoc test for N_{CBR} is indicated by matrices. * $p < 0.05$, ** $p < 0.01$, *** $p < 0.001$, white and gray blank are not significant and not applicable, respectively.

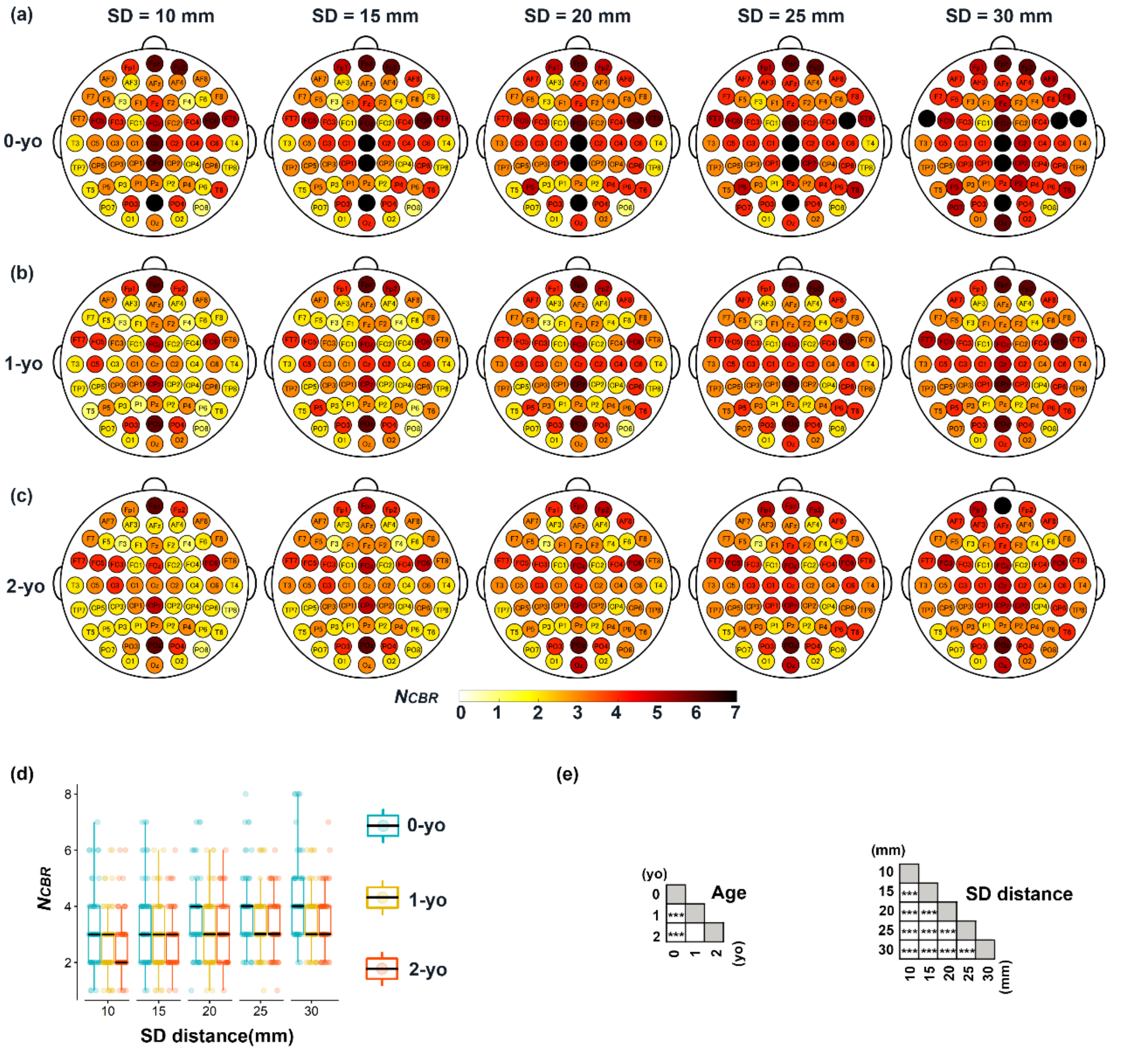

Fig. S3. Selectivity of the MLCBR for vertical SD pairs at all 10-10 fiducial points at 5 different SD distances for 0-yo (a), 1-yo (b) and 2-yo (c). Magenta and blue indicate higher and lower selectivity, respectively. (d) Box plots of the selectivity at all scalp fiducial points of the 10-10 system for each condition of the age and the SD distance. The individual colored dots indicate the selectivity of each fiducial point. Boxes indicate the interquartile range. The black horizontal line within the boxes indicates the median. Whiskers extend 1.5 times above and below the interquartile range limits. (e) Statistical significance of post-hoc test for the selectivity is indicated by matrices. * $p < 0.05$, ** $p < 0.01$, *** $p < 0.001$, white and gray blank are not significant and not applicable, respectively.

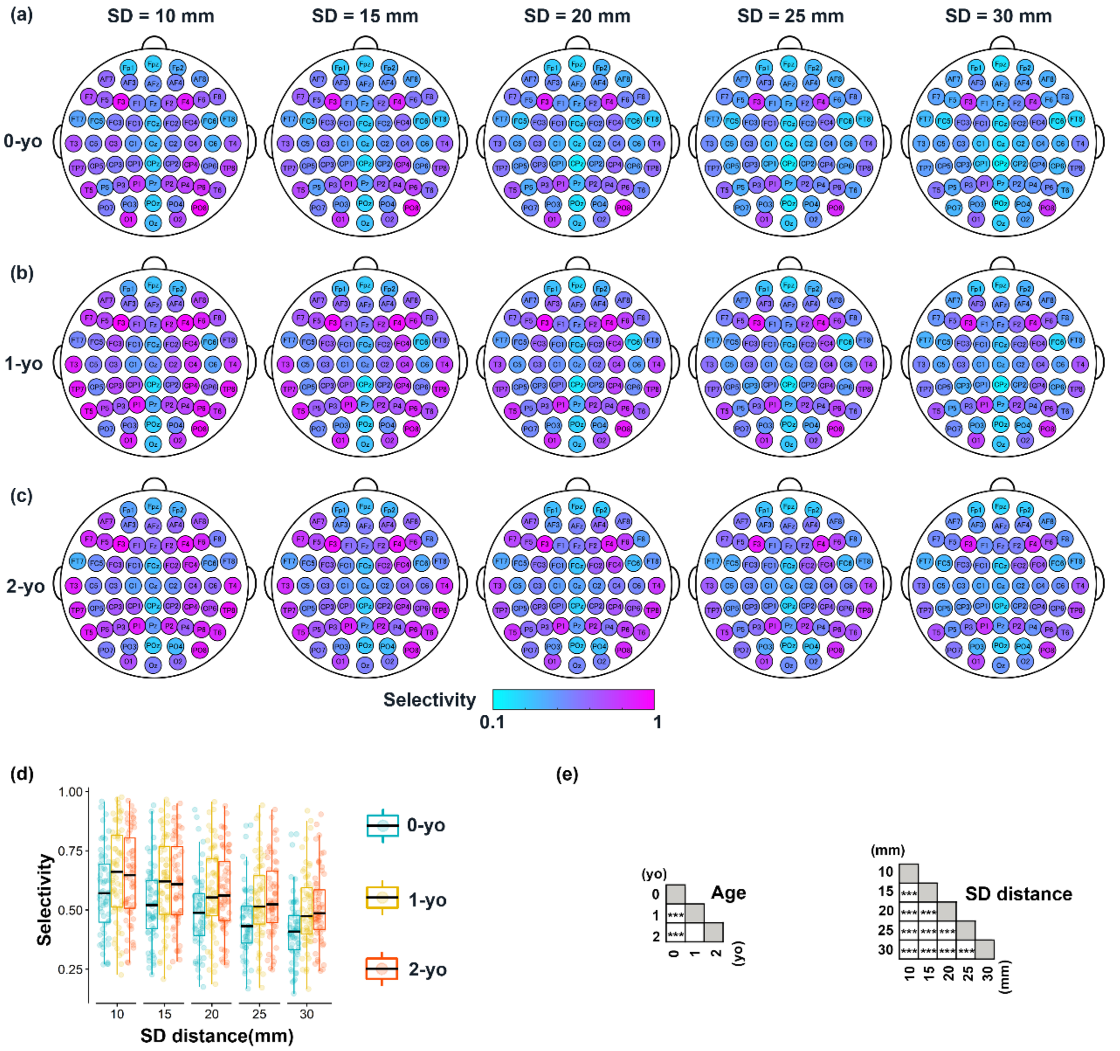

Fig. S4. Sensitivity of the MLCBR for vertical SD pairs at all 10-10 fiducial points at 5 different SD distances for 0-yo (a), 1-yo (b) and 2-yo (c). Magenta and blue indicate higher and lower sensitivity, respectively. (d) Box plots of the sensitivity at all scalp fiducial points of the 10-10 system for each condition of the age and the SD distance. The individual colored dots indicate the sensitivity of each fiducial point. Boxes indicate the interquartile range. The black horizontal line within the boxes indicates the median. Whiskers extend 1.5 times above and below the interquartile range limits. (e) Statistical significance of post-hoc test for the sensitivity is indicated by matrices. * $p < 0.05$, ** $p < 0.01$, *** $p < 0.001$, white and gray blank are not significant and not applicable, respectively.

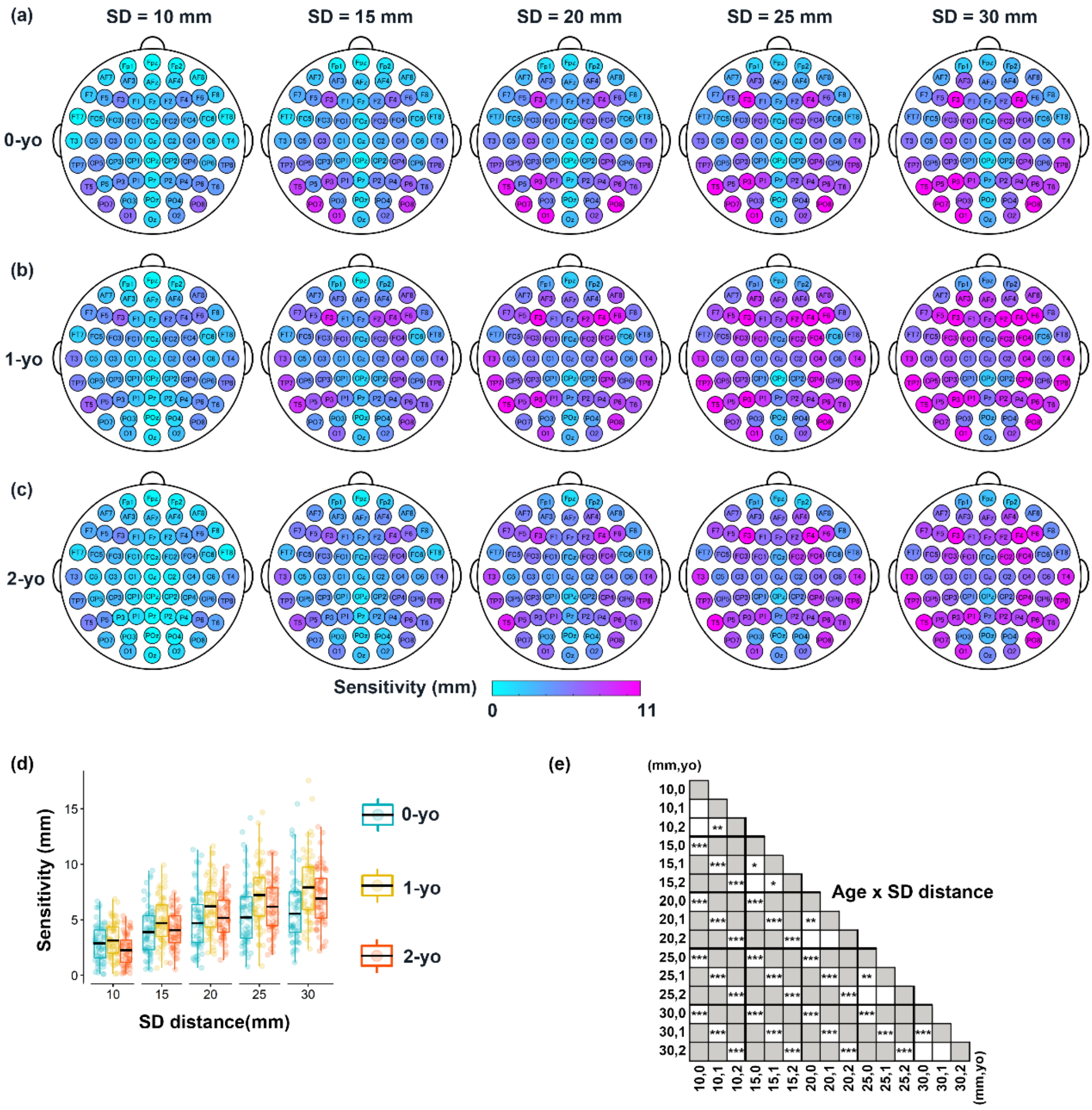

Fig. S5. Relation between the sensitivity and the selectivity for each SD distance in 0-yo (a), 1-yo (b), and 2-yo (c) at thresholds of 4 mm and 0.5 for the sensitivity and the selectivity, respectively. Black circles in subfigures a-c indicate data from each 10-10 fiducial point. (d) Ratios of the number of fiducial points in the green, yellow and red zones to the number of all 10-10 fiducial points.

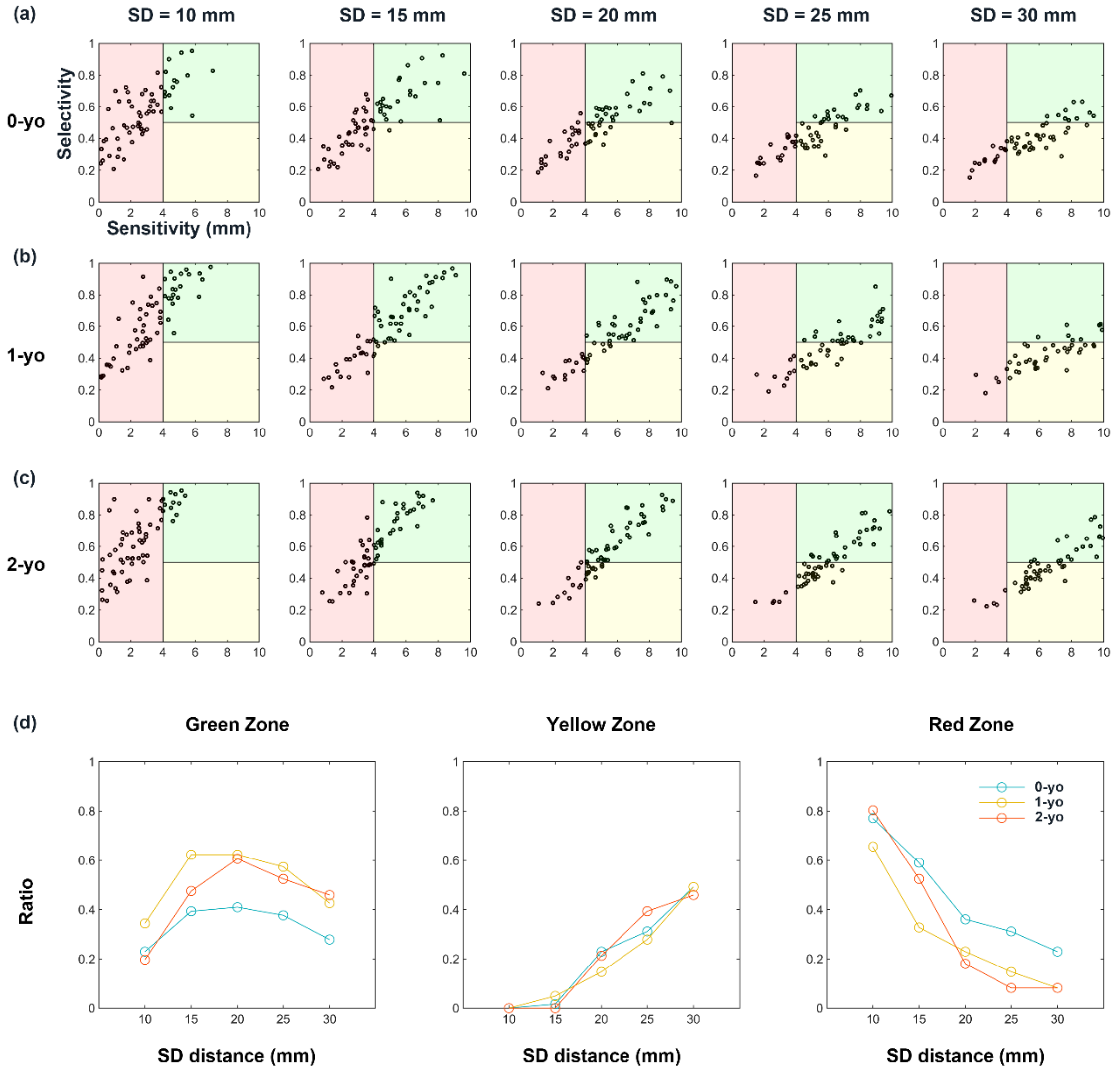

Fig. S6 Comparison of the PMDF obtained from neonate' (a) and adult's optical properties (b) for the fiducial point T4 at five SD distances for 2-yo when the SD pair was attached in a circumferential orientation. Dashed lines in different colors indicate the AAL brain region boundaries. Adult's absorption coefficients are 0.019 mm^{-1} and 0.011 mm^{-1} for GM and WM, respectively. Adult's reduced scattering coefficients are 0.86 mm^{-1} and 4.16 mm^{-1} for of GM and WM, respectively.

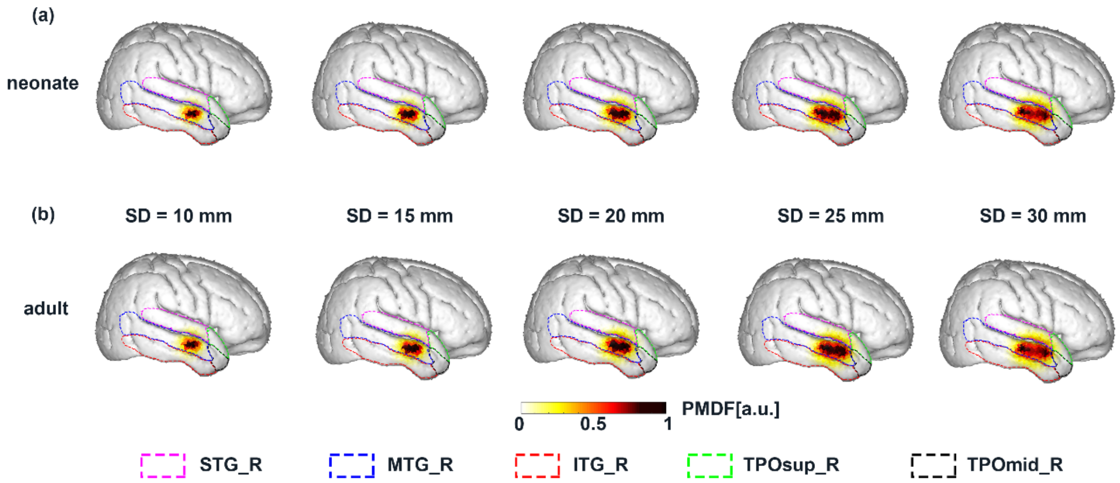

Fig. S7 Consistency of MLCBR at five SD distances for circumferential (a) and vertical (b) SD pairs. The consistency of the MLCBR among 0-yo, 1-yo, and 2-yo for every fiducial point is indicated by circles with different colors. Note: MLCBR for 2-yo was obtained by adult's optical properties of GM and WM. Adult's absorption coefficients are 0.019 mm^{-1} and 0.011 mm^{-1} for GM and WM, respectively. Adult's reduced scattering coefficients are 0.86 mm^{-1} and 4.16 mm^{-1} for GM and WM, respectively.

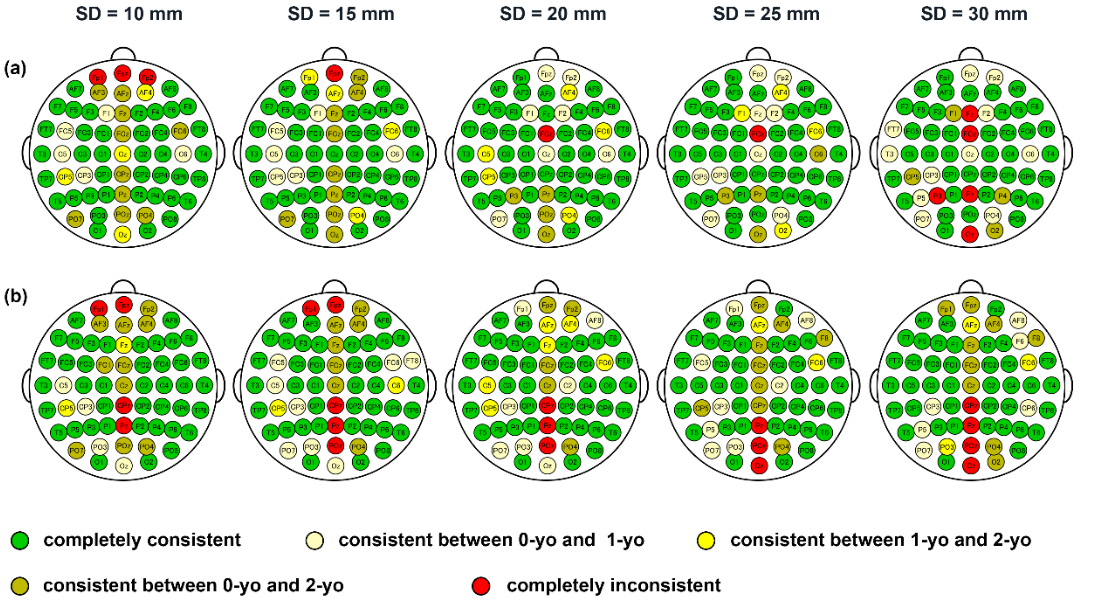

Table S1 List of abbreviations.

Abbreviation	Definition
fNIRS	functional near-infrared spectroscopy
0-yo	0-year-old
1-yo	1-year-old
2-yo	2-year-old
SD	source-detector
SCC	scalp-cortex correlation
PPL	partial pathlength
SSP	spatial sensitivity profile
GM	gray matter
WM	white matter
MRI	magnetic resonance imaging
CSF	cerebrospinal fluid
AAL	automated anatomical labeling
PMDF	photon measurement density function
MLCBR	most likely corresponding brain region
WTS	Wald-type statistic

Table S2 AAL brain regions.

Index	AAL region	Abbreviation	Index	AAL region	Abbreviation
1	Precentral gyrus left	PreCG-L	46	Cuneus right	CUN-R
2	Precentral gyrus right	PreCG-R	47	Lingual gyrus left	LING-L
3	Superior frontal gyrus (dorsal) left	SFGdor-L	48	Lingual gyrus right	LING-R
4	Superior frontal gyrus (dorsal) right	SFGdor-R	49	Superior occipital gyrus left	SOG-L
5	Orbitofrontal cortex (superior) left	ORBsup-L	50	Superior occipital gyrus right	SOG-R
6	Orbitofrontal cortex (superior) right	ORBsup-R	51	Middle occipital gyrus left	MOG-L
7	Middle frontal gyrus left	MFG-L	52	Middle occipital gyrus right	MOG-R
8	Middle frontal gyrus right	MFG-R	53	Inferior occipital gyrus left	IOG-L
9	Orbitofrontal cortex (middle) left	ORBmid-L	54	Inferior occipital gyrus right	IOG-R
10	Orbitofrontal cortex (middle) right	ORBmid-R	55	Fusiform gyrus left	FFG-L
11	Inferior frontal gyrus (opercular) left	IFGoperc-L	56	Fusiform gyrus right	FFG-R
12	Inferior frontal gyrus (opercular) right	IFGoperc-R	57	Postcentral gyrus left	PoCG-L
13	Inferior frontal gyrus (triangular) left	IFGtriang-L	58	Postcentral gyrus right	PoCG-R
14	Inferior frontal gyrus (triangular) right	IFGtriang-R	59	Superior parietal gyrus left	SPG-L
15	Orbitofrontal cortex (inferior) left	ORBinf-L	60	Superior parietal gyrus right	SPG-R
16	Orbitofrontal cortex (inferior) right	ORBinf-R	61	Inferior parietal lobule left	IPL-L
17	Rolandic operculum left	ROL-L	62	Inferior parietal lobule right	IPL-R
18	Rolandic operculum right	ROL-R	63	Supramarginal gyrus left	SMG-L
19	Supplementary motor area left	SMA-L	64	Supramarginal gyrus right	SMG-R
20	Supplementary motor area right	SMA-R	65	Angular gyrus left	ANG-L
21	Olfactory left	OLF-L	66	Angular gyrus right	ANG-R
22	Olfactory right	OLF-R	67	Precuneus gyrus left	PCUN-L
23	Superior frontal gyrus (medial) left	SFGmed-L	68	Precuneus gyrus right	PCUN-R
24	Superior frontal gyrus (medial) right	SFGmed-R	69	Paracentral lobule left	PCL-L
25	Orbitofrontal cortex (medial) left	ORBmed-L	70	Paracentral lobule right	PCL-R
26	Orbitofrontal cortex (medial) right	ORBmed-R	71	Caudate left	CAU-L
27	Rectus gyrus left	REC-L	72	Caudate right	CAU-R
28	Rectus gyrus right	REC-R	73	Putamen left	PUT-L
29	Insula left	INS-L	74	Putamen right	PUT-R
30	Insula right	INS-R	75	Pallidum left	PAL-L
31	Anterior cingulate gyrus left	ACG-L	76	Pallidum right	PAL-R
32	Anterior cingulate gyrus right	ACG-R	77	Thalamus left	THA-L

33	Middle cingulate gyrus left	MCG-L	78	Thalamus right	THA-R
34	Middle cingulate gyrus right	MCG-R	79	Heschl gyrus left	HES-L
35	Posterior cingulate gyrus left	PCG-L	80	Heschl gyrus right	HES-R
36	Posterior cingulate gyrus right	PCG-R	81	Superior temporal gyrus left	STG-L
37	Hippocampus left	HIP-L	82	Superior temporal gyrus right	STG-R
38	Hippocampus right	HIP-R	83	Temporal temporal gyrus left	TPOsup-L
39	ParaHippocampal gyrus left	PHG-L	84	Temporal temporal gyrus right	TPOsup-R
40	ParaHippocampal gyrus right	PHG-R	85	Middle temporal gyrus left	MTG-L
41	Amygdala left	AMYG-L	86	Middle temporal gyrus right	MTG-R
42	Amygdala right	AMYG-R	87	Temporal pole (middle) left	TPOmid-L
43	Calcarine cortex left	CAL-L	88	Temporal pole (middle) right	TPOmid-R
44	Calcarine cortex right	CAL-R	89	Inferior temporal gyrus left	ITG-L
45	Cuneus left	CUN-L	90	Inferior temporal gyrus right	ITG-R

Table S3 Normalized PPL of corresponding brain regions ($L_{norm,M}$) for the circumferential and vertical SD pairs set at 10/10 fiducial points at 10-mm SD distance from 0-yo, 1-yo, and 2-yo.

SD distance = 10 mm		Circumferential SD pair			Vertical channel SD pair		
10/10 Points	AAL regions	0 year	1 year	2 year	0 year	1 year	2 year
Cz	PreCG-R	0.019	0.003	0.004	0.012	0.003	0.003
Cz	PreCG-L	0.052	0.016	0.009	0.066	0.019	0.009
Cz	SFGdor-R	0.088	0.025	0.038	0.045	0.018	0.031
Cz	SFGdor-L	0.147	0.134	0.072	0.145	0.112	0.061
Cz	SMA-R	0.268	0.346	0.441	0.176	0.292	0.462
Cz	SMA-L	0.228	0.434	0.405	0.354	0.502	0.397
Cz	PoCG-L	0.060	0.002	0.001	0.060	0.003	0.001
Cz	SPG-L	0.028	0.000	0.000	0.025	0.000	0.000
Cz	PCL-R	0.064	0.029	0.021	0.057	0.038	0.024
Cz	PCL-L	0.020	0.007	0.005	0.029	0.011	0.007
Fpz	SFGdor-R	0.038	0.017	0.021	0.028	0.012	0.023
Fpz	SFGdor-L	0.075	0.054	0.014	0.061	0.052	0.011
Fpz	ORBsup-R	0.028	0.022	0.046	0.023	0.017	0.050
Fpz	ORBsup-L	0.075	0.117	0.070	0.071	0.104	0.056
Fpz	ORBmid-L	0.016	0.008	0.008	0.015	0.008	0.007
Fpz	SFGmed-R	0.212	0.105	0.076	0.202	0.099	0.095
Fpz	SFGmed-L	0.243	0.191	0.087	0.272	0.228	0.082
Fpz	ORBmed-R	0.134	0.183	0.316	0.132	0.156	0.351
Fpz	ORBmed-L	0.149	0.286	0.323	0.167	0.308	0.277
Fpz	REC-R	0.009	0.010	0.025	0.010	0.010	0.033
AFz	SFGdor-R	0.040	0.019	0.062	0.041	0.014	0.050
AFz	SFGdor-L	0.054	0.102	0.038	0.057	0.093	0.029
AFz	SFGmed-R	0.302	0.394	0.542	0.395	0.313	0.597
AFz	SFGmed-L	0.585	0.474	0.349	0.483	0.568	0.314
Fz	SFGdor-R	0.045	0.013	0.059	0.054	0.018	0.058
Fz	SFGdor-L	0.068	0.095	0.046	0.068	0.069	0.030
Fz	SMA-R	0.036	0.000	0.000	0.019	0.001	0.000
Fz	SMA-L	0.081	0.000	0.000	0.026	0.001	0.000
Fz	SFGmed-R	0.277	0.237	0.526	0.474	0.330	0.598
Fz	SFGmed-L	0.482	0.651	0.363	0.345	0.578	0.309

SD distance = 10 mm		Circumferential SD pair			Vertical channel SD pair		
10/10 Points	AAL regions	0 year	1 year	2 year	0 year	1 year	2 year
FCz	SFGdor-R	0.077	0.016	0.051	0.049	0.013	0.045
FCz	SFGdor-L	0.091	0.039	0.033	0.094	0.056	0.029
FCz	SMA-R	0.283	0.357	0.215	0.220	0.157	0.236
FCz	SMA-L	0.250	0.302	0.118	0.313	0.376	0.111
FCz	SFGmed-R	0.100	0.107	0.314	0.103	0.113	0.326
FCz	SFGmed-L	0.133	0.177	0.263	0.198	0.282	0.248
FCz	PCL-R	0.046	0.000	0.000	0.004	0.000	0.000
CPz	PreCG-L	0.043	0.018	0.016	0.047	0.017	0.018
CPz	SMA-L	0.013	0.009	0.009	0.014	0.010	0.013
CPz	PoCG-R	0.037	0.009	0.017	0.021	0.007	0.013
CPz	PoCG-L	0.290	0.290	0.155	0.269	0.217	0.127
CPz	SPG-R	0.014	0.006	0.008	0.010	0.005	0.006
CPz	SPG-L	0.051	0.040	0.022	0.076	0.040	0.021
CPz	PCUN-R	0.185	0.147	0.213	0.129	0.136	0.191
CPz	PCUN-L	0.062	0.125	0.094	0.134	0.184	0.102
CPz	PCL-R	0.158	0.152	0.258	0.101	0.150	0.279
CPz	PCL-L	0.114	0.199	0.200	0.172	0.225	0.219
Pz	PoCG-L	0.011	0.004	0.004	0.016	0.005	0.004
Pz	SPG-R	0.031	0.011	0.017	0.015	0.007	0.013
Pz	SPG-L	0.398	0.358	0.188	0.401	0.364	0.155
Pz	PCUN-R	0.319	0.272	0.449	0.227	0.218	0.455
Pz	PCUN-L	0.221	0.349	0.337	0.321	0.400	0.369
POz	CUN-R	0.158	0.081	0.101	0.132	0.083	0.112
POz	CUN-L	0.162	0.256	0.142	0.184	0.257	0.144
POz	SOG-R	0.152	0.070	0.094	0.069	0.057	0.079
POz	SOG-L	0.054	0.037	0.012	0.059	0.031	0.011
POz	MOG-L	0.010	0.002	0.001	0.011	0.002	0.001
POz	SPG-R	0.018	0.018	0.026	0.011	0.015	0.020
POz	SPG-L	0.208	0.279	0.243	0.272	0.271	0.225
POz	PCUN-R	0.153	0.164	0.265	0.144	0.169	0.276
POz	PCUN-L	0.070	0.090	0.112	0.105	0.112	0.130
Oz	CAL-R	0.038	0.020	0.031	0.037	0.024	0.037

SD distance = 10 mm		Circumferential SD pair			Vertical channel SD pair		
10/10 Points	AAL regions	0 year	1 year	2 year	0 year	1 year	2 year
Oz	CUN-R	0.376	0.313	0.546	0.284	0.317	0.547
Oz	CUN-L	0.207	0.263	0.209	0.277	0.306	0.227
Oz	SOG-R	0.044	0.030	0.057	0.022	0.028	0.042
Oz	SOG-L	0.297	0.348	0.138	0.342	0.298	0.128
Oz	MOG-L	0.022	0.016	0.005	0.021	0.014	0.005
T3	STG-L	0.025	0.058	0.033	0.035	0.073	0.041
T3	TPOsup-L	0.011	0.005	0.004	0.012	0.004	0.003
T3	MTG-L	0.700	0.898	0.901	0.712	0.882	0.887
T3	TPOmid-L	0.023	0.005	0.005	0.021	0.004	0.004
T3	ITG-L	0.238	0.034	0.057	0.218	0.035	0.063
C5	PreCG-L	0.014	0.015	0.015	0.014	0.009	0.010
C5	ROL-L	0.097	0.098	0.067	0.072	0.061	0.039
C5	PoCG-L	0.196	0.567	0.524	0.279	0.558	0.530
C5	SMG-L	0.042	0.076	0.083	0.036	0.069	0.075
C5	STG-L	0.603	0.238	0.301	0.545	0.299	0.337
C5	TPOsup-L	0.015	0.001	0.003	0.012	0.001	0.002
C5	MTG-L	0.024	0.002	0.004	0.033	0.002	0.005
C3	PreCG-L	0.338	0.468	0.363	0.239	0.318	0.335
C3	MFG-L	0.016	0.038	0.053	0.012	0.030	0.058
C3	PoCG-L	0.567	0.473	0.526	0.693	0.625	0.554
C3	SMG-L	0.074	0.020	0.054	0.051	0.026	0.051
C1	PreCG-L	0.473	0.474	0.440	0.454	0.446	0.434
C1	SFGdor-L	0.279	0.353	0.275	0.331	0.383	0.325
C1	MFG-L	0.200	0.144	0.259	0.153	0.143	0.212
C1	PoCG-L	0.037	0.023	0.022	0.049	0.021	0.024
C2	PreCG-R	0.539	0.695	0.499	0.518	0.696	0.487
C2	SFGdor-R	0.237	0.161	0.273	0.310	0.212	0.312
C2	MFG-R	0.127	0.105	0.189	0.080	0.051	0.165
C2	SMA-R	0.011	0.005	0.009	0.012	0.005	0.010
C2	PoCG-R	0.069	0.032	0.026	0.065	0.033	0.022
C4	PreCG-R	0.187	0.083	0.262	0.128	0.082	0.275
C4	MFG-R	0.013	0.002	0.012	0.009	0.002	0.013

SD distance = 10 mm		Circumferential SD pair			Vertical channel SD pair		
10/10 Points	AAL regions	0 year	1 year	2 year	0 year	1 year	2 year
C4	PoCG-R	0.569	0.793	0.660	0.622	0.828	0.658
C4	IPL-R	0.097	0.088	0.036	0.060	0.048	0.023
C4	SMG-R	0.129	0.033	0.028	0.178	0.040	0.030
C6	PreCG-R	0.008	0.002	0.012	0.010	0.002	0.014
C6	ROL-R	0.054	0.019	0.019	0.041	0.015	0.021
C6	PoCG-R	0.288	0.457	0.598	0.372	0.425	0.618
C6	SMG-R	0.069	0.143	0.139	0.052	0.111	0.100
C6	STG-R	0.428	0.356	0.210	0.369	0.416	0.224
C6	MTG-R	0.129	0.021	0.017	0.132	0.030	0.018
T4	STG-R	0.009	0.009	0.026	0.011	0.011	0.029
T4	MTG-R	0.636	0.836	0.890	0.673	0.829	0.883
T4	ITG-R	0.346	0.153	0.078	0.305	0.159	0.081
FT7	IFGoperc-L	0.009	0.010	0.006	0.013	0.010	0.006
FT7	IFGtriang-L	0.016	0.008	0.007	0.019	0.008	0.007
FT7	ORBinf-L	0.056	0.015	0.016	0.049	0.013	0.013
FT7	STG-L	0.033	0.115	0.070	0.029	0.070	0.054
FT7	TPOsup-L	0.333	0.495	0.385	0.419	0.519	0.362
FT7	MTG-L	0.185	0.136	0.209	0.129	0.117	0.214
FT7	TPOmid-L	0.322	0.202	0.295	0.294	0.246	0.331
FT7	ITG-L	0.033	0.005	0.006	0.029	0.005	0.008
F7	MFG-L	0.020	0.004	0.004	0.019	0.004	0.004
F7	ORBmid-L	0.076	0.007	0.013	0.064	0.005	0.010
F7	IFGtriang-L	0.142	0.105	0.056	0.146	0.139	0.068
F7	ORBinf-L	0.723	0.834	0.842	0.727	0.810	0.852
F7	TPOsup-L	0.029	0.045	0.075	0.033	0.037	0.058
AF7	SFGdor-L	0.019	0.003	0.004	0.016	0.002	0.004
AF7	ORBsup-L	0.014	0.004	0.008	0.012	0.003	0.008
AF7	MFG-L	0.172	0.085	0.056	0.183	0.101	0.066
AF7	ORBmid-L	0.634	0.679	0.739	0.623	0.688	0.749
AF7	IFGtriang-L	0.033	0.020	0.015	0.034	0.019	0.016
AF7	ORBinf-L	0.123	0.209	0.176	0.128	0.187	0.155
Fp1	SFGdor-L	0.260	0.172	0.074	0.277	0.209	0.096

SD distance = 10 mm		Circumferential SD pair			Vertical channel SD pair		
10/10 Points	AAL regions	0 year	1 year	2 year	0 year	1 year	2 year
Fp1	ORBsup-L	0.219	0.397	0.375	0.205	0.404	0.381
Fp1	MFG-L	0.148	0.078	0.025	0.154	0.068	0.031
Fp1	ORBmid-L	0.257	0.320	0.431	0.273	0.285	0.420
Fp1	SFGmed-L	0.054	0.016	0.020	0.042	0.016	0.017
Fp1	ORBmed-L	0.034	0.014	0.065	0.025	0.015	0.045
Fp2	SFGdor-R	0.383	0.293	0.181	0.395	0.337	0.196
Fp2	ORBsup-R	0.164	0.323	0.321	0.183	0.307	0.301
Fp2	MFG-R	0.106	0.047	0.055	0.082	0.050	0.064
Fp2	ORBmid-R	0.134	0.229	0.358	0.134	0.220	0.372
Fp2	SFGmed-R	0.102	0.034	0.019	0.097	0.030	0.016
Fp2	SFGmed-L	0.012	0.001	0.001	0.011	0.001	0.001
Fp2	ORBmed-R	0.069	0.066	0.051	0.068	0.049	0.036
AF8	SFGdor-R	0.037	0.003	0.006	0.038	0.003	0.006
AF8	ORBsup-R	0.015	0.002	0.007	0.017	0.002	0.007
AF8	MFG-R	0.288	0.084	0.087	0.332	0.130	0.105
AF8	ORBmid-R	0.463	0.782	0.677	0.448	0.730	0.683
AF8	IFGtriang-R	0.059	0.023	0.038	0.043	0.024	0.035
AF8	ORBinf-R	0.130	0.106	0.183	0.115	0.109	0.161
F8	MFG-R	0.023	0.001	0.006	0.026	0.001	0.006
F8	ORBmid-R	0.062	0.003	0.018	0.052	0.003	0.017
F8	IFGtriang-R	0.180	0.201	0.294	0.265	0.260	0.352
F8	ORBinf-R	0.693	0.733	0.578	0.620	0.693	0.536
F8	TPOsup-R	0.029	0.050	0.087	0.024	0.034	0.074
FT8	IFGoperc-R	0.014	0.004	0.012	0.023	0.004	0.014
FT8	IFGtriang-R	0.025	0.005	0.015	0.031	0.005	0.015
FT8	ORBinf-R	0.070	0.007	0.015	0.060	0.007	0.014
FT8	ROL-R	0.011	0.006	0.012	0.018	0.007	0.014
FT8	STG-R	0.075	0.140	0.136	0.100	0.181	0.143
FT8	TPOsup-R	0.279	0.154	0.258	0.284	0.147	0.269
FT8	MTG-R	0.393	0.650	0.518	0.356	0.615	0.490
FT8	TPOmid-R	0.094	0.024	0.026	0.089	0.023	0.031
FT8	ITG-R	0.030	0.009	0.006	0.029	0.010	0.006

SD distance = 10 mm		Circumferential SD pair			Vertical channel SD pair		
10/10 Points	AAL regions	0 year	1 year	2 year	0 year	1 year	2 year
TP7	STG-L	0.030	0.029	0.017	0.042	0.033	0.022
TP7	MTG-L	0.668	0.930	0.879	0.704	0.912	0.867
TP7	ITG-L	0.301	0.040	0.104	0.253	0.055	0.111
T5	STG-L	0.006	0.010	0.007	0.009	0.014	0.007
T5	MTG-L	0.828	0.935	0.922	0.801	0.936	0.907
T5	ITG-L	0.162	0.048	0.066	0.186	0.043	0.079
PO7	MOG-L	0.442	0.420	0.544	0.404	0.464	0.544
PO7	ANG-L	0.006	0.015	0.006	0.007	0.019	0.007
PO7	MTG-L	0.542	0.559	0.443	0.552	0.511	0.441
O1	SOG-L	0.184	0.149	0.266	0.105	0.114	0.266
O1	MOG-L	0.798	0.840	0.718	0.879	0.875	0.717
O2	CUN-R	0.058	0.081	0.040	0.039	0.067	0.036
O2	SOG-R	0.694	0.790	0.611	0.733	0.802	0.611
O2	MOG-R	0.230	0.121	0.343	0.211	0.122	0.347
PO8	SOG-R	0.007	0.012	0.005	0.005	0.013	0.005
PO8	MOG-R	0.953	0.946	0.899	0.958	0.953	0.904
PO8	ANG-R	0.013	0.024	0.038	0.013	0.020	0.044
PO8	MTG-R	0.019	0.012	0.055	0.013	0.008	0.043
T6	MOG-R	0.107	0.125	0.062	0.079	0.090	0.053
T6	IOG-R	0.077	0.042	0.015	0.093	0.037	0.016
T6	ANG-R	0.013	0.023	0.024	0.017	0.028	0.025
T6	STG-R	0.004	0.003	0.011	0.004	0.004	0.010
T6	MTG-R	0.673	0.779	0.864	0.678	0.815	0.869
T6	ITG-R	0.124	0.025	0.020	0.127	0.023	0.021
TP8	STG-R	0.007	0.015	0.038	0.011	0.017	0.039
TP8	MTG-R	0.758	0.934	0.931	0.754	0.915	0.919
TP8	ITG-R	0.232	0.049	0.027	0.233	0.065	0.038
FC5	PreCG-L	0.078	0.145	0.163	0.132	0.103	0.144
FC5	IFGoperc-L	0.221	0.388	0.387	0.379	0.576	0.496
FC5	IFGtriang-L	0.467	0.236	0.237	0.282	0.200	0.204
FC5	ORBinf-L	0.022	0.003	0.003	0.015	0.003	0.003

SD distance = 10 mm		Circumferential SD pair			Vertical channel SD pair		
10/10 Points	AAL regions	0 year	1 year	2 year	0 year	1 year	2 year
FC5	ROL-L	0.093	0.186	0.160	0.079	0.087	0.107
FC5	PoCG-L	0.023	0.016	0.012	0.025	0.008	0.008
FC5	STG-L	0.021	0.007	0.008	0.018	0.004	0.007
FC5	TPOsup-L	0.061	0.015	0.026	0.058	0.014	0.028
FC3	PreCG-L	0.158	0.149	0.101	0.109	0.081	0.092
FC3	MFG-L	0.562	0.656	0.716	0.552	0.710	0.669
FC3	IFGoperc-L	0.197	0.157	0.134	0.244	0.177	0.183
FC3	IFGtriang-L	0.069	0.035	0.043	0.085	0.030	0.050
FC3	PoCG-L	0.010	0.002	0.003	0.008	0.001	0.003
FC1	PreCG-L	0.049	0.002	0.001	0.012	0.001	0.001
FC1	SFGdor-L	0.372	0.428	0.488	0.390	0.480	0.522
FC1	MFG-L	0.556	0.558	0.491	0.574	0.505	0.453
FC1	SFGmed-L	0.011	0.004	0.012	0.009	0.005	0.014
FC2	PreCG-R	0.031	0.001	0.002	0.070	0.001	0.002
FC2	SFGdor-R	0.315	0.346	0.231	0.325	0.336	0.249
FC2	MFG-R	0.614	0.623	0.746	0.571	0.635	0.729
FC2	SMA-R	0.018	0.013	0.007	0.017	0.015	0.007
FC2	SFGmed-R	0.014	0.016	0.012	0.010	0.012	0.012
FC4	PreCG-R	0.219	0.165	0.090	0.158	0.131	0.084
FC4	MFG-R	0.638	0.779	0.826	0.631	0.810	0.825
FC4	IFGoperc-R	0.068	0.028	0.041	0.124	0.033	0.047
FC4	IFGtriang-R	0.049	0.022	0.039	0.058	0.021	0.040
FC4	PoCG-R	0.022	0.005	0.003	0.025	0.004	0.003
FC6	PreCG-R	0.051	0.129	0.092	0.069	0.176	0.086
FC6	MFG-R	0.018	0.005	0.011	0.019	0.007	0.011
FC6	IFGoperc-R	0.332	0.338	0.377	0.351	0.358	0.432
FC6	IFGtriang-R	0.320	0.135	0.352	0.266	0.122	0.308
FC6	ORBinf-R	0.012	0.001	0.001	0.013	0.001	0.001
FC6	ROL-R	0.077	0.096	0.053	0.079	0.114	0.060
FC6	PoCG-R	0.102	0.269	0.093	0.099	0.194	0.077
FC6	STG-R	0.045	0.018	0.012	0.052	0.020	0.015
FC6	TPOsup-R	0.026	0.006	0.007	0.033	0.007	0.009

SD distance = 10 mm		Circumferential SD pair			Vertical channel SD pair		
10/10 Points	AAL regions	0 year	1 year	2 year	0 year	1 year	2 year
FC6	MTG-R	0.012	0.002	0.001	0.014	0.002	0.001
F5	MFG-L	0.339	0.230	0.188	0.236	0.224	0.158
F5	ORBmid-L	0.020	0.003	0.006	0.019	0.003	0.005
F5	IFGtriang-L	0.570	0.746	0.762	0.649	0.745	0.792
F5	ORBinf-L	0.057	0.019	0.039	0.081	0.025	0.042
F3	SFGdor-L	0.013	0.005	0.010	0.014	0.006	0.010
F3	MFG-L	0.942	0.976	0.954	0.934	0.977	0.962
F3	IFGtriang-L	0.039	0.018	0.034	0.044	0.016	0.026
F1	SFGdor-L	0.448	0.606	0.625	0.538	0.652	0.636
F1	MFG-L	0.477	0.355	0.310	0.384	0.315	0.310
F1	SFGmed-R	0.010	0.001	0.005	0.011	0.001	0.005
F1	SFGmed-L	0.060	0.038	0.060	0.062	0.031	0.049
F2	SFGdor-R	0.707	0.801	0.694	0.670	0.837	0.713
F2	MFG-R	0.186	0.111	0.251	0.200	0.099	0.237
F2	SFGmed-R	0.090	0.085	0.051	0.107	0.061	0.047
F2	SFGmed-L	0.011	0.002	0.003	0.014	0.002	0.003
F4	SFGdor-R	0.039	0.008	0.017	0.038	0.009	0.014
F4	MFG-R	0.901	0.960	0.943	0.904	0.968	0.940
F4	IFGtriang-R	0.051	0.031	0.038	0.048	0.022	0.045
F6	MFG-R	0.288	0.128	0.136	0.274	0.097	0.132
F6	ORBmid-R	0.018	0.003	0.006	0.018	0.004	0.006
F6	IFGoperc-R	0.011	0.005	0.002	0.010	0.005	0.002
F6	IFGtriang-R	0.639	0.854	0.846	0.649	0.881	0.849
F6	ORBinf-R	0.033	0.008	0.008	0.038	0.011	0.009
AF3	SFGdor-L	0.449	0.443	0.501	0.382	0.388	0.526
AF3	ORBsup-L	0.011	0.008	0.014	0.012	0.005	0.014
AF3	MFG-L	0.461	0.524	0.438	0.533	0.587	0.417
AF3	ORBmid-L	0.025	0.015	0.020	0.029	0.011	0.020
AF3	SFGmed-L	0.037	0.008	0.023	0.029	0.007	0.020
AF4	SFGdor-R	0.446	0.496	0.326	0.523	0.605	0.329
AF4	MFG-R	0.448	0.472	0.638	0.376	0.360	0.632
AF4	ORBmid-R	0.017	0.005	0.012	0.018	0.005	0.014

SD distance = 10 mm		Circumferential SD pair			Vertical channel SD pair		
10/10 Points	AAL regions	0 year	1 year	2 year	0 year	1 year	2 year
AF4	SFGmed-R	0.060	0.022	0.015	0.054	0.025	0.014
CP5	PoCG-L	0.015	0.002	0.002	0.010	0.002	0.002
CP5	SMG-L	0.210	0.509	0.446	0.304	0.509	0.445
CP5	STG-L	0.724	0.465	0.523	0.614	0.465	0.523
CP5	MTG-L	0.046	0.013	0.019	0.068	0.016	0.021
CP3	PoCG-L	0.057	0.064	0.059	0.065	0.054	0.049
CP3	SPG-L	0.008	0.008	0.013	0.008	0.012	0.012
CP3	IPL-L	0.430	0.750	0.679	0.360	0.742	0.688
CP3	SMG-L	0.498	0.176	0.241	0.560	0.189	0.243
CP1	PreCG-L	0.087	0.046	0.067	0.099	0.052	0.070
CP1	PoCG-L	0.681	0.711	0.691	0.580	0.703	0.683
CP1	SPG-L	0.196	0.224	0.219	0.275	0.225	0.222
CP1	IPL-L	0.025	0.016	0.018	0.034	0.016	0.020
CP2	PreCG-R	0.082	0.023	0.045	0.072	0.026	0.048
CP2	PoCG-R	0.601	0.575	0.647	0.576	0.583	0.647
CP2	SPG-R	0.248	0.351	0.261	0.281	0.342	0.256
CP2	IPL-R	0.014	0.011	0.017	0.018	0.015	0.019
CP2	PCUN-R	0.023	0.024	0.012	0.019	0.018	0.011
CP2	PCL-R	0.019	0.014	0.014	0.021	0.014	0.015
CP4	PoCG-R	0.119	0.075	0.151	0.143	0.091	0.171
CP4	IPL-R	0.821	0.904	0.825	0.801	0.889	0.804
CP4	SMG-R	0.034	0.006	0.010	0.031	0.005	0.011
CP4	ANG-R	0.013	0.008	0.004	0.012	0.008	0.004
CP6	PoCG-R	0.012	0.003	0.007	0.011	0.002	0.007
CP6	IPL-R	0.029	0.048	0.050	0.044	0.061	0.049
CP6	SMG-R	0.488	0.644	0.770	0.528	0.610	0.739
CP6	ANG-R	0.022	0.016	0.011	0.014	0.016	0.009
CP6	STG-R	0.337	0.265	0.149	0.313	0.283	0.182
CP6	MTG-R	0.111	0.023	0.013	0.089	0.027	0.015
P5	MOG-L	0.039	0.008	0.014	0.014	0.008	0.013
P5	IPL-L	0.007	0.031	0.032	0.010	0.039	0.034
P5	SMG-L	0.034	0.017	0.013	0.029	0.011	0.012

SD distance = 10 mm		Circumferential SD pair			Vertical channel SD pair		
10/10 Points	AAL regions	0 year	1 year	2 year	0 year	1 year	2 year
P5	ANG-L	0.456	0.789	0.801	0.437	0.757	0.751
P5	STG-L	0.196	0.067	0.033	0.145	0.060	0.030
P5	MTG-L	0.268	0.089	0.107	0.365	0.125	0.160
P3	SPG-L	0.026	0.030	0.025	0.024	0.028	0.027
P3	IPL-L	0.371	0.451	0.281	0.338	0.307	0.279
P3	ANG-L	0.591	0.516	0.689	0.626	0.662	0.691
P1	SPG-L	0.815	0.915	0.900	0.807	0.919	0.892
P1	IPL-L	0.068	0.029	0.034	0.055	0.023	0.033
P1	ANG-L	0.093	0.045	0.051	0.116	0.048	0.060
P2	SPG-R	0.680	0.740	0.830	0.727	0.739	0.837
P2	IPL-R	0.022	0.007	0.016	0.023	0.008	0.022
P2	ANG-R	0.017	0.004	0.010	0.016	0.004	0.013
P2	PCUN-R	0.245	0.242	0.134	0.200	0.241	0.119
P4	MOG-R	0.019	0.005	0.006	0.035	0.006	0.006
P4	SPG-R	0.066	0.117	0.108	0.075	0.125	0.107
P4	IPL-R	0.182	0.123	0.244	0.121	0.108	0.225
P4	ANG-R	0.725	0.753	0.638	0.763	0.759	0.658
P6	MOG-R	0.068	0.030	0.013	0.045	0.030	0.011
P6	IPL-R	0.015	0.022	0.043	0.022	0.027	0.060
P6	SMG-R	0.044	0.011	0.039	0.017	0.008	0.033
P6	ANG-R	0.767	0.901	0.873	0.834	0.908	0.867
P6	MTG-R	0.097	0.035	0.030	0.079	0.026	0.027
PO3	SOG-L	0.141	0.217	0.196	0.113	0.161	0.160
PO3	MOG-L	0.372	0.203	0.235	0.492	0.238	0.283
PO3	SPG-L	0.043	0.056	0.045	0.053	0.058	0.044
PO3	ANG-L	0.436	0.518	0.517	0.334	0.538	0.507
PO4	CUN-R	0.015	0.010	0.004	0.016	0.009	0.004
PO4	SOG-R	0.457	0.478	0.250	0.507	0.492	0.253
PO4	MOG-R	0.317	0.249	0.328	0.234	0.242	0.311
PO4	SPG-R	0.029	0.049	0.053	0.053	0.067	0.069
PO4	IPL-R	0.155	0.198	0.336	0.158	0.176	0.339

SD distance = 10 mm		Circumferential SD pair			Vertical channel SD pair		
10/10 Points	AAL regions	0 year	1 year	2 year	0 year	1 year	2 year
PO4	ANG-R	0.013	0.008	0.023	0.014	0.007	0.016

Table S4 Normalized PPL of corresponding brain regions ($L_{norm,M}$) for the circumferential and vertical SD pairs set at 10/10 fiducial points at 15-mm SD distance from 0-yo, 1-yo, and 2-yo.

SD distance = 15 mm		Circumferential SD pair			Vertical SD pair		
10/10 Point	AAL regions	0 year	1 year	2 year	0 year	1 year	2 year
Cz	PreCG-R	0.021	0.005	0.005	0.014	0.003	0.004
Cz	PreCG-L	0.060	0.019	0.011	0.078	0.024	0.015
Cz	SFGdor-R	0.110	0.040	0.050	0.051	0.021	0.033
Cz	SFGdor-L	0.208	0.151	0.099	0.151	0.115	0.070
Cz	SMA-R	0.219	0.415	0.415	0.172	0.290	0.427
Cz	SMA-L	0.191	0.322	0.385	0.307	0.470	0.389
Cz	PoCG-L	0.044	0.003	0.002	0.051	0.004	0.003
Cz	SPG-L	0.039	0.000	0.000	0.025	0.000	0.000
Cz	PCL-R	0.061	0.033	0.023	0.077	0.051	0.041
Cz	PCL-L	0.020	0.008	0.006	0.046	0.017	0.013
Fpz	SFGdor-R	0.052	0.023	0.027	0.029	0.013	0.025
Fpz	SFGdor-L	0.084	0.072	0.014	0.066	0.051	0.013
Fpz	ORBsup-R	0.038	0.029	0.062	0.024	0.020	0.050
Fpz	ORBsup-L	0.085	0.138	0.076	0.075	0.110	0.058
Fpz	ORBmid-L	0.019	0.011	0.009	0.017	0.010	0.007
Fpz	SFGmed-R	0.207	0.117	0.078	0.196	0.103	0.111
Fpz	SFGmed-L	0.207	0.186	0.080	0.276	0.225	0.101
Fpz	ORBmed-R	0.137	0.190	0.310	0.126	0.161	0.326
Fpz	ORBmed-L	0.134	0.217	0.300	0.158	0.283	0.258
Fpz	REC-R	0.010	0.011	0.028	0.011	0.014	0.035
AFz	SFGdor-R	0.087	0.027	0.082	0.045	0.018	0.051
AFz	SFGdor-L	0.185	0.159	0.058	0.075	0.094	0.037
AFz	MFG-L	0.019	0.009	0.004	0.013	0.007	0.003
AFz	SFGmed-R	0.359	0.385	0.493	0.390	0.332	0.557
AFz	SFGmed-L	0.331	0.413	0.354	0.457	0.540	0.342
Fz	SFGdor-R	0.083	0.025	0.085	0.059	0.020	0.059
Fz	SFGdor-L	0.198	0.250	0.062	0.093	0.089	0.041
Fz	MFG-L	0.012	0.003	0.003	0.013	0.004	0.003
Fz	SMA-R	0.091	0.001	0.000	0.016	0.001	0.000
Fz	SMA-L	0.030	0.001	0.000	0.014	0.001	0.000

SD distance = 15 mm		Circumferential SD pair			Vertical SD pair		
10/10 Point	AAL regions	0 year	1 year	2 year	0 year	1 year	2 year
Fz	SFGmed-R	0.275	0.292	0.486	0.394	0.330	0.553
Fz	SFGmed-L	0.304	0.426	0.359	0.403	0.553	0.341
FCz	SFGdor-R	0.103	0.030	0.064	0.060	0.015	0.052
FCz	SFGdor-L	0.194	0.068	0.047	0.109	0.063	0.030
FCz	SMA-R	0.241	0.317	0.178	0.227	0.151	0.234
FCz	SMA-L	0.229	0.238	0.112	0.267	0.347	0.124
FCz	SFGmed-R	0.080	0.145	0.307	0.120	0.122	0.327
FCz	SFGmed-L	0.112	0.198	0.284	0.186	0.298	0.227
FCz	PCL-R	0.012	0.000	0.000	0.005	0.000	0.000
CPz	PreCG-R	0.014	0.002	0.003	0.007	0.002	0.003
CPz	PreCG-L	0.051	0.017	0.020	0.060	0.021	0.019
CPz	SMA-R	0.011	0.004	0.005	0.011	0.005	0.008
CPz	SMA-L	0.015	0.008	0.010	0.020	0.014	0.017
CPz	PoCG-R	0.058	0.014	0.022	0.020	0.007	0.016
CPz	PoCG-L	0.266	0.270	0.191	0.226	0.201	0.113
CPz	SPG-R	0.018	0.010	0.010	0.010	0.006	0.008
CPz	SPG-L	0.056	0.044	0.025	0.094	0.049	0.024
CPz	PCUN-R	0.180	0.192	0.200	0.111	0.143	0.206
CPz	PCUN-L	0.054	0.116	0.078	0.123	0.187	0.107
CPz	PCL-R	0.160	0.167	0.254	0.102	0.155	0.282
CPz	PCL-L	0.097	0.154	0.179	0.198	0.208	0.195
Pz	PoCG-L	0.014	0.006	0.005	0.024	0.006	0.005
Pz	SPG-R	0.042	0.016	0.021	0.017	0.009	0.014
Pz	SPG-L	0.410	0.361	0.217	0.403	0.332	0.161
Pz	PCUN-R	0.329	0.317	0.445	0.227	0.253	0.444
Pz	PCUN-L	0.181	0.294	0.305	0.302	0.391	0.370
POz	CUN-R	0.138	0.079	0.094	0.144	0.094	0.117
POz	CUN-L	0.156	0.236	0.135	0.180	0.258	0.139
POz	SOG-R	0.152	0.081	0.116	0.065	0.059	0.082
POz	SOG-L	0.072	0.047	0.015	0.065	0.034	0.012
POz	MOG-L	0.013	0.003	0.001	0.013	0.002	0.001
POz	SPG-R	0.020	0.024	0.038	0.010	0.016	0.023

SD distance = 15 mm		Circumferential SD pair			Vertical SD pair		
10/10 Point	AAL regions	0 year	1 year	2 year	0 year	1 year	2 year
POz	SPG-L	0.224	0.279	0.237	0.255	0.243	0.192
POz	PCUN-R	0.142	0.165	0.256	0.138	0.173	0.290
POz	PCUN-L	0.065	0.084	0.104	0.115	0.119	0.143
Oz	CAL-R	0.034	0.021	0.030	0.054	0.035	0.047
Oz	CUN-R	0.328	0.297	0.499	0.274	0.320	0.546
Oz	CUN-L	0.172	0.203	0.201	0.269	0.291	0.221
Oz	SOG-R	0.052	0.047	0.070	0.029	0.033	0.050
Oz	SOG-L	0.357	0.393	0.177	0.317	0.289	0.115
Oz	MOG-L	0.038	0.027	0.008	0.029	0.017	0.005
T3	STG-L	0.031	0.063	0.042	0.050	0.131	0.063
T3	TPOsup-L	0.016	0.006	0.005	0.016	0.007	0.005
T3	MTG-L	0.647	0.882	0.874	0.640	0.809	0.836
T3	TPOmid-L	0.037	0.007	0.008	0.026	0.006	0.006
T3	ITG-L	0.264	0.042	0.069	0.264	0.045	0.089
C5	PreCG-L	0.024	0.023	0.020	0.019	0.013	0.014
C5	ROL-L	0.098	0.129	0.074	0.066	0.054	0.040
C5	PoCG-L	0.203	0.435	0.460	0.318	0.478	0.483
C5	SMG-L	0.057	0.093	0.116	0.038	0.088	0.094
C5	STG-L	0.549	0.311	0.316	0.492	0.359	0.356
C5	TPOsup-L	0.020	0.003	0.003	0.014	0.002	0.002
C5	MTG-L	0.032	0.004	0.006	0.042	0.005	0.008
C3	PreCG-L	0.329	0.432	0.320	0.175	0.310	0.308
C3	MFG-L	0.026	0.040	0.049	0.118	0.088	0.074
C3	PoCG-L	0.451	0.477	0.524	0.622	0.571	0.550
C3	SMG-L	0.181	0.048	0.101	0.077	0.029	0.064
C1	PreCG-L	0.493	0.506	0.445	0.421	0.481	0.457
C1	SFGdor-L	0.199	0.251	0.268	0.330	0.387	0.333
C1	MFG-L	0.235	0.204	0.254	0.164	0.089	0.168
C1	PoCG-L	0.059	0.034	0.028	0.066	0.031	0.035
C2	PreCG-R	0.466	0.660	0.484	0.480	0.619	0.476
C2	SFGdor-R	0.240	0.142	0.246	0.328	0.255	0.322
C2	MFG-R	0.188	0.129	0.221	0.069	0.055	0.152

SD distance = 15 mm		Circumferential SD pair			Vertical SD pair		
10/10 Point	AAL regions	0 year	1 year	2 year	0 year	1 year	2 year
C2	SMA-R	0.015	0.006	0.010	0.015	0.008	0.015
C2	PoCG-R	0.071	0.058	0.033	0.088	0.058	0.029
C4	PreCG-R	0.209	0.190	0.306	0.135	0.128	0.319
C4	MFG-R	0.018	0.005	0.017	0.010	0.005	0.017
C4	PoCG-R	0.458	0.662	0.591	0.531	0.768	0.603
C4	IPL-R	0.130	0.112	0.050	0.081	0.049	0.025
C4	SMG-R	0.179	0.031	0.035	0.238	0.050	0.034
C6	PreCG-R	0.009	0.004	0.021	0.015	0.003	0.016
C6	IFGoperc-R	0.011	0.001	0.006	0.009	0.000	0.004
C6	ROL-R	0.059	0.036	0.027	0.036	0.013	0.023
C6	PoCG-R	0.242	0.430	0.609	0.391	0.401	0.551
C6	SMG-R	0.065	0.127	0.132	0.057	0.102	0.088
C6	STG-R	0.425	0.371	0.186	0.305	0.423	0.286
C6	MTG-R	0.169	0.029	0.017	0.168	0.057	0.029
T4	STG-R	0.013	0.011	0.027	0.018	0.016	0.034
T4	MTG-R	0.651	0.818	0.869	0.662	0.792	0.854
T4	ITG-R	0.318	0.168	0.097	0.305	0.190	0.104
FT7	IFGoperc-L	0.012	0.011	0.007	0.021	0.011	0.010
FT7	IFGtriang-L	0.025	0.011	0.009	0.032	0.008	0.011
FT7	ORBinf-L	0.089	0.024	0.022	0.071	0.013	0.019
FT7	STG-L	0.039	0.135	0.078	0.031	0.088	0.064
FT7	TPOsup-L	0.350	0.424	0.355	0.421	0.460	0.419
FT7	MTG-L	0.174	0.180	0.250	0.111	0.156	0.190
FT7	TPOmid-L	0.263	0.195	0.266	0.256	0.241	0.273
FT7	ITG-L	0.029	0.007	0.008	0.033	0.008	0.008
F7	MFG-L	0.021	0.006	0.005	0.023	0.005	0.004
F7	ORBmid-L	0.100	0.011	0.019	0.064	0.007	0.010
F7	IFGtriang-L	0.132	0.117	0.077	0.199	0.241	0.103
F7	ORBinf-L	0.680	0.796	0.811	0.657	0.689	0.808
F7	TPOsup-L	0.050	0.061	0.077	0.042	0.049	0.066
AF7	SFGdor-L	0.021	0.004	0.005	0.023	0.004	0.005
AF7	ORBsup-L	0.016	0.005	0.010	0.015	0.005	0.010

SD distance = 15 mm		Circumferential SD pair			Vertical SD pair		
10/10 Point	AAL regions	0 year	1 year	2 year	0 year	1 year	2 year
AF7	MFG-L	0.153	0.093	0.063	0.259	0.161	0.098
AF7	ORBmid-L	0.562	0.603	0.643	0.534	0.639	0.712
AF7	IFGtriang-L	0.055	0.028	0.023	0.044	0.027	0.021
AF7	ORBinf-L	0.187	0.266	0.253	0.119	0.163	0.152
Fp1	SFGdor-L	0.245	0.157	0.066	0.285	0.224	0.131
Fp1	ORBsup-L	0.203	0.392	0.361	0.216	0.336	0.373
Fp1	MFG-L	0.143	0.069	0.024	0.124	0.082	0.036
Fp1	ORBmid-L	0.253	0.325	0.405	0.255	0.321	0.372
Fp1	SFGmed-R	0.013	0.001	0.002	0.010	0.001	0.002
Fp1	SFGmed-L	0.073	0.023	0.025	0.056	0.017	0.023
Fp1	ORBmed-L	0.046	0.028	0.103	0.035	0.017	0.053
Fp2	SFGdor-R	0.333	0.283	0.183	0.378	0.367	0.224
Fp2	ORBsup-R	0.147	0.274	0.294	0.173	0.269	0.272
Fp2	MFG-R	0.131	0.062	0.068	0.093	0.058	0.088
Fp2	ORBmid-R	0.156	0.229	0.351	0.140	0.197	0.348
Fp2	ORBinf-R	0.011	0.001	0.005	0.008	0.001	0.005
Fp2	SFGmed-R	0.109	0.050	0.023	0.101	0.041	0.017
Fp2	SFGmed-L	0.014	0.002	0.002	0.013	0.002	0.001
Fp2	ORBmed-R	0.071	0.092	0.062	0.066	0.058	0.035
Fp2	ORBmed-L	0.011	0.002	0.004	0.010	0.002	0.003
AF8	SFGdor-R	0.054	0.007	0.009	0.042	0.004	0.008
AF8	ORBsup-R	0.023	0.004	0.010	0.018	0.003	0.008
AF8	MFG-R	0.261	0.152	0.093	0.349	0.173	0.166
AF8	ORBmid-R	0.436	0.667	0.640	0.406	0.628	0.625
AF8	IFGtriang-R	0.069	0.051	0.043	0.054	0.040	0.047
AF8	ORBinf-R	0.147	0.118	0.201	0.123	0.151	0.143
F8	MFG-R	0.034	0.002	0.007	0.027	0.002	0.008
F8	ORBmid-R	0.084	0.004	0.022	0.048	0.004	0.019
F8	IFGtriang-R	0.242	0.167	0.313	0.286	0.288	0.380
F8	ORBinf-R	0.581	0.742	0.531	0.584	0.641	0.495
F8	TPOsup-R	0.037	0.069	0.105	0.035	0.051	0.080
FT8	IFGoperc-R	0.021	0.004	0.014	0.032	0.006	0.017

SD distance = 15 mm		Circumferential SD pair			Vertical SD pair		
10/10 Point	AAL regions	0 year	1 year	2 year	0 year	1 year	2 year
FT8	IFGtriang-R	0.044	0.006	0.019	0.045	0.007	0.019
FT8	ORBinf-R	0.112	0.010	0.021	0.077	0.009	0.017
FT8	ROL-R	0.016	0.006	0.014	0.023	0.009	0.017
FT8	STG-R	0.093	0.110	0.130	0.094	0.183	0.134
FT8	TPOsup-R	0.256	0.175	0.258	0.296	0.157	0.263
FT8	MTG-R	0.355	0.640	0.501	0.286	0.580	0.480
FT8	TPOmid-R	0.064	0.033	0.032	0.102	0.032	0.041
FT8	ITG-R	0.026	0.013	0.007	0.031	0.015	0.009
TP7	STG-L	0.039	0.037	0.022	0.079	0.081	0.028
TP7	MTG-L	0.635	0.907	0.852	0.640	0.818	0.785
TP7	ITG-L	0.324	0.056	0.125	0.279	0.100	0.187
T5	STG-L	0.010	0.018	0.007	0.018	0.022	0.010
T5	MTG-L	0.810	0.925	0.892	0.747	0.897	0.857
T5	ITG-L	0.172	0.047	0.092	0.229	0.068	0.124
PO7	MOG-L	0.455	0.471	0.520	0.377	0.504	0.573
PO7	IOG-L	0.011	0.003	0.005	0.039	0.005	0.008
PO7	ANG-L	0.007	0.022	0.009	0.014	0.032	0.012
PO7	MTG-L	0.513	0.500	0.463	0.531	0.454	0.403
PO7	ITG-L	0.012	0.003	0.002	0.037	0.004	0.003
O1	SOG-L	0.220	0.191	0.274	0.163	0.116	0.231
O1	MOG-L	0.752	0.793	0.705	0.806	0.868	0.747
O2	CAL-R	0.014	0.006	0.002	0.015	0.007	0.002
O2	CUN-R	0.084	0.107	0.048	0.044	0.087	0.036
O2	SOG-R	0.597	0.720	0.536	0.622	0.754	0.564
O2	MOG-R	0.291	0.161	0.408	0.306	0.144	0.392
PO8	SOG-R	0.014	0.018	0.006	0.006	0.012	0.006
PO8	MOG-R	0.924	0.921	0.872	0.943	0.921	0.892
PO8	IOG-R	0.010	0.006	0.004	0.012	0.006	0.003
PO8	ANG-R	0.019	0.034	0.043	0.020	0.042	0.053
PO8	MTG-R	0.030	0.019	0.074	0.016	0.015	0.045
T6	MOG-R	0.126	0.174	0.072	0.097	0.136	0.066
T6	IOG-R	0.064	0.049	0.013	0.134	0.071	0.023

SD distance = 15 mm		Circumferential SD pair			Vertical SD pair		
10/10 Point	AAL regions	0 year	1 year	2 year	0 year	1 year	2 year
T6	ANG-R	0.019	0.028	0.033	0.031	0.041	0.035
T6	STG-R	0.008	0.004	0.015	0.006	0.004	0.013
T6	MTG-R	0.666	0.714	0.842	0.593	0.713	0.831
T6	ITG-R	0.113	0.028	0.017	0.134	0.030	0.023
TP8	STG-R	0.012	0.020	0.043	0.015	0.025	0.055
TP8	MTG-R	0.751	0.913	0.918	0.686	0.876	0.894
TP8	ITG-R	0.232	0.064	0.036	0.295	0.096	0.047
FC5	PreCG-L	0.087	0.170	0.142	0.126	0.139	0.122
FC5	MFG-L	0.011	0.004	0.004	0.010	0.005	0.004
FC5	IFGoperc-L	0.170	0.308	0.305	0.309	0.498	0.484
FC5	IFGtriang-L	0.441	0.282	0.276	0.322	0.180	0.229
FC5	ORBinf-L	0.026	0.004	0.004	0.026	0.005	0.004
FC5	ROL-L	0.125	0.177	0.204	0.070	0.122	0.100
FC5	PoCG-L	0.041	0.028	0.020	0.027	0.015	0.011
FC5	STG-L	0.029	0.009	0.012	0.021	0.008	0.008
FC5	TPOsup-L	0.060	0.018	0.031	0.079	0.027	0.036
FC3	PreCG-L	0.229	0.207	0.130	0.113	0.104	0.081
FC3	MFG-L	0.511	0.618	0.685	0.566	0.659	0.637
FC3	IFGoperc-L	0.162	0.130	0.129	0.199	0.191	0.201
FC3	IFGtriang-L	0.076	0.041	0.048	0.107	0.042	0.075
FC3	PoCG-L	0.017	0.003	0.005	0.010	0.002	0.003
FC1	PreCG-L	0.078	0.002	0.002	0.038	0.002	0.001
FC1	SFGdor-L	0.324	0.434	0.483	0.395	0.455	0.484
FC1	MFG-L	0.568	0.546	0.486	0.528	0.523	0.485
FC1	SFGmed-L	0.014	0.008	0.017	0.013	0.006	0.016
FC2	PreCG-R	0.029	0.002	0.003	0.027	0.002	0.003
FC2	SFGdor-R	0.384	0.383	0.265	0.439	0.361	0.248
FC2	MFG-R	0.519	0.574	0.705	0.474	0.600	0.725
FC2	SMA-R	0.029	0.017	0.008	0.031	0.022	0.009
FC2	SFGmed-R	0.025	0.022	0.016	0.015	0.014	0.013
FC4	PreCG-R	0.238	0.186	0.112	0.129	0.117	0.071
FC4	MFG-R	0.621	0.759	0.811	0.613	0.808	0.815

SD distance = 15 mm		Circumferential SD pair			Vertical SD pair		
10/10 Point	AAL regions	0 year	1 year	2 year	0 year	1 year	2 year
FC4	IFGoperc-R	0.057	0.023	0.034	0.148	0.040	0.051
FC4	IFGtriang-R	0.053	0.025	0.037	0.084	0.031	0.058
FC4	PoCG-R	0.025	0.006	0.004	0.020	0.004	0.003
FC6	PreCG-R	0.058	0.103	0.092	0.090	0.203	0.092
FC6	MFG-R	0.025	0.006	0.013	0.031	0.008	0.015
FC6	IFGoperc-R	0.241	0.280	0.310	0.294	0.275	0.391
FC6	IFGtriang-R	0.360	0.203	0.382	0.300	0.110	0.333
FC6	ORBinf-R	0.014	0.001	0.001	0.016	0.001	0.001
FC6	ROL-R	0.074	0.096	0.062	0.067	0.146	0.063
FC6	PoCG-R	0.135	0.274	0.112	0.095	0.204	0.072
FC6	STG-R	0.048	0.025	0.017	0.051	0.036	0.018
FC6	TPOsup-R	0.026	0.008	0.009	0.037	0.013	0.013
FC6	MTG-R	0.013	0.003	0.002	0.014	0.003	0.002
F5	MFG-L	0.291	0.258	0.238	0.269	0.241	0.193
F5	ORBmid-L	0.027	0.004	0.006	0.027	0.004	0.007
F5	IFGoperc-L	0.011	0.005	0.004	0.008	0.003	0.003
F5	IFGtriang-L	0.586	0.707	0.713	0.574	0.713	0.732
F5	ORBinf-L	0.073	0.024	0.037	0.113	0.038	0.063
F3	SFGdor-L	0.026	0.008	0.011	0.021	0.009	0.012
F3	MFG-L	0.907	0.967	0.940	0.917	0.967	0.950
F3	IFGtriang-L	0.055	0.024	0.046	0.050	0.023	0.035
F1	SFGdor-L	0.401	0.517	0.542	0.476	0.649	0.598
F1	MFG-L	0.468	0.419	0.352	0.428	0.313	0.326
F1	SFGmed-R	0.019	0.002	0.009	0.015	0.002	0.007
F1	SFGmed-L	0.105	0.060	0.096	0.074	0.036	0.067
F2	SFGdor-R	0.594	0.704	0.633	0.590	0.768	0.661
F2	MFG-R	0.233	0.173	0.291	0.261	0.138	0.282
F2	SFGmed-R	0.146	0.119	0.071	0.119	0.090	0.053
F2	SFGmed-L	0.018	0.003	0.004	0.017	0.004	0.003
F4	SFGdor-R	0.050	0.023	0.021	0.049	0.016	0.020
F4	MFG-R	0.862	0.942	0.920	0.875	0.948	0.931
F4	IFGtriang-R	0.073	0.033	0.056	0.063	0.035	0.047

SD distance = 15 mm		Circumferential SD pair			Vertical SD pair		
10/10 Point	AAL regions	0 year	1 year	2 year	0 year	1 year	2 year
F6	MFG-R	0.306	0.153	0.148	0.318	0.117	0.176
F6	ORBmid-R	0.022	0.005	0.010	0.030	0.007	0.008
F6	IFGoperc-R	0.016	0.008	0.003	0.011	0.006	0.002
F6	IFGtriang-R	0.603	0.819	0.825	0.569	0.843	0.799
F6	ORBinf-R	0.037	0.012	0.011	0.056	0.024	0.012
AF3	SFGdor-L	0.374	0.441	0.481	0.364	0.333	0.471
AF3	ORBsup-L	0.012	0.008	0.013	0.019	0.009	0.021
AF3	MFG-L	0.508	0.522	0.449	0.515	0.629	0.450
AF3	ORBmid-L	0.026	0.012	0.019	0.040	0.017	0.028
AF3	SFGmed-L	0.056	0.014	0.033	0.040	0.009	0.025
AF4	SFGdor-R	0.429	0.502	0.349	0.470	0.570	0.320
AF4	MFG-R	0.427	0.450	0.606	0.419	0.384	0.632
AF4	ORBmid-R	0.018	0.005	0.014	0.024	0.008	0.019
AF4	SFGmed-R	0.087	0.037	0.019	0.053	0.030	0.017
CP5	PoCG-L	0.019	0.005	0.004	0.014	0.004	0.003
CP5	SMG-L	0.232	0.523	0.476	0.350	0.482	0.456
CP5	ANG-L	0.007	0.017	0.015	0.003	0.007	0.009
CP5	STG-L	0.677	0.435	0.479	0.523	0.477	0.496
CP5	MTG-L	0.064	0.017	0.023	0.108	0.028	0.033
CP3	PoCG-L	0.084	0.112	0.070	0.093	0.089	0.074
CP3	SPG-L	0.011	0.014	0.015	0.010	0.013	0.015
CP3	IPL-L	0.428	0.619	0.623	0.330	0.599	0.620
CP3	SMG-L	0.468	0.251	0.282	0.555	0.295	0.279
CP1	PreCG-L	0.119	0.071	0.068	0.146	0.078	0.086
CP1	PoCG-L	0.569	0.669	0.636	0.486	0.660	0.631
CP1	SPG-L	0.264	0.237	0.267	0.300	0.237	0.249
CP1	IPL-L	0.030	0.017	0.022	0.051	0.020	0.027
CP2	PreCG-R	0.097	0.030	0.050	0.086	0.035	0.056
CP2	PoCG-R	0.513	0.539	0.609	0.472	0.556	0.614
CP2	SPG-R	0.296	0.362	0.284	0.342	0.341	0.269
CP2	IPL-R	0.020	0.014	0.023	0.028	0.019	0.023
CP2	PCUN-R	0.034	0.036	0.015	0.025	0.023	0.013

SD distance = 15 mm		Circumferential SD pair			Vertical SD pair		
10/10 Point	AAL regions	0 year	1 year	2 year	0 year	1 year	2 year
CP2	PCL-R	0.021	0.017	0.015	0.028	0.022	0.019
CP4	PoCG-R	0.148	0.096	0.190	0.151	0.152	0.202
CP4	SPG-R	0.015	0.008	0.008	0.009	0.010	0.008
CP4	IPL-R	0.771	0.875	0.780	0.765	0.821	0.767
CP4	SMG-R	0.036	0.009	0.012	0.047	0.007	0.013
CP4	ANG-R	0.021	0.011	0.005	0.021	0.009	0.005
CP6	PoCG-R	0.018	0.003	0.010	0.012	0.003	0.009
CP6	IPL-R	0.045	0.055	0.060	0.052	0.073	0.063
CP6	SMG-R	0.513	0.617	0.760	0.461	0.543	0.691
CP6	ANG-R	0.036	0.029	0.015	0.015	0.016	0.010
CP6	STG-R	0.273	0.265	0.140	0.308	0.326	0.206
CP6	MTG-R	0.113	0.030	0.015	0.150	0.039	0.020
P5	MOG-L	0.033	0.014	0.018	0.014	0.013	0.019
P5	IPL-L	0.008	0.036	0.035	0.025	0.063	0.055
P5	SMG-L	0.049	0.028	0.023	0.040	0.014	0.014
P5	ANG-L	0.330	0.726	0.730	0.432	0.662	0.690
P5	STG-L	0.257	0.085	0.057	0.114	0.051	0.027
P5	MTG-L	0.323	0.112	0.135	0.373	0.197	0.195
P3	SPG-L	0.032	0.045	0.035	0.032	0.040	0.035
P3	IPL-L	0.440	0.440	0.338	0.320	0.331	0.289
P3	SMG-L	0.013	0.002	0.002	0.008	0.002	0.001
P3	ANG-L	0.506	0.511	0.622	0.625	0.622	0.671
P1	PoCG-L	0.012	0.006	0.005	0.014	0.006	0.005
P1	SPG-L	0.783	0.902	0.881	0.761	0.883	0.877
P1	IPL-L	0.081	0.035	0.043	0.053	0.033	0.034
P1	ANG-L	0.098	0.048	0.055	0.150	0.072	0.071
P1	PCUN-L	0.015	0.006	0.011	0.009	0.004	0.009
P2	PoCG-R	0.014	0.001	0.006	0.014	0.001	0.005
P2	SPG-R	0.631	0.694	0.784	0.661	0.744	0.816
P2	IPL-R	0.029	0.010	0.020	0.039	0.010	0.025
P2	ANG-R	0.025	0.006	0.013	0.025	0.005	0.015
P2	PCUN-R	0.265	0.280	0.170	0.216	0.231	0.133

SD distance = 15 mm		Circumferential SD pair			Vertical SD pair		
10/10 Point	AAL regions	0 year	1 year	2 year	0 year	1 year	2 year
P2	PCUN-L	0.012	0.005	0.004	0.009	0.004	0.003
P4	MOG-R	0.026	0.005	0.005	0.051	0.008	0.008
P4	SPG-R	0.088	0.152	0.135	0.097	0.170	0.129
P4	IPL-R	0.263	0.176	0.280	0.146	0.129	0.249
P4	ANG-R	0.608	0.664	0.574	0.696	0.689	0.609
P6	MOG-R	0.091	0.047	0.019	0.063	0.036	0.015
P6	IPL-R	0.024	0.035	0.056	0.046	0.036	0.059
P6	SMG-R	0.073	0.024	0.054	0.025	0.014	0.035
P6	ANG-R	0.702	0.847	0.835	0.760	0.863	0.846
P6	MTG-R	0.099	0.044	0.033	0.100	0.049	0.042
PO3	SOG-L	0.143	0.234	0.199	0.118	0.175	0.153
PO3	MOG-L	0.334	0.186	0.236	0.464	0.274	0.308
PO3	SPG-L	0.046	0.064	0.051	0.088	0.087	0.057
PO3	ANG-L	0.462	0.507	0.504	0.317	0.457	0.474
PO4	CUN-R	0.023	0.013	0.006	0.021	0.012	0.006
PO4	SOG-R	0.374	0.464	0.287	0.468	0.470	0.254
PO4	MOG-R	0.375	0.270	0.305	0.244	0.246	0.327
PO4	SPG-R	0.034	0.049	0.061	0.075	0.091	0.080
PO4	IPL-R	0.152	0.183	0.307	0.151	0.163	0.305
PO4	ANG-R	0.020	0.011	0.027	0.017	0.009	0.020
PO4	PCUN-R	0.013	0.007	0.006	0.016	0.007	0.007

Table S5 Normalized PPL of corresponding brain regions ($L_{norm,M}$) for the circumferential and vertical SD pairs set at 10/10 fiducial points at 20-mm SD distance from 0-yo, 1-yo, and 2-yo.

SD distance = 20 mm		Circumferential SD pair			Vertical SD pair		
10/10 Points	AAL regions	0 year	1 year	2 year	0 year	1 year	2 year
Cz	PreCG-R	0.027	0.008	0.007	0.015	0.004	0.006
Cz	PreCG-L	0.059	0.024	0.015	0.079	0.031	0.017
Cz	SFGdor-R	0.187	0.065	0.076	0.056	0.024	0.038
Cz	SFGdor-L	0.212	0.183	0.134	0.137	0.111	0.072
Cz	SMA-R	0.168	0.371	0.392	0.173	0.278	0.418
Cz	SMA-L	0.149	0.291	0.333	0.268	0.442	0.369
Cz	PoCG-R	0.011	0.002	0.001	0.009	0.001	0.001
Cz	PoCG-L	0.033	0.003	0.002	0.048	0.006	0.003
Cz	SPG-L	0.059	0.000	0.000	0.019	0.000	0.000
Cz	PCL-R	0.060	0.039	0.026	0.093	0.069	0.054
Cz	PCL-L	0.018	0.009	0.007	0.078	0.028	0.016
Fpz	SFGdor-R	0.060	0.034	0.034	0.031	0.015	0.024
Fpz	SFGdor-L	0.103	0.070	0.017	0.066	0.059	0.015
Fpz	ORBsup-R	0.045	0.040	0.073	0.029	0.020	0.049
Fpz	ORBsup-L	0.100	0.123	0.091	0.091	0.118	0.071
Fpz	ORBmid-R	0.011	0.004	0.007	0.007	0.002	0.005
Fpz	ORBmid-L	0.026	0.010	0.012	0.021	0.013	0.009
Fpz	SFGmed-R	0.187	0.134	0.084	0.186	0.112	0.113
Fpz	SFGmed-L	0.186	0.173	0.084	0.248	0.247	0.106
Fpz	ORBmed-R	0.127	0.211	0.282	0.126	0.141	0.291
Fpz	ORBmed-L	0.122	0.183	0.277	0.157	0.246	0.261
Fpz	REC-R	0.011	0.012	0.027	0.017	0.017	0.042
AFz	SFGdor-R	0.119	0.044	0.118	0.065	0.020	0.059
AFz	SFGdor-L	0.282	0.195	0.076	0.098	0.105	0.045
AFz	MFG-R	0.011	0.001	0.004	0.007	0.001	0.003
AFz	MFG-L	0.029	0.015	0.006	0.019	0.008	0.004
AFz	SFGmed-R	0.316	0.394	0.456	0.420	0.317	0.536
AFz	SFGmed-L	0.230	0.345	0.333	0.367	0.538	0.343
Fz	SFGdor-R	0.188	0.046	0.113	0.069	0.028	0.071
Fz	SFGdor-L	0.218	0.227	0.090	0.089	0.087	0.045

SD distance = 20 mm		Circumferential SD pair			Vertical SD pair		
10/10 Points	AAL regions	0 year	1 year	2 year	0 year	1 year	2 year
Fz	MFG-R	0.012	0.001	0.005	0.008	0.001	0.004
Fz	MFG-L	0.022	0.005	0.005	0.014	0.004	0.003
Fz	SMA-R	0.025	0.001	0.000	0.020	0.001	0.001
Fz	SMA-L	0.018	0.001	0.000	0.049	0.001	0.000
Fz	SFGmed-R	0.275	0.372	0.439	0.402	0.376	0.558
Fz	SFGmed-L	0.242	0.346	0.348	0.346	0.499	0.317
FCz	SFGdor-R	0.132	0.038	0.096	0.068	0.022	0.058
FCz	SFGdor-L	0.246	0.126	0.059	0.121	0.067	0.038
FCz	MFG-R	0.012	0.002	0.008	0.008	0.001	0.006
FCz	MFG-L	0.011	0.003	0.002	0.008	0.002	0.002
FCz	SMA-R	0.194	0.273	0.173	0.193	0.167	0.230
FCz	SMA-L	0.171	0.243	0.131	0.271	0.304	0.142
FCz	SFGmed-R	0.087	0.143	0.274	0.135	0.146	0.305
FCz	SFGmed-L	0.110	0.170	0.256	0.175	0.288	0.221
FCz	PCL-R	0.025	0.000	0.000	0.006	0.000	0.000
CPz	PreCG-R	0.015	0.002	0.003	0.010	0.002	0.003
CPz	PreCG-L	0.059	0.021	0.022	0.060	0.027	0.024
CPz	SFGdor-L	0.011	0.002	0.002	0.014	0.002	0.003
CPz	SMA-R	0.011	0.004	0.005	0.015	0.007	0.012
CPz	SMA-L	0.015	0.008	0.010	0.028	0.021	0.023
CPz	PoCG-R	0.075	0.019	0.030	0.026	0.009	0.017
CPz	PoCG-L	0.267	0.308	0.240	0.175	0.184	0.119
CPz	SPG-R	0.019	0.014	0.013	0.014	0.007	0.009
CPz	SPG-L	0.069	0.064	0.036	0.103	0.058	0.030
CPz	PCUN-R	0.174	0.198	0.198	0.133	0.152	0.191
CPz	PCUN-L	0.045	0.098	0.072	0.129	0.176	0.112
CPz	PCL-R	0.149	0.147	0.224	0.116	0.166	0.266
CPz	PCL-L	0.080	0.116	0.143	0.168	0.187	0.187
Pz	PoCG-L	0.018	0.006	0.006	0.041	0.009	0.007
Pz	SPG-R	0.052	0.024	0.031	0.024	0.011	0.016
Pz	SPG-L	0.442	0.381	0.250	0.341	0.314	0.156
Pz	PCUN-R	0.306	0.343	0.435	0.262	0.271	0.450

SD distance = 20 mm		Circumferential SD pair			Vertical SD pair		
10/10 Points	AAL regions	0 year	1 year	2 year	0 year	1 year	2 year
Pz	PCUN-L	0.154	0.237	0.270	0.295	0.384	0.362
POz	CUN-R	0.124	0.070	0.088	0.165	0.100	0.128
POz	CUN-L	0.143	0.203	0.146	0.172	0.265	0.148
POz	SOG-R	0.158	0.090	0.129	0.069	0.055	0.081
POz	SOG-L	0.095	0.055	0.025	0.072	0.042	0.014
POz	MOG-L	0.017	0.003	0.002	0.015	0.003	0.001
POz	SPG-R	0.023	0.036	0.046	0.013	0.016	0.023
POz	SPG-L	0.233	0.284	0.244	0.215	0.221	0.177
POz	ANG-L	0.013	0.003	0.003	0.010	0.002	0.001
POz	PCUN-R	0.126	0.171	0.225	0.145	0.168	0.279
POz	PCUN-L	0.058	0.082	0.091	0.118	0.128	0.146
Oz	CAL-R	0.036	0.020	0.028	0.067	0.044	0.064
Oz	CUN-R	0.285	0.311	0.466	0.253	0.323	0.521
Oz	CUN-L	0.145	0.176	0.188	0.264	0.294	0.217
Oz	SOG-R	0.083	0.061	0.089	0.034	0.038	0.056
Oz	SOG-L	0.367	0.386	0.202	0.299	0.262	0.113
Oz	MOG-L	0.059	0.034	0.010	0.039	0.018	0.006
T3	STG-L	0.039	0.072	0.046	0.065	0.198	0.107
T3	TPOsup-L	0.024	0.007	0.008	0.015	0.008	0.008
T3	MTG-L	0.581	0.856	0.831	0.569	0.712	0.765
T3	TPOmid-L	0.053	0.010	0.015	0.022	0.006	0.009
T3	ITG-L	0.296	0.054	0.099	0.323	0.074	0.110
C5	PreCG-L	0.027	0.037	0.024	0.031	0.019	0.016
C5	IFGoperc-L	0.017	0.004	0.003	0.009	0.002	0.002
C5	ROL-L	0.097	0.136	0.083	0.061	0.056	0.033
C5	PoCG-L	0.157	0.406	0.401	0.337	0.436	0.424
C5	SMG-L	0.064	0.114	0.123	0.036	0.085	0.119
C5	STG-L	0.550	0.293	0.353	0.417	0.386	0.385
C5	TPOsup-L	0.030	0.004	0.004	0.020	0.003	0.003
C5	MTG-L	0.046	0.005	0.008	0.080	0.011	0.018
C3	PreCG-L	0.246	0.412	0.340	0.197	0.268	0.264
C3	MFG-L	0.035	0.070	0.060	0.133	0.090	0.094

SD distance = 20 mm		Circumferential SD pair			Vertical SD pair		
10/10 Points	AAL regions	0 year	1 year	2 year	0 year	1 year	2 year
C3	PoCG-L	0.431	0.447	0.475	0.556	0.585	0.531
C3	SMG-L	0.264	0.066	0.116	0.099	0.054	0.104
C1	PreCG-L	0.448	0.419	0.431	0.381	0.478	0.438
C1	SFGdor-L	0.202	0.325	0.251	0.326	0.366	0.326
C1	MFG-L	0.231	0.210	0.268	0.193	0.097	0.182
C1	PoCG-L	0.096	0.036	0.041	0.073	0.044	0.045
C2	PreCG-R	0.448	0.605	0.464	0.372	0.564	0.462
C2	SFGdor-R	0.191	0.134	0.236	0.421	0.268	0.321
C2	MFG-R	0.197	0.167	0.238	0.085	0.073	0.150
C2	SMA-R	0.017	0.007	0.012	0.006	0.013	0.021
C2	PoCG-R	0.119	0.079	0.042	0.106	0.077	0.039
C2	PCL-R	0.012	0.004	0.005	0.004	0.004	0.005
C4	PreCG-R	0.242	0.235	0.343	0.202	0.146	0.329
C4	MFG-R	0.029	0.008	0.025	0.020	0.006	0.021
C4	PoCG-R	0.381	0.539	0.530	0.523	0.718	0.561
C4	IPL-R	0.152	0.159	0.063	0.062	0.054	0.034
C4	SMG-R	0.185	0.058	0.037	0.184	0.076	0.054
C6	PreCG-R	0.013	0.007	0.024	0.023	0.005	0.022
C6	IFGoperc-R	0.019	0.001	0.010	0.011	0.001	0.005
C6	ROL-R	0.069	0.047	0.044	0.034	0.015	0.022
C6	PoCG-R	0.244	0.406	0.514	0.372	0.431	0.521
C6	SMG-R	0.077	0.126	0.136	0.058	0.091	0.090
C6	STG-R	0.375	0.375	0.241	0.246	0.382	0.294
C6	MTG-R	0.176	0.037	0.026	0.235	0.074	0.043
T4	STG-R	0.014	0.014	0.034	0.022	0.023	0.046
T4	MTG-R	0.593	0.786	0.861	0.616	0.767	0.806
T4	TPOmid-R	0.010	0.001	0.001	0.006	0.000	0.001
T4	ITG-R	0.366	0.196	0.094	0.341	0.207	0.138
FT7	IFGoperc-L	0.014	0.013	0.009	0.030	0.014	0.014
FT7	IFGtriang-L	0.038	0.015	0.014	0.049	0.010	0.015
FT7	ORBinf-L	0.146	0.042	0.035	0.088	0.015	0.022
FT7	ROL-L	0.007	0.010	0.005	0.012	0.013	0.007

SD distance = 20 mm		Circumferential SD pair			Vertical SD pair		
10/10 Points	AAL regions	0 year	1 year	2 year	0 year	1 year	2 year
FT7	STG-L	0.053	0.134	0.099	0.033	0.098	0.067
FT7	TPOsup-L	0.307	0.394	0.355	0.407	0.428	0.400
FT7	MTG-L	0.197	0.200	0.268	0.098	0.182	0.195
FT7	TPOmid-L	0.190	0.178	0.202	0.228	0.224	0.265
FT7	ITG-L	0.031	0.009	0.009	0.036	0.012	0.011
F7	MFG-L	0.029	0.008	0.006	0.031	0.009	0.007
F7	ORBmid-L	0.143	0.018	0.025	0.068	0.010	0.013
F7	IFGtriang-L	0.146	0.127	0.082	0.274	0.317	0.172
F7	ORBinf-L	0.593	0.751	0.752	0.565	0.594	0.747
F7	TPOsup-L	0.066	0.083	0.118	0.043	0.058	0.052
F7	TPOmid-L	0.006	0.005	0.011	0.006	0.005	0.006
AF7	SFGdor-L	0.027	0.006	0.006	0.026	0.006	0.007
AF7	ORBsup-L	0.023	0.010	0.014	0.016	0.007	0.011
AF7	MFG-L	0.156	0.109	0.065	0.288	0.247	0.151
AF7	ORBmid-L	0.500	0.548	0.602	0.487	0.501	0.646
AF7	IFGtriang-L	0.071	0.042	0.027	0.051	0.054	0.028
AF7	ORBinf-L	0.213	0.285	0.283	0.125	0.184	0.155
Fp1	SFGdor-L	0.204	0.178	0.077	0.282	0.255	0.196
Fp1	ORBsup-L	0.173	0.320	0.319	0.180	0.267	0.328
Fp1	MFG-L	0.150	0.115	0.035	0.141	0.114	0.049
Fp1	ORBmid-L	0.287	0.321	0.405	0.273	0.321	0.329
Fp1	ORBinf-L	0.011	0.002	0.004	0.009	0.002	0.002
Fp1	SFGmed-R	0.017	0.001	0.003	0.011	0.001	0.003
Fp1	SFGmed-L	0.083	0.029	0.031	0.055	0.019	0.031
Fp1	ORBmed-R	0.011	0.001	0.008	0.007	0.001	0.005
Fp1	ORBmed-L	0.052	0.031	0.112	0.033	0.019	0.052
Fp2	SFGdor-R	0.286	0.243	0.165	0.360	0.360	0.257
Fp2	ORBsup-R	0.136	0.241	0.277	0.157	0.263	0.280
Fp2	MFG-R	0.147	0.077	0.071	0.137	0.058	0.086
Fp2	ORBmid-R	0.166	0.267	0.357	0.184	0.194	0.275
Fp2	ORBinf-R	0.013	0.002	0.007	0.013	0.001	0.005
Fp2	SFGmed-R	0.120	0.054	0.026	0.068	0.047	0.026

SD distance = 20 mm		Circumferential SD pair			Vertical SD pair		
10/10 Points	AAL regions	0 year	1 year	2 year	0 year	1 year	2 year
Fp2	SFGmed-L	0.018	0.003	0.002	0.010	0.002	0.002
Fp2	ORBmed-R	0.079	0.105	0.078	0.047	0.065	0.051
Fp2	ORBmed-L	0.013	0.003	0.005	0.007	0.002	0.004
AF8	SFGdor-R	0.057	0.011	0.012	0.046	0.007	0.010
AF8	ORBsup-R	0.025	0.008	0.014	0.020	0.005	0.009
AF8	MFG-R	0.222	0.155	0.104	0.369	0.234	0.214
AF8	ORBmid-R	0.391	0.601	0.577	0.359	0.546	0.543
AF8	IFGtriang-R	0.098	0.066	0.065	0.066	0.051	0.066
AF8	ORBinf-R	0.195	0.159	0.224	0.130	0.155	0.155
F8	MFG-R	0.035	0.002	0.009	0.038	0.002	0.010
F8	ORBmid-R	0.100	0.008	0.030	0.057	0.004	0.021
F8	IFGoperc-R	0.010	0.006	0.007	0.008	0.008	0.006
F8	IFGtriang-R	0.209	0.166	0.281	0.321	0.306	0.425
F8	ORBinf-R	0.558	0.699	0.519	0.520	0.571	0.430
F8	TPOsup-R	0.063	0.099	0.132	0.039	0.088	0.091
FT8	IFGoperc-R	0.020	0.005	0.014	0.042	0.011	0.022
FT8	IFGtriang-R	0.046	0.008	0.025	0.051	0.011	0.021
FT8	ORBinf-R	0.155	0.016	0.031	0.075	0.013	0.017
FT8	ROL-R	0.016	0.007	0.014	0.030	0.021	0.024
FT8	STG-R	0.085	0.096	0.112	0.101	0.212	0.146
FT8	TPOsup-R	0.220	0.189	0.255	0.262	0.177	0.246
FT8	MTG-R	0.352	0.615	0.499	0.281	0.493	0.460
FT8	TPOmid-R	0.062	0.044	0.036	0.101	0.038	0.048
FT8	ITG-R	0.031	0.018	0.010	0.040	0.018	0.012
TP7	STG-L	0.048	0.062	0.028	0.114	0.122	0.045
TP7	MTG-L	0.601	0.883	0.853	0.580	0.717	0.705
TP7	ITG-L	0.348	0.054	0.118	0.302	0.161	0.248
T5	STG-L	0.018	0.019	0.012	0.046	0.043	0.019
T5	MTG-L	0.761	0.902	0.897	0.697	0.835	0.822
T5	ITG-L	0.208	0.065	0.078	0.245	0.103	0.147
PO7	MOG-L	0.460	0.509	0.517	0.377	0.513	0.569
PO7	IOG-L	0.011	0.004	0.008	0.049	0.007	0.016

SD distance = 20 mm		Circumferential SD pair			Vertical SD pair		
10/10 Points	AAL regions	0 year	1 year	2 year	0 year	1 year	2 year
PO7	ANG-L	0.016	0.031	0.009	0.029	0.049	0.019
PO7	MTG-L	0.497	0.450	0.462	0.502	0.425	0.390
PO7	ITG-L	0.012	0.004	0.004	0.040	0.005	0.004
O1	CUN-L	0.014	0.007	0.012	0.011	0.007	0.010
O1	SOG-L	0.253	0.205	0.272	0.165	0.138	0.225
O1	MOG-L	0.703	0.772	0.700	0.789	0.836	0.747
O2	CAL-R	0.018	0.007	0.002	0.026	0.009	0.003
O2	CUN-R	0.125	0.136	0.059	0.063	0.092	0.041
O2	SOG-R	0.471	0.625	0.504	0.561	0.709	0.534
O2	MOG-R	0.363	0.222	0.427	0.326	0.179	0.413
PO8	SOG-R	0.023	0.029	0.010	0.010	0.016	0.007
PO8	MOG-R	0.864	0.897	0.846	0.923	0.913	0.853
PO8	IOG-R	0.019	0.008	0.003	0.018	0.008	0.004
PO8	ANG-R	0.028	0.036	0.055	0.028	0.044	0.080
PO8	MTG-R	0.060	0.026	0.083	0.016	0.014	0.054
T6	MOG-R	0.160	0.238	0.096	0.096	0.150	0.077
T6	IOG-R	0.072	0.056	0.017	0.171	0.094	0.035
T6	ANG-R	0.019	0.036	0.033	0.045	0.054	0.053
T6	STG-R	0.011	0.006	0.018	0.011	0.006	0.018
T6	MTG-R	0.619	0.629	0.806	0.505	0.655	0.772
T6	ITG-R	0.113	0.030	0.023	0.164	0.034	0.030
TP8	STG-R	0.014	0.020	0.045	0.024	0.041	0.076
TP8	MTG-R	0.717	0.885	0.900	0.620	0.793	0.849
TP8	ITG-R	0.263	0.089	0.049	0.351	0.160	0.069
FC5	PreCG-L	0.088	0.151	0.131	0.145	0.157	0.132
FC5	MFG-L	0.016	0.005	0.006	0.015	0.007	0.005
FC5	IFGoperc-L	0.146	0.247	0.240	0.297	0.475	0.455
FC5	IFGtriang-L	0.435	0.318	0.342	0.290	0.180	0.222
FC5	ORBinf-L	0.033	0.005	0.006	0.032	0.006	0.006
FC5	ROL-L	0.124	0.193	0.195	0.063	0.113	0.103
FC5	PoCG-L	0.057	0.043	0.027	0.032	0.017	0.014
FC5	STG-L	0.034	0.013	0.015	0.023	0.009	0.011

SD distance = 20 mm		Circumferential SD pair			Vertical SD pair		
10/10 Points	AAL regions	0 year	1 year	2 year	0 year	1 year	2 year
FC5	TPOsup-L	0.058	0.022	0.035	0.091	0.034	0.050
FC3	PreCG-L	0.250	0.214	0.169	0.109	0.095	0.083
FC3	MFG-L	0.517	0.652	0.632	0.549	0.658	0.602
FC3	IFGoperc-L	0.120	0.095	0.130	0.160	0.173	0.209
FC3	IFGtriang-L	0.079	0.033	0.059	0.164	0.070	0.099
FC3	PoCG-L	0.026	0.003	0.007	0.011	0.002	0.004
FC1	PreCG-L	0.022	0.003	0.002	0.042	0.002	0.002
FC1	SFGdor-L	0.343	0.461	0.471	0.369	0.480	0.472
FC1	MFG-L	0.585	0.513	0.485	0.540	0.489	0.488
FC1	SMA-L	0.015	0.011	0.011	0.024	0.020	0.016
FC1	SFGmed-L	0.025	0.011	0.028	0.014	0.008	0.020
FC2	PreCG-R	0.046	0.003	0.004	0.028	0.002	0.003
FC2	SFGdor-R	0.350	0.401	0.289	0.363	0.361	0.303
FC2	MFG-R	0.513	0.543	0.674	0.514	0.586	0.658
FC2	SMA-R	0.035	0.020	0.010	0.051	0.030	0.014
FC2	SFGmed-R	0.036	0.031	0.021	0.020	0.018	0.019
FC4	PreCG-R	0.255	0.233	0.161	0.143	0.141	0.083
FC4	MFG-R	0.589	0.698	0.751	0.593	0.760	0.761
FC4	IFGoperc-R	0.050	0.025	0.035	0.098	0.050	0.063
FC4	IFGtriang-R	0.059	0.032	0.044	0.132	0.042	0.086
FC4	PoCG-R	0.036	0.010	0.006	0.024	0.006	0.004
FC6	PreCG-R	0.053	0.101	0.096	0.111	0.233	0.110
FC6	MFG-R	0.029	0.008	0.018	0.039	0.012	0.020
FC6	IFGoperc-R	0.171	0.217	0.229	0.258	0.238	0.334
FC6	IFGtriang-R	0.363	0.246	0.424	0.274	0.119	0.320
FC6	ORBinf-R	0.020	0.002	0.002	0.021	0.002	0.002
FC6	ROL-R	0.075	0.090	0.061	0.064	0.143	0.080
FC6	PoCG-R	0.167	0.293	0.136	0.097	0.180	0.081
FC6	STG-R	0.064	0.031	0.021	0.062	0.049	0.029
FC6	TPOsup-R	0.030	0.009	0.010	0.049	0.019	0.021
FC6	MTG-R	0.019	0.003	0.002	0.019	0.005	0.003
F5	MFG-L	0.303	0.262	0.283	0.332	0.281	0.211

SD distance = 20 mm		Circumferential SD pair			Vertical SD pair		
10/10 Points	AAL regions	0 year	1 year	2 year	0 year	1 year	2 year
F5	ORBmid-L	0.035	0.006	0.009	0.035	0.007	0.008
F5	IFGoperc-L	0.018	0.009	0.006	0.009	0.004	0.004
F5	IFGtriang-L	0.554	0.689	0.659	0.473	0.646	0.678
F5	ORBinf-L	0.072	0.030	0.040	0.138	0.060	0.097
F3	SFGdor-L	0.031	0.009	0.018	0.032	0.012	0.015
F3	MFG-L	0.792	0.922	0.926	0.892	0.957	0.940
F3	IFGoperc-L	0.011	0.002	0.002	0.004	0.001	0.001
F3	IFGtriang-L	0.154	0.065	0.051	0.058	0.029	0.041
F1	SFGdor-L	0.294	0.489	0.474	0.464	0.574	0.533
F1	MFG-L	0.543	0.391	0.371	0.414	0.373	0.389
F1	SFGmed-R	0.024	0.005	0.013	0.021	0.003	0.008
F1	SFGmed-L	0.129	0.115	0.140	0.091	0.049	0.068
F2	SFGdor-R	0.299	0.578	0.585	0.547	0.725	0.627
F2	MFG-R	0.269	0.220	0.288	0.288	0.159	0.289
F2	SFGmed-R	0.384	0.193	0.119	0.128	0.110	0.078
F2	SFGmed-L	0.030	0.008	0.008	0.021	0.005	0.006
F4	SFGdor-R	0.072	0.032	0.027	0.067	0.021	0.024
F4	MFG-R	0.811	0.922	0.890	0.842	0.924	0.909
F4	IFGtriang-R	0.093	0.043	0.079	0.072	0.052	0.065
F6	MFG-R	0.353	0.191	0.214	0.331	0.164	0.182
F6	ORBmid-R	0.024	0.009	0.011	0.034	0.012	0.012
F6	IFGoperc-R	0.027	0.013	0.005	0.015	0.007	0.004
F6	IFGtriang-R	0.534	0.765	0.755	0.523	0.774	0.777
F6	ORBinf-R	0.037	0.018	0.011	0.077	0.039	0.021
AF3	SFGdor-L	0.357	0.428	0.461	0.352	0.293	0.436
AF3	ORBsup-L	0.013	0.009	0.015	0.024	0.014	0.032
AF3	MFG-L	0.493	0.524	0.443	0.504	0.652	0.452
AF3	ORBmid-L	0.027	0.013	0.021	0.047	0.027	0.043
AF3	SFGmed-R	0.014	0.001	0.003	0.010	0.001	0.002
AF3	SFGmed-L	0.078	0.023	0.054	0.048	0.011	0.030
AF4	SFGdor-R	0.393	0.489	0.351	0.409	0.522	0.315
AF4	ORBsup-R	0.011	0.004	0.006	0.018	0.006	0.012

SD distance = 20 mm		Circumferential SD pair			Vertical SD pair		
10/10 Points	AAL regions	0 year	1 year	2 year	0 year	1 year	2 year
AF4	MFG-R	0.421	0.441	0.594	0.446	0.422	0.621
AF4	ORBmid-R	0.020	0.006	0.015	0.037	0.012	0.027
AF4	IFGtriang-R	0.013	0.002	0.005	0.009	0.001	0.003
AF4	SFGmed-R	0.116	0.056	0.026	0.060	0.033	0.019
AF4	SFGmed-L	0.012	0.002	0.001	0.007	0.001	0.001
CP5	PoCG-L	0.023	0.006	0.007	0.015	0.005	0.005
CP5	SMG-L	0.242	0.529	0.419	0.353	0.500	0.409
CP5	ANG-L	0.017	0.030	0.022	0.004	0.010	0.010
CP5	STG-L	0.625	0.409	0.513	0.445	0.441	0.506
CP5	MTG-L	0.089	0.021	0.035	0.180	0.041	0.068
CP3	PoCG-L	0.156	0.151	0.095	0.164	0.142	0.103
CP3	SPG-L	0.019	0.017	0.020	0.013	0.013	0.021
CP3	IPL-L	0.427	0.554	0.580	0.264	0.493	0.598
CP3	SMG-L	0.381	0.272	0.292	0.537	0.344	0.261
CP1	PreCG-L	0.143	0.077	0.091	0.215	0.088	0.099
CP1	PoCG-L	0.492	0.608	0.577	0.399	0.553	0.550
CP1	SPG-L	0.305	0.286	0.300	0.285	0.321	0.296
CP1	IPL-L	0.034	0.021	0.022	0.071	0.031	0.045
CP2	PreCG-R	0.113	0.043	0.062	0.114	0.043	0.081
CP2	SFGdor-R	0.010	0.002	0.004	0.015	0.002	0.005
CP2	PoCG-R	0.467	0.542	0.567	0.434	0.497	0.583
CP2	SPG-R	0.301	0.328	0.299	0.294	0.369	0.256
CP2	IPL-R	0.023	0.019	0.024	0.046	0.029	0.033
CP2	PCUN-R	0.048	0.044	0.022	0.031	0.026	0.013
CP2	PCL-R	0.024	0.019	0.019	0.048	0.029	0.026
CP4	PreCG-R	0.012	0.001	0.007	0.010	0.002	0.008
CP4	PoCG-R	0.207	0.162	0.223	0.229	0.199	0.263
CP4	SPG-R	0.020	0.014	0.011	0.014	0.014	0.008
CP4	IPL-R	0.682	0.801	0.738	0.637	0.762	0.695
CP4	SMG-R	0.046	0.008	0.013	0.075	0.009	0.020
CP4	ANG-R	0.029	0.014	0.007	0.032	0.014	0.006
CP6	PoCG-R	0.027	0.007	0.015	0.014	0.004	0.012

SD distance = 20 mm		Circumferential SD pair			Vertical SD pair		
10/10 Points	AAL regions	0 year	1 year	2 year	0 year	1 year	2 year
CP6	IPL-R	0.043	0.080	0.068	0.077	0.101	0.091
CP6	SMG-R	0.462	0.606	0.720	0.430	0.495	0.665
CP6	ANG-R	0.057	0.046	0.024	0.019	0.018	0.013
CP6	STG-R	0.264	0.228	0.152	0.233	0.322	0.195
CP6	MTG-R	0.145	0.032	0.021	0.223	0.060	0.024
P5	MOG-L	0.047	0.018	0.025	0.016	0.014	0.022
P5	IPL-L	0.013	0.048	0.038	0.053	0.119	0.077
P5	SMG-L	0.078	0.044	0.033	0.053	0.027	0.018
P5	ANG-L	0.360	0.711	0.678	0.392	0.550	0.599
P5	STG-L	0.241	0.086	0.076	0.099	0.055	0.030
P5	MTG-L	0.260	0.094	0.150	0.386	0.235	0.254
P3	SPG-L	0.052	0.057	0.040	0.049	0.057	0.043
P3	IPL-L	0.476	0.476	0.363	0.294	0.319	0.319
P3	SMG-L	0.023	0.004	0.003	0.008	0.003	0.002
P3	ANG-L	0.438	0.458	0.589	0.624	0.616	0.631
P1	PoCG-L	0.026	0.008	0.006	0.019	0.009	0.007
P1	SPG-L	0.762	0.883	0.847	0.735	0.856	0.848
P1	IPL-L	0.088	0.044	0.056	0.041	0.033	0.039
P1	ANG-L	0.078	0.052	0.069	0.177	0.092	0.090
P1	PCUN-L	0.027	0.010	0.015	0.012	0.006	0.010
P2	SOG-R	0.010	0.003	0.001	0.033	0.004	0.002
P2	PoCG-R	0.022	0.002	0.007	0.019	0.002	0.006
P2	SPG-R	0.597	0.661	0.732	0.603	0.715	0.775
P2	IPL-R	0.038	0.014	0.025	0.044	0.018	0.030
P2	ANG-R	0.032	0.011	0.015	0.025	0.008	0.016
P2	PCUN-R	0.265	0.298	0.211	0.245	0.244	0.164
P2	PCUN-L	0.016	0.008	0.006	0.012	0.006	0.004
P4	MOG-R	0.030	0.006	0.007	0.111	0.014	0.012
P4	SPG-R	0.114	0.203	0.149	0.118	0.186	0.138
P4	IPL-R	0.330	0.235	0.324	0.140	0.150	0.274
P4	ANG-R	0.501	0.550	0.512	0.617	0.645	0.569
P6	MOG-R	0.133	0.060	0.025	0.076	0.049	0.020

SD distance = 20 mm		Circumferential SD pair			Vertical SD pair		
10/10 Points	AAL regions	0 year	1 year	2 year	0 year	1 year	2 year
P6	IPL-R	0.048	0.052	0.068	0.082	0.054	0.093
P6	SMG-R	0.123	0.041	0.078	0.034	0.016	0.045
P6	ANG-R	0.572	0.799	0.790	0.669	0.812	0.777
P6	STG-R	0.018	0.003	0.003	0.006	0.002	0.003
P6	MTG-R	0.103	0.043	0.035	0.129	0.065	0.062
PO3	CUN-L	0.014	0.010	0.010	0.010	0.006	0.006
PO3	SOG-L	0.134	0.236	0.198	0.118	0.141	0.138
PO3	MOG-L	0.311	0.174	0.227	0.450	0.297	0.312
PO3	SPG-L	0.049	0.080	0.061	0.111	0.103	0.084
PO3	ANG-L	0.482	0.495	0.500	0.303	0.449	0.455
PO4	CUN-R	0.029	0.017	0.008	0.026	0.012	0.007
PO4	SOG-R	0.295	0.426	0.309	0.422	0.409	0.254
PO4	MOG-R	0.424	0.259	0.289	0.243	0.263	0.325
PO4	SPG-R	0.039	0.063	0.067	0.110	0.123	0.096
PO4	IPL-R	0.147	0.203	0.277	0.142	0.168	0.284
PO4	ANG-R	0.037	0.018	0.038	0.022	0.013	0.023
PO4	PCUN-R	0.017	0.010	0.009	0.023	0.009	0.008

Table S6 Normalized PPL of corresponding brain regions ($L_{norm,M}$) for the circumferential and vertical SD pairs set at 10/10 fiducial points at 25-mm SD distance from 0-yo, 1-yo, and 2-yo.

SD distance = 25 mm		Circumferential SD pair			Vertical SD pair		
10/10 Points	AAL regions	0 year	1 year	2 year	0 year	1 year	2 year
Cz	PreCG-R	0.030	0.011	0.010	0.017	0.005	0.007
Cz	PreCG-L	0.081	0.035	0.017	0.078	0.039	0.020
Cz	SFGdor-R	0.206	0.092	0.107	0.060	0.024	0.042
Cz	SFGdor-L	0.247	0.227	0.158	0.120	0.114	0.071
Cz	SMA-R	0.145	0.320	0.366	0.175	0.255	0.408
Cz	SMA-L	0.123	0.246	0.295	0.231	0.435	0.348
Cz	PoCG-R	0.011	0.002	0.001	0.011	0.002	0.002
Cz	PoCG-L	0.033	0.005	0.002	0.050	0.009	0.004
Cz	SPG-L	0.033	0.000	0.000	0.017	0.000	0.000
Cz	PCL-R	0.054	0.045	0.027	0.093	0.073	0.069
Cz	PCL-L	0.019	0.011	0.007	0.118	0.037	0.021
Fpz	SFGdor-R	0.077	0.044	0.041	0.036	0.018	0.032
Fpz	SFGdor-L	0.122	0.084	0.020	0.071	0.053	0.018
Fpz	ORBsup-R	0.054	0.048	0.093	0.030	0.031	0.049
Fpz	ORBsup-L	0.111	0.150	0.111	0.093	0.112	0.072
Fpz	MFG-L	0.015	0.002	0.001	0.009	0.001	0.001
Fpz	ORBmid-R	0.015	0.005	0.011	0.007	0.003	0.006
Fpz	ORBmid-L	0.035	0.016	0.017	0.024	0.014	0.010
Fpz	SFGmed-R	0.166	0.131	0.079	0.204	0.121	0.153
Fpz	SFGmed-L	0.162	0.153	0.080	0.244	0.229	0.126
Fpz	ORBmed-R	0.112	0.191	0.252	0.113	0.161	0.253
Fpz	ORBmed-L	0.102	0.157	0.255	0.134	0.215	0.221
Fpz	REC-R	0.012	0.013	0.029	0.019	0.030	0.044
AFz	SFGdor-R	0.177	0.071	0.154	0.073	0.025	0.067
AFz	SFGdor-L	0.221	0.237	0.106	0.104	0.093	0.048
AFz	MFG-R	0.020	0.002	0.006	0.009	0.001	0.003
AFz	MFG-L	0.055	0.028	0.009	0.022	0.009	0.005
AFz	SFGmed-R	0.292	0.363	0.410	0.407	0.347	0.525
AFz	SFGmed-L	0.209	0.292	0.308	0.347	0.510	0.335
Fz	SFGdor-R	0.164	0.086	0.158	0.069	0.032	0.079

SD distance = 25 mm		Circumferential SD pair			Vertical SD pair		
10/10 Points	AAL regions	0 year	1 year	2 year	0 year	1 year	2 year
Fz	SFGdor-L	0.362	0.236	0.131	0.103	0.093	0.051
Fz	MFG-R	0.018	0.001	0.008	0.009	0.001	0.005
Fz	MFG-L	0.035	0.008	0.007	0.018	0.005	0.004
Fz	SMA-R	0.017	0.002	0.001	0.024	0.002	0.001
Fz	SMA-L	0.012	0.002	0.000	0.034	0.002	0.001
Fz	SFGmed-R	0.205	0.371	0.384	0.370	0.379	0.542
Fz	SFGmed-L	0.185	0.294	0.311	0.372	0.483	0.317
FCz	PreCG-L	0.012	0.000	0.000	0.009	0.000	0.000
FCz	SFGdor-R	0.157	0.064	0.134	0.075	0.027	0.066
FCz	SFGdor-L	0.276	0.197	0.085	0.135	0.075	0.041
FCz	MFG-R	0.016	0.004	0.012	0.010	0.002	0.007
FCz	MFG-L	0.016	0.006	0.004	0.010	0.003	0.002
FCz	SMA-R	0.168	0.188	0.151	0.186	0.188	0.249
FCz	SMA-L	0.160	0.228	0.116	0.241	0.318	0.156
FCz	SFGmed-R	0.081	0.164	0.247	0.143	0.148	0.282
FCz	SFGmed-L	0.095	0.150	0.251	0.171	0.237	0.198
FCz	PCL-R	0.014	0.000	0.000	0.010	0.000	0.000
CPz	PreCG-R	0.020	0.003	0.005	0.011	0.003	0.004
CPz	PreCG-L	0.066	0.023	0.031	0.076	0.034	0.027
CPz	SFGdor-R	0.011	0.001	0.002	0.012	0.002	0.002
CPz	SFGdor-L	0.012	0.002	0.003	0.023	0.004	0.004
CPz	SMA-R	0.012	0.004	0.007	0.022	0.011	0.014
CPz	SMA-L	0.015	0.009	0.012	0.049	0.032	0.027
CPz	PoCG-R	0.130	0.031	0.044	0.026	0.010	0.017
CPz	PoCG-L	0.247	0.297	0.250	0.167	0.172	0.117
CPz	SPG-R	0.036	0.021	0.016	0.014	0.009	0.010
CPz	SPG-L	0.081	0.066	0.038	0.126	0.076	0.037
CPz	PCUN-R	0.138	0.201	0.173	0.124	0.152	0.200
CPz	PCUN-L	0.040	0.084	0.058	0.121	0.166	0.119
CPz	PCL-R	0.125	0.157	0.230	0.112	0.165	0.247
CPz	PCL-L	0.066	0.100	0.130	0.114	0.166	0.174
Pz	PoCG-L	0.025	0.008	0.008	0.063	0.014	0.010

SD distance = 25 mm		Circumferential SD pair			Vertical SD pair		
10/10 Points	AAL regions	0 year	1 year	2 year	0 year	1 year	2 year
Pz	SPG-R	0.095	0.034	0.044	0.025	0.013	0.018
Pz	SPG-L	0.424	0.413	0.279	0.347	0.305	0.157
Pz	PCUN-R	0.296	0.340	0.424	0.242	0.283	0.446
Pz	PCUN-L	0.125	0.196	0.236	0.269	0.372	0.358
POz	CUN-R	0.100	0.068	0.083	0.178	0.114	0.145
POz	CUN-L	0.117	0.190	0.139	0.163	0.252	0.155
POz	SOG-R	0.170	0.104	0.148	0.068	0.056	0.078
POz	SOG-L	0.120	0.074	0.030	0.078	0.043	0.018
POz	MOG-L	0.023	0.004	0.002	0.016	0.003	0.001
POz	SPG-R	0.029	0.044	0.053	0.014	0.018	0.022
POz	SPG-L	0.243	0.284	0.245	0.196	0.193	0.159
POz	ANG-L	0.021	0.005	0.004	0.010	0.002	0.001
POz	PCUN-R	0.114	0.153	0.208	0.148	0.177	0.269
POz	PCUN-L	0.052	0.070	0.083	0.121	0.140	0.150
Oz	CAL-R	0.037	0.023	0.029	0.083	0.051	0.072
Oz	CUN-R	0.244	0.282	0.414	0.239	0.285	0.495
Oz	CUN-L	0.125	0.144	0.165	0.245	0.296	0.223
Oz	SOG-R	0.113	0.095	0.126	0.040	0.039	0.059
Oz	SOG-L	0.358	0.391	0.231	0.280	0.278	0.115
Oz	MOG-L	0.093	0.051	0.016	0.048	0.025	0.007
T3	STG-L	0.042	0.083	0.058	0.110	0.292	0.184
T3	TPOsup-L	0.028	0.011	0.011	0.023	0.013	0.010
T3	MTG-L	0.521	0.823	0.799	0.504	0.596	0.671
T3	TPOmid-L	0.061	0.018	0.021	0.027	0.008	0.009
T3	ITG-L	0.340	0.064	0.109	0.326	0.086	0.122
C5	PreCG-L	0.032	0.045	0.042	0.039	0.027	0.030
C5	IFGoperc-L	0.027	0.008	0.008	0.010	0.003	0.004
C5	IFGtriang-L	0.013	0.001	0.001	0.005	0.000	0.001
C5	ROL-L	0.090	0.183	0.120	0.047	0.052	0.041
C5	PoCG-L	0.138	0.300	0.324	0.367	0.416	0.431
C5	SMG-L	0.064	0.087	0.109	0.055	0.080	0.091
C5	STG-L	0.534	0.360	0.374	0.341	0.399	0.371

SD distance = 25 mm		Circumferential SD pair			Vertical SD pair		
10/10 Points	AAL regions	0 year	1 year	2 year	0 year	1 year	2 year
C5	TPOsup-L	0.035	0.007	0.008	0.018	0.004	0.004
C5	MTG-L	0.060	0.008	0.011	0.110	0.019	0.026
C3	PreCG-L	0.263	0.368	0.338	0.188	0.266	0.271
C3	MFG-L	0.053	0.076	0.064	0.114	0.077	0.116
C3	IFGoperc-L	0.018	0.004	0.004	0.005	0.001	0.002
C3	PoCG-L	0.350	0.417	0.426	0.505	0.586	0.498
C3	IPL-L	0.012	0.005	0.008	0.005	0.002	0.003
C3	SMG-L	0.291	0.128	0.157	0.166	0.067	0.108
C1	PreCG-L	0.411	0.423	0.420	0.322	0.458	0.430
C1	SFGdor-L	0.196	0.257	0.220	0.284	0.362	0.314
C1	MFG-L	0.236	0.258	0.289	0.214	0.102	0.182
C1	SMA-L	0.011	0.010	0.006	0.021	0.018	0.009
C1	PoCG-L	0.127	0.049	0.061	0.105	0.056	0.061
C2	PreCG-R	0.377	0.510	0.432	0.339	0.513	0.453
C2	SFGdor-R	0.184	0.141	0.230	0.340	0.284	0.318
C2	MFG-R	0.207	0.216	0.257	0.074	0.074	0.145
C2	SMA-R	0.022	0.010	0.016	0.048	0.022	0.027
C2	PoCG-R	0.166	0.112	0.054	0.152	0.097	0.048
C2	PCL-R	0.018	0.007	0.007	0.020	0.007	0.006
C4	PreCG-R	0.250	0.241	0.376	0.228	0.188	0.331
C4	MFG-R	0.045	0.011	0.037	0.028	0.009	0.024
C4	PoCG-R	0.340	0.493	0.472	0.467	0.671	0.534
C4	IPL-R	0.192	0.180	0.074	0.063	0.046	0.038
C4	SMG-R	0.157	0.072	0.038	0.199	0.084	0.070
C6	PreCG-R	0.016	0.009	0.028	0.030	0.008	0.037
C6	IFGoperc-R	0.032	0.002	0.014	0.011	0.001	0.007
C6	IFGtriang-R	0.014	0.001	0.002	0.007	0.000	0.002
C6	ROL-R	0.066	0.061	0.048	0.029	0.013	0.028
C6	PoCG-R	0.206	0.358	0.462	0.360	0.440	0.513
C6	SMG-R	0.081	0.120	0.152	0.066	0.102	0.067
C6	STG-R	0.349	0.396	0.257	0.205	0.320	0.284
C6	TPOsup-R	0.011	0.001	0.001	0.005	0.000	0.001

SD distance = 25 mm		Circumferential SD pair			Vertical SD pair		
10/10 Points	AAL regions	0 year	1 year	2 year	0 year	1 year	2 year
C6	MTG-R	0.210	0.052	0.032	0.272	0.113	0.060
T4	STG-R	0.021	0.020	0.037	0.038	0.037	0.069
T4	TPOsup-R	0.013	0.002	0.007	0.011	0.001	0.004
T4	MTG-R	0.611	0.786	0.824	0.587	0.729	0.745
T4	TPOmid-R	0.013	0.001	0.003	0.009	0.001	0.001
T4	ITG-R	0.332	0.188	0.124	0.340	0.229	0.172
FT7	IFGoperc-L	0.015	0.016	0.012	0.033	0.031	0.021
FT7	IFGtriang-L	0.043	0.025	0.021	0.035	0.019	0.021
FT7	ORBinf-L	0.159	0.075	0.060	0.054	0.023	0.026
FT7	ROL-L	0.008	0.015	0.006	0.021	0.039	0.012
FT7	STG-L	0.066	0.164	0.102	0.060	0.105	0.068
FT7	TPOsup-L	0.262	0.361	0.359	0.389	0.404	0.395
FT7	MTG-L	0.243	0.201	0.258	0.123	0.163	0.195
FT7	TPOmid-L	0.148	0.127	0.170	0.215	0.192	0.243
FT7	ITG-L	0.039	0.009	0.009	0.047	0.016	0.016
F7	MFG-L	0.031	0.011	0.007	0.035	0.012	0.009
F7	ORBmid-L	0.163	0.034	0.029	0.062	0.013	0.014
F7	IFGtriang-L	0.138	0.151	0.096	0.320	0.360	0.279
F7	ORBinf-L	0.539	0.670	0.653	0.492	0.536	0.612
F7	TPOsup-L	0.094	0.111	0.185	0.059	0.061	0.071
F7	TPOmid-L	0.010	0.008	0.019	0.011	0.006	0.010
AF7	SFGdor-L	0.038	0.010	0.008	0.030	0.007	0.008
AF7	ORBsup-L	0.034	0.018	0.021	0.018	0.008	0.013
AF7	MFG-L	0.166	0.128	0.072	0.313	0.270	0.207
AF7	ORBmid-L	0.416	0.492	0.553	0.433	0.471	0.571
AF7	IFGtriang-L	0.095	0.056	0.034	0.062	0.057	0.039
AF7	ORBinf-L	0.237	0.295	0.306	0.135	0.186	0.158
Fp1	SFGdor-L	0.187	0.171	0.085	0.302	0.268	0.215
Fp1	ORBsup-L	0.160	0.305	0.291	0.162	0.249	0.311
Fp1	MFG-L	0.150	0.094	0.045	0.140	0.121	0.056
Fp1	ORBmid-L	0.280	0.339	0.393	0.248	0.309	0.309
Fp1	ORBinf-L	0.013	0.003	0.004	0.009	0.003	0.003

SD distance = 25 mm		Circumferential SD pair			Vertical SD pair		
10/10 Points	AAL regions	0 year	1 year	2 year	0 year	1 year	2 year
Fp1	SFGmed-R	0.022	0.002	0.004	0.015	0.001	0.003
Fp1	SFGmed-L	0.094	0.039	0.037	0.067	0.022	0.034
Fp1	ORBmed-R	0.015	0.002	0.010	0.008	0.001	0.006
Fp1	ORBmed-L	0.062	0.043	0.123	0.036	0.022	0.056
Fp2	SFGdor-R	0.241	0.228	0.155	0.341	0.359	0.287
Fp2	ORBsup-R	0.119	0.206	0.247	0.157	0.259	0.251
Fp2	MFG-R	0.161	0.094	0.080	0.116	0.059	0.108
Fp2	ORBmid-R	0.166	0.272	0.347	0.155	0.170	0.242
Fp2	ORBinf-R	0.016	0.003	0.009	0.012	0.001	0.005
Fp2	SFGmed-R	0.134	0.065	0.033	0.104	0.056	0.032
Fp2	SFGmed-L	0.027	0.004	0.003	0.017	0.003	0.003
Fp2	ORBmed-R	0.087	0.116	0.103	0.061	0.076	0.053
Fp2	ORBmed-L	0.020	0.005	0.008	0.012	0.004	0.005
Fp2	REC-R	0.008	0.006	0.011	0.011	0.010	0.012
AF8	SFGdor-R	0.079	0.018	0.017	0.050	0.010	0.011
AF8	ORBsup-R	0.038	0.014	0.020	0.023	0.007	0.010
AF8	MFG-R	0.210	0.162	0.115	0.364	0.266	0.247
AF8	ORBmid-R	0.338	0.555	0.531	0.338	0.494	0.486
AF8	IFGtriang-R	0.114	0.075	0.084	0.074	0.062	0.085
AF8	ORBinf-R	0.201	0.174	0.226	0.138	0.158	0.158
F8	MFG-R	0.042	0.004	0.010	0.045	0.002	0.012
F8	ORBmid-R	0.119	0.017	0.037	0.057	0.005	0.020
F8	IFGoperc-R	0.015	0.008	0.008	0.010	0.013	0.008
F8	IFGtriang-R	0.195	0.169	0.251	0.334	0.332	0.456
F8	ORBinf-R	0.494	0.633	0.500	0.478	0.505	0.372
F8	STG-R	0.011	0.008	0.005	0.004	0.005	0.003
F8	TPOsup-R	0.091	0.135	0.162	0.050	0.113	0.107
F8	TPOmid-R	0.008	0.010	0.012	0.007	0.013	0.012
FT8	IFGoperc-R	0.018	0.007	0.020	0.048	0.014	0.033
FT8	IFGtriang-R	0.049	0.014	0.039	0.047	0.013	0.029
FT8	ORBinf-R	0.189	0.032	0.048	0.065	0.013	0.020
FT8	ROL-R	0.015	0.011	0.019	0.039	0.033	0.038

SD distance = 25 mm		Circumferential SD pair			Vertical SD pair		
10/10 Points	AAL regions	0 year	1 year	2 year	0 year	1 year	2 year
FT8	STG-R	0.076	0.105	0.122	0.111	0.217	0.148
FT8	TPOsup-R	0.195	0.213	0.274	0.232	0.159	0.247
FT8	MTG-R	0.349	0.551	0.429	0.283	0.476	0.408
FT8	TPOmid-R	0.057	0.043	0.034	0.105	0.040	0.054
FT8	ITG-R	0.036	0.018	0.009	0.049	0.028	0.015
TP7	STG-L	0.062	0.066	0.034	0.171	0.188	0.066
TP7	MTG-L	0.585	0.851	0.822	0.520	0.618	0.664
TP7	ITG-L	0.348	0.082	0.143	0.303	0.192	0.269
T5	MOG-L	0.015	0.008	0.011	0.003	0.006	0.005
T5	STG-L	0.025	0.030	0.015	0.101	0.080	0.029
T5	MTG-L	0.759	0.881	0.875	0.629	0.758	0.769
T5	ITG-L	0.190	0.071	0.091	0.251	0.131	0.186
PO7	MOG-L	0.436	0.516	0.535	0.383	0.535	0.590
PO7	IOG-L	0.017	0.004	0.010	0.090	0.012	0.026
PO7	ANG-L	0.019	0.045	0.010	0.060	0.088	0.031
PO7	MTG-L	0.501	0.428	0.438	0.415	0.358	0.346
PO7	ITG-L	0.018	0.004	0.005	0.047	0.006	0.006
O1	CUN-L	0.023	0.012	0.018	0.016	0.010	0.012
O1	SOG-L	0.238	0.270	0.293	0.176	0.162	0.219
O1	MOG-L	0.684	0.694	0.668	0.726	0.798	0.744
O1	ANG-L	0.011	0.011	0.005	0.017	0.015	0.008
O1	MTG-L	0.018	0.004	0.003	0.002	0.002	0.002
O2	CAL-R	0.028	0.010	0.003	0.041	0.016	0.004
O2	CUN-R	0.172	0.172	0.081	0.065	0.103	0.045
O2	CUN-L	0.018	0.009	0.004	0.008	0.007	0.003
O2	SOG-R	0.377	0.535	0.471	0.473	0.645	0.502
O2	MOG-R	0.383	0.264	0.433	0.385	0.219	0.437
PO8	SOG-R	0.037	0.040	0.013	0.013	0.018	0.009
PO8	MOG-R	0.793	0.871	0.811	0.859	0.887	0.838
PO8	IOG-R	0.023	0.009	0.005	0.032	0.013	0.005
PO8	ANG-R	0.041	0.041	0.057	0.067	0.059	0.095
PO8	MTG-R	0.098	0.034	0.111	0.019	0.017	0.049

SD distance = 25 mm		Circumferential SD pair			Vertical SD pair		
10/10 Points	AAL regions	0 year	1 year	2 year	0 year	1 year	2 year
T6	MOG-R	0.178	0.219	0.121	0.102	0.165	0.092
T6	IOG-R	0.079	0.045	0.018	0.238	0.129	0.057
T6	ANG-R	0.018	0.044	0.039	0.062	0.081	0.063
T6	STG-R	0.015	0.010	0.025	0.012	0.008	0.022
T6	MTG-R	0.572	0.645	0.766	0.420	0.568	0.701
T6	ITG-R	0.132	0.030	0.022	0.151	0.038	0.041
TP8	STG-R	0.020	0.025	0.051	0.050	0.067	0.100
TP8	MTG-R	0.673	0.851	0.880	0.593	0.741	0.797
TP8	ITG-R	0.296	0.115	0.062	0.347	0.183	0.094
FC5	PreCG-L	0.071	0.151	0.133	0.138	0.143	0.159
FC5	MFG-L	0.019	0.008	0.008	0.024	0.013	0.007
FC5	IFGoperc-L	0.111	0.203	0.208	0.276	0.439	0.387
FC5	IFGtriang-L	0.384	0.349	0.349	0.286	0.239	0.196
FC5	ORBinf-L	0.048	0.007	0.008	0.046	0.011	0.008
FC5	ROL-L	0.108	0.165	0.170	0.051	0.087	0.117
FC5	PoCG-L	0.111	0.077	0.057	0.034	0.017	0.022
FC5	STG-L	0.062	0.016	0.023	0.023	0.009	0.018
FC5	TPOsup-L	0.069	0.023	0.040	0.104	0.041	0.083
FC3	PreCG-L	0.256	0.252	0.212	0.144	0.118	0.091
FC3	MFG-L	0.538	0.602	0.593	0.476	0.586	0.541
FC3	IFGoperc-L	0.087	0.092	0.120	0.165	0.183	0.206
FC3	IFGtriang-L	0.074	0.046	0.060	0.184	0.106	0.152
FC3	PoCG-L	0.034	0.005	0.010	0.018	0.003	0.005
FC1	PreCG-L	0.030	0.005	0.004	0.039	0.003	0.003
FC1	SFGdor-L	0.311	0.459	0.458	0.341	0.445	0.443
FC1	MFG-L	0.568	0.497	0.478	0.546	0.516	0.503
FC1	SMA-L	0.022	0.017	0.014	0.037	0.025	0.024
FC1	SFGmed-L	0.050	0.020	0.041	0.019	0.009	0.022
FC2	PreCG-R	0.060	0.005	0.006	0.025	0.003	0.004
FC2	SFGdor-R	0.332	0.415	0.326	0.308	0.349	0.289
FC2	MFG-R	0.470	0.510	0.614	0.468	0.580	0.668
FC2	SMA-R	0.047	0.022	0.013	0.070	0.046	0.018

SD distance = 25 mm		Circumferential SD pair			Vertical SD pair		
10/10 Points	AAL regions	0 year	1 year	2 year	0 year	1 year	2 year
FC2	SFGmed-R	0.060	0.046	0.035	0.022	0.018	0.018
FC4	PreCG-R	0.249	0.267	0.191	0.168	0.168	0.104
FC4	MFG-R	0.580	0.652	0.715	0.584	0.713	0.706
FC4	IFGoperc-R	0.044	0.026	0.032	0.080	0.051	0.065
FC4	IFGtriang-R	0.061	0.037	0.049	0.123	0.057	0.116
FC4	PoCG-R	0.049	0.017	0.009	0.028	0.009	0.006
FC6	PreCG-R	0.054	0.093	0.099	0.127	0.241	0.117
FC6	MFG-R	0.039	0.009	0.019	0.050	0.019	0.027
FC6	IFGoperc-R	0.133	0.180	0.201	0.227	0.204	0.290
FC6	IFGtriang-R	0.358	0.256	0.408	0.265	0.131	0.339
FC6	ORBinf-R	0.025	0.002	0.002	0.024	0.003	0.004
FC6	ROL-R	0.067	0.094	0.064	0.060	0.133	0.078
FC6	PoCG-R	0.187	0.307	0.167	0.095	0.160	0.076
FC6	STG-R	0.074	0.042	0.026	0.064	0.069	0.035
FC6	TPOsup-R	0.029	0.011	0.010	0.057	0.033	0.029
FC6	MTG-R	0.022	0.005	0.003	0.022	0.008	0.004
F5	MFG-L	0.262	0.296	0.297	0.351	0.243	0.244
F5	ORBmid-L	0.045	0.009	0.013	0.045	0.008	0.013
F5	IFGoperc-L	0.040	0.019	0.008	0.017	0.008	0.005
F5	IFGtriang-L	0.522	0.630	0.627	0.430	0.641	0.598
F5	ORBinf-L	0.093	0.039	0.049	0.140	0.096	0.136
F5	TPOsup-L	0.014	0.003	0.003	0.006	0.002	0.002
F3	SFGdor-L	0.050	0.014	0.025	0.044	0.017	0.021
F3	MFG-L	0.750	0.890	0.893	0.856	0.942	0.924
F3	IFGoperc-L	0.016	0.004	0.004	0.006	0.001	0.002
F3	IFGtriang-L	0.165	0.090	0.075	0.072	0.037	0.049
F1	SFGdor-L	0.262	0.448	0.410	0.435	0.497	0.505
F1	MFG-L	0.504	0.368	0.387	0.407	0.437	0.404
F1	SFGmed-R	0.041	0.009	0.020	0.029	0.004	0.011
F1	SFGmed-L	0.176	0.173	0.181	0.113	0.059	0.078
F2	SFGdor-R	0.304	0.513	0.510	0.517	0.684	0.572
F2	MFG-R	0.339	0.243	0.313	0.282	0.190	0.346

SD distance = 25 mm		Circumferential SD pair			Vertical SD pair		
10/10 Points	AAL regions	0 year	1 year	2 year	0 year	1 year	2 year
F2	SFGmed-R	0.291	0.230	0.163	0.146	0.117	0.075
F2	SFGmed-L	0.042	0.012	0.012	0.027	0.007	0.006
F4	SFGdor-R	0.100	0.052	0.037	0.080	0.036	0.029
F4	MFG-R	0.704	0.896	0.871	0.810	0.900	0.891
F4	IFGoperc-R	0.013	0.002	0.002	0.004	0.001	0.001
F4	IFGtriang-R	0.161	0.047	0.088	0.084	0.060	0.077
F6	SFGdor-R	0.011	0.001	0.001	0.009	0.001	0.001
F6	MFG-R	0.354	0.225	0.241	0.400	0.184	0.197
F6	ORBmid-R	0.030	0.011	0.018	0.047	0.015	0.016
F6	IFGoperc-R	0.046	0.025	0.007	0.016	0.010	0.005
F6	IFGtriang-R	0.484	0.711	0.714	0.420	0.723	0.745
F6	ORBinf-R	0.047	0.020	0.014	0.093	0.063	0.031
AF3	SFGdor-L	0.304	0.395	0.413	0.302	0.311	0.383
AF3	ORBsup-L	0.017	0.011	0.015	0.034	0.024	0.045
AF3	MFG-L	0.497	0.537	0.467	0.505	0.605	0.471
AF3	ORBmid-L	0.034	0.015	0.024	0.076	0.038	0.059
AF3	IFGtriang-L	0.013	0.002	0.003	0.007	0.001	0.002
AF3	SFGmed-R	0.019	0.002	0.004	0.012	0.001	0.003
AF3	SFGmed-L	0.101	0.036	0.070	0.050	0.018	0.033
AF4	SFGdor-R	0.350	0.451	0.377	0.432	0.546	0.345
AF4	ORBsup-R	0.011	0.005	0.009	0.022	0.012	0.018
AF4	MFG-R	0.412	0.437	0.535	0.384	0.366	0.566
AF4	ORBmid-R	0.020	0.008	0.018	0.043	0.020	0.037
AF4	IFGtriang-R	0.017	0.004	0.006	0.009	0.001	0.004
AF4	SFGmed-R	0.154	0.090	0.049	0.080	0.049	0.025
AF4	SFGmed-L	0.018	0.003	0.002	0.011	0.002	0.002
CP5	PoCG-L	0.040	0.012	0.011	0.020	0.007	0.007
CP5	SMG-L	0.233	0.501	0.450	0.389	0.498	0.412
CP5	ANG-L	0.017	0.056	0.044	0.005	0.011	0.011
CP5	STG-L	0.612	0.397	0.450	0.353	0.409	0.462
CP5	MTG-L	0.091	0.028	0.037	0.227	0.070	0.103
CP3	PreCG-L	0.010	0.004	0.007	0.008	0.005	0.007

SD distance = 25 mm		Circumferential SD pair			Vertical SD pair		
10/10 Points	AAL regions	0 year	1 year	2 year	0 year	1 year	2 year
CP3	PoCG-L	0.209	0.205	0.121	0.235	0.215	0.134
CP3	SPG-L	0.021	0.021	0.024	0.011	0.017	0.022
CP3	IPL-L	0.364	0.529	0.539	0.209	0.393	0.532
CP3	SMG-L	0.379	0.237	0.297	0.508	0.363	0.289
CP3	ANG-L	0.013	0.003	0.011	0.016	0.007	0.015
CP1	PreCG-L	0.139	0.083	0.095	0.245	0.126	0.121
CP1	PoCG-L	0.467	0.567	0.546	0.343	0.504	0.512
CP1	SPG-L	0.313	0.316	0.316	0.236	0.309	0.296
CP1	IPL-L	0.048	0.025	0.031	0.133	0.048	0.058
CP2	PreCG-R	0.124	0.051	0.068	0.125	0.057	0.112
CP2	SFGdor-R	0.013	0.002	0.004	0.020	0.004	0.008
CP2	PoCG-R	0.406	0.514	0.537	0.400	0.473	0.519
CP2	SPG-R	0.299	0.326	0.302	0.255	0.349	0.256
CP2	IPL-R	0.024	0.021	0.028	0.073	0.042	0.045
CP2	PCUN-R	0.086	0.061	0.033	0.034	0.028	0.016
CP2	PCL-R	0.029	0.021	0.023	0.066	0.043	0.038
CP4	PreCG-R	0.018	0.002	0.010	0.020	0.003	0.011
CP4	PoCG-R	0.241	0.196	0.245	0.267	0.262	0.310
CP4	SPG-R	0.028	0.020	0.013	0.020	0.020	0.011
CP4	IPL-R	0.592	0.747	0.707	0.536	0.684	0.634
CP4	SMG-R	0.064	0.012	0.016	0.103	0.012	0.025
CP4	ANG-R	0.052	0.022	0.009	0.049	0.019	0.008
CP6	PoCG-R	0.045	0.011	0.026	0.025	0.005	0.014
CP6	IPL-R	0.046	0.074	0.083	0.105	0.171	0.106
CP6	SMG-R	0.389	0.531	0.694	0.402	0.441	0.609
CP6	ANG-R	0.108	0.072	0.034	0.016	0.022	0.017
CP6	STG-R	0.235	0.265	0.139	0.189	0.276	0.211
CP6	MTG-R	0.171	0.047	0.023	0.258	0.084	0.042
P5	MOG-L	0.071	0.026	0.031	0.012	0.018	0.027
P5	IPL-L	0.017	0.049	0.038	0.097	0.170	0.110
P5	SMG-L	0.117	0.090	0.046	0.073	0.034	0.022
P5	ANG-L	0.292	0.610	0.613	0.324	0.473	0.510

SD distance = 25 mm		Circumferential SD pair			Vertical SD pair		
10/10 Points	AAL regions	0 year	1 year	2 year	0 year	1 year	2 year
P5	STG-L	0.247	0.112	0.106	0.113	0.054	0.030
P5	MTG-L	0.255	0.113	0.165	0.380	0.252	0.300
P3	SPG-L	0.075	0.090	0.063	0.071	0.085	0.059
P3	IPL-L	0.435	0.508	0.403	0.293	0.314	0.331
P3	SMG-L	0.050	0.008	0.005	0.011	0.004	0.003
P3	ANG-L	0.419	0.388	0.523	0.579	0.588	0.599
P1	PoCG-L	0.033	0.011	0.009	0.034	0.014	0.010
P1	SPG-L	0.678	0.853	0.814	0.698	0.824	0.825
P1	IPL-L	0.127	0.055	0.074	0.040	0.036	0.038
P1	ANG-L	0.097	0.057	0.074	0.180	0.114	0.105
P1	PCUN-R	0.013	0.004	0.006	0.007	0.002	0.004
P1	PCUN-L	0.036	0.017	0.020	0.017	0.007	0.013
P2	SOG-R	0.012	0.003	0.001	0.038	0.006	0.003
P2	PoCG-R	0.030	0.003	0.010	0.023	0.003	0.009
P2	SPG-R	0.543	0.633	0.690	0.579	0.688	0.761
P2	SPG-L	0.011	0.003	0.001	0.008	0.002	0.001
P2	IPL-R	0.060	0.019	0.030	0.064	0.022	0.044
P2	ANG-R	0.048	0.015	0.017	0.031	0.010	0.023
P2	PCUN-R	0.259	0.309	0.240	0.224	0.258	0.153
P2	PCUN-L	0.021	0.013	0.008	0.014	0.008	0.005
P4	SOG-R	0.018	0.003	0.001	0.008	0.002	0.001
P4	MOG-R	0.034	0.006	0.008	0.192	0.026	0.017
P4	SPG-R	0.142	0.256	0.153	0.143	0.232	0.175
P4	IPL-R	0.365	0.278	0.350	0.145	0.162	0.274
P4	ANG-R	0.419	0.450	0.479	0.495	0.572	0.525
P6	MOG-R	0.216	0.100	0.038	0.093	0.068	0.024
P6	IPL-R	0.043	0.060	0.084	0.110	0.073	0.119
P6	SMG-R	0.127	0.070	0.111	0.040	0.019	0.051
P6	ANG-R	0.454	0.712	0.722	0.583	0.750	0.722
P6	STG-R	0.028	0.005	0.005	0.008	0.002	0.004
P6	MTG-R	0.128	0.049	0.039	0.158	0.085	0.080
PO3	CUN-L	0.026	0.017	0.015	0.011	0.006	0.008

SD distance = 25 mm		Circumferential SD pair			Vertical SD pair		
10/10 Points	AAL regions	0 year	1 year	2 year	0 year	1 year	2 year
PO3	SOG-L	0.115	0.234	0.198	0.114	0.114	0.141
PO3	MOG-L	0.247	0.167	0.220	0.436	0.310	0.331
PO3	SPG-L	0.073	0.094	0.071	0.135	0.106	0.108
PO3	ANG-L	0.519	0.483	0.490	0.292	0.459	0.406
PO4	CUN-R	0.032	0.022	0.011	0.030	0.018	0.009
PO4	SOG-R	0.240	0.388	0.310	0.386	0.397	0.263
PO4	MOG-R	0.378	0.246	0.262	0.235	0.241	0.300
PO4	SPG-R	0.051	0.076	0.079	0.142	0.170	0.132
PO4	IPL-R	0.155	0.214	0.257	0.129	0.142	0.252
PO4	ANG-R	0.105	0.033	0.065	0.029	0.014	0.026
PO4	PCUN-R	0.023	0.014	0.013	0.034	0.014	0.014

Table S7 Normalized PPL of corresponding brain regions ($L_{norm,M}$) for the circumferential and vertical SD pairs set at 10/10 fiducial points at 30-mm SD distance from 0-yo, 1-yo, and 2-yo.

SD distance = 30 mm		Circumferential SD pair			Vertical SD pair		
10/10 Points	AAL regions	0 year	1 year	2 year	0 year	1 year	2 year
Cz	PreCG-R	0.053	0.015	0.013	0.018	0.006	0.008
Cz	PreCG-L	0.092	0.043	0.028	0.080	0.045	0.022
Cz	SFGdor-R	0.222	0.127	0.130	0.060	0.029	0.050
Cz	SFGdor-L	0.240	0.254	0.207	0.126	0.105	0.069
Cz	SMA-R	0.111	0.275	0.314	0.163	0.270	0.402
Cz	SMA-L	0.105	0.211	0.252	0.235	0.380	0.322
Cz	PoCG-R	0.021	0.003	0.002	0.014	0.002	0.002
Cz	PoCG-L	0.034	0.006	0.004	0.061	0.013	0.005
Cz	SPG-L	0.013	0.000	0.000	0.016	0.001	0.000
Cz	PCL-R	0.066	0.048	0.031	0.092	0.088	0.080
Cz	PCL-L	0.020	0.011	0.009	0.089	0.051	0.026
Fpz	SFGdor-R	0.099	0.059	0.058	0.037	0.021	0.036
Fpz	SFGdor-L	0.120	0.092	0.024	0.066	0.057	0.017
Fpz	ORBsup-R	0.067	0.067	0.112	0.034	0.035	0.058
Fpz	ORBsup-L	0.108	0.156	0.119	0.133	0.111	0.071
Fpz	MFG-R	0.011	0.002	0.004	0.004	0.001	0.002
Fpz	MFG-L	0.018	0.003	0.001	0.010	0.002	0.001
Fpz	ORBmid-R	0.022	0.008	0.018	0.009	0.003	0.008
Fpz	ORBmid-L	0.039	0.021	0.020	0.030	0.016	0.010
Fpz	SFGmed-R	0.154	0.121	0.083	0.194	0.131	0.157
Fpz	SFGmed-L	0.145	0.135	0.080	0.199	0.249	0.122
Fpz	ORBmed-R	0.105	0.181	0.221	0.103	0.149	0.248
Fpz	ORBmed-L	0.091	0.135	0.224	0.123	0.172	0.198
Fpz	REC-R	0.013	0.015	0.029	0.032	0.038	0.055
AFz	SFGdor-R	0.219	0.115	0.181	0.076	0.030	0.071
AFz	SFGdor-L	0.205	0.249	0.141	0.110	0.095	0.054
AFz	MFG-R	0.030	0.004	0.010	0.011	0.001	0.004
AFz	MFG-L	0.077	0.046	0.014	0.025	0.010	0.006
AFz	SFGmed-R	0.252	0.333	0.363	0.386	0.358	0.509
AFz	SFGmed-L	0.187	0.246	0.282	0.342	0.482	0.332

SD distance = 30 mm		Circumferential SD pair			Vertical SD pair		
10/10 Points	AAL regions	0 year	1 year	2 year	0 year	1 year	2 year
Fz	SFGdor-R	0.177	0.127	0.186	0.066	0.036	0.090
Fz	SFGdor-L	0.352	0.332	0.178	0.100	0.091	0.058
Fz	MFG-R	0.024	0.003	0.011	0.010	0.001	0.007
Fz	MFG-L	0.064	0.018	0.013	0.021	0.006	0.005
Fz	SMA-R	0.020	0.003	0.001	0.028	0.004	0.001
Fz	SMA-L	0.016	0.004	0.001	0.093	0.004	0.001
Fz	SFGmed-R	0.179	0.261	0.333	0.292	0.381	0.528
Fz	SFGmed-L	0.165	0.252	0.279	0.386	0.473	0.310
FCz	PreCG-R	0.068	0.000	0.000	0.004	0.000	0.000
FCz	PreCG-L	0.012	0.000	0.000	0.011	0.000	0.000
FCz	SFGdor-R	0.219	0.114	0.153	0.089	0.031	0.067
FCz	SFGdor-L	0.267	0.250	0.143	0.136	0.087	0.050
FCz	MFG-R	0.030	0.007	0.016	0.013	0.002	0.008
FCz	MFG-L	0.026	0.012	0.007	0.012	0.004	0.003
FCz	SMA-R	0.123	0.156	0.130	0.187	0.176	0.234
FCz	SMA-L	0.116	0.186	0.104	0.231	0.313	0.158
FCz	SFGmed-R	0.059	0.147	0.213	0.142	0.158	0.272
FCz	SFGmed-L	0.070	0.128	0.233	0.157	0.226	0.208
CPz	PreCG-R	0.025	0.005	0.006	0.014	0.003	0.005
CPz	PreCG-L	0.070	0.028	0.042	0.076	0.041	0.032
CPz	SFGdor-R	0.012	0.002	0.002	0.016	0.002	0.003
CPz	SFGdor-L	0.012	0.002	0.004	0.030	0.006	0.007
CPz	SMA-R	0.012	0.005	0.008	0.032	0.016	0.024
CPz	SMA-L	0.015	0.009	0.013	0.053	0.046	0.042
CPz	PoCG-R	0.153	0.056	0.058	0.030	0.011	0.018
CPz	PoCG-L	0.246	0.296	0.260	0.145	0.153	0.111
CPz	SPG-R	0.049	0.036	0.022	0.019	0.011	0.011
CPz	SPG-L	0.094	0.086	0.045	0.147	0.076	0.044
CPz	PCUN-R	0.113	0.187	0.161	0.135	0.166	0.188
CPz	PCUN-L	0.035	0.071	0.050	0.109	0.159	0.121
CPz	PCL-R	0.107	0.138	0.212	0.106	0.161	0.242
CPz	PCL-L	0.055	0.079	0.116	0.085	0.149	0.152

SD distance = 30 mm		Circumferential SD pair			Vertical SD pair		
10/10 Points	AAL regions	0 year	1 year	2 year	0 year	1 year	2 year
Pz	PoCG-R	0.013	0.001	0.002	0.007	0.001	0.001
Pz	PoCG-L	0.038	0.010	0.009	0.072	0.023	0.014
Pz	SPG-R	0.160	0.051	0.065	0.030	0.013	0.019
Pz	SPG-L	0.403	0.415	0.286	0.321	0.322	0.153
Pz	PCUN-R	0.248	0.343	0.417	0.254	0.270	0.445
Pz	PCUN-L	0.098	0.171	0.212	0.244	0.352	0.352
POz	CUN-R	0.079	0.062	0.080	0.173	0.125	0.155
POz	CUN-L	0.092	0.161	0.139	0.153	0.240	0.152
POz	SOG-R	0.178	0.128	0.169	0.062	0.053	0.076
POz	SOG-L	0.129	0.087	0.043	0.089	0.049	0.019
POz	MOG-L	0.029	0.006	0.004	0.018	0.004	0.002
POz	SPG-R	0.036	0.064	0.058	0.015	0.017	0.021
POz	SPG-L	0.258	0.275	0.242	0.196	0.193	0.148
POz	ANG-L	0.035	0.007	0.005	0.011	0.003	0.001
POz	PCUN-R	0.100	0.143	0.182	0.149	0.166	0.267
POz	PCUN-L	0.046	0.062	0.071	0.126	0.149	0.156
Oz	CAL-R	0.029	0.022	0.029	0.096	0.065	0.090
Oz	CUN-R	0.206	0.263	0.370	0.208	0.294	0.466
Oz	CUN-L	0.108	0.128	0.149	0.213	0.295	0.221
Oz	SOG-R	0.159	0.120	0.153	0.043	0.047	0.067
Oz	SOG-L	0.304	0.374	0.252	0.270	0.238	0.109
Oz	MOG-R	0.014	0.004	0.009	0.005	0.003	0.005
Oz	MOG-L	0.151	0.076	0.023	0.063	0.025	0.008
T3	STG-L	0.035	0.092	0.066	0.150	0.321	0.258
T3	TPOsup-L	0.036	0.016	0.015	0.033	0.015	0.014
T3	MTG-L	0.416	0.787	0.773	0.415	0.531	0.572
T3	TPOmid-L	0.083	0.029	0.031	0.037	0.009	0.010
T3	ITG-L	0.418	0.074	0.113	0.346	0.117	0.139
C5	PreCG-L	0.036	0.074	0.057	0.048	0.043	0.041
C5	IFGoperc-L	0.038	0.013	0.013	0.012	0.004	0.005
C5	IFGtriang-L	0.019	0.002	0.003	0.006	0.001	0.001
C5	ROL-L	0.075	0.151	0.141	0.041	0.054	0.036

SD distance = 30 mm		Circumferential SD pair			Vertical SD pair		
10/10 Points	AAL regions	0 year	1 year	2 year	0 year	1 year	2 year
C5	PoCG-L	0.126	0.290	0.273	0.422	0.414	0.428
C5	SMG-L	0.075	0.130	0.102	0.051	0.078	0.098
C5	STG-L	0.514	0.324	0.382	0.269	0.375	0.352
C5	TPOsup-L	0.037	0.007	0.012	0.020	0.005	0.004
C5	MTG-L	0.071	0.009	0.015	0.121	0.025	0.034
C3	PreCG-L	0.225	0.344	0.346	0.205	0.278	0.267
C3	MFG-L	0.081	0.095	0.075	0.087	0.085	0.136
C3	IFGoperc-L	0.023	0.006	0.006	0.006	0.002	0.002
C3	PoCG-L	0.328	0.387	0.388	0.467	0.544	0.447
C3	IPL-L	0.019	0.010	0.012	0.006	0.002	0.004
C3	SMG-L	0.310	0.154	0.170	0.201	0.086	0.140
C1	PreCG-L	0.339	0.390	0.399	0.311	0.420	0.402
C1	SFGdor-L	0.200	0.244	0.218	0.260	0.355	0.320
C1	MFG-L	0.244	0.274	0.296	0.178	0.116	0.183
C1	SMA-L	0.014	0.012	0.007	0.029	0.032	0.014
C1	PoCG-L	0.174	0.075	0.074	0.170	0.071	0.074
C2	PreCG-R	0.326	0.457	0.404	0.325	0.463	0.427
C2	SFGdor-R	0.168	0.133	0.218	0.280	0.274	0.316
C2	MFG-R	0.219	0.228	0.271	0.070	0.065	0.137
C2	SMA-R	0.024	0.011	0.020	0.062	0.034	0.045
C2	PoCG-R	0.213	0.157	0.073	0.199	0.149	0.062
C2	SPG-R	0.010	0.003	0.001	0.005	0.001	0.001
C2	PCL-R	0.019	0.008	0.010	0.026	0.011	0.008
C4	PreCG-R	0.227	0.278	0.384	0.235	0.228	0.365
C4	MFG-R	0.059	0.024	0.048	0.034	0.015	0.037
C4	PoCG-R	0.314	0.444	0.427	0.414	0.605	0.482
C4	IPL-R	0.224	0.187	0.093	0.068	0.050	0.036
C4	SMG-R	0.152	0.065	0.042	0.219	0.099	0.076
C6	PreCG-R	0.018	0.015	0.036	0.044	0.012	0.046
C6	IFGoperc-R	0.039	0.004	0.020	0.015	0.001	0.007
C6	IFGtriang-R	0.017	0.001	0.003	0.010	0.000	0.002
C6	ROL-R	0.061	0.067	0.052	0.030	0.015	0.024

SD distance = 30 mm		Circumferential SD pair			Vertical SD pair		
10/10 Points	AAL regions	0 year	1 year	2 year	0 year	1 year	2 year
C6	PoCG-R	0.194	0.378	0.442	0.354	0.426	0.488
C6	SMG-R	0.091	0.129	0.168	0.064	0.094	0.075
C6	STG-R	0.336	0.351	0.241	0.173	0.279	0.267
C6	TPOsup-R	0.013	0.001	0.001	0.008	0.000	0.001
C6	MTG-R	0.215	0.051	0.032	0.283	0.169	0.085
T4	STG-R	0.022	0.022	0.046	0.060	0.070	0.094
T4	TPOsup-R	0.017	0.003	0.009	0.017	0.002	0.005
T4	MTG-R	0.562	0.746	0.819	0.533	0.695	0.682
T4	TPOmid-R	0.020	0.002	0.003	0.014	0.001	0.002
T4	ITG-R	0.366	0.224	0.117	0.352	0.227	0.205
FT7	IFGoperc-L	0.015	0.016	0.013	0.054	0.048	0.031
FT7	IFGtriang-L	0.048	0.028	0.029	0.056	0.029	0.028
FT7	ORBinf-L	0.185	0.113	0.100	0.066	0.028	0.028
FT7	ROL-L	0.009	0.015	0.007	0.027	0.067	0.019
FT7	STG-L	0.071	0.147	0.101	0.052	0.101	0.071
FT7	TPOsup-L	0.231	0.339	0.334	0.339	0.366	0.369
FT7	MTG-L	0.256	0.211	0.267	0.110	0.147	0.202
FT7	TPOmid-L	0.119	0.113	0.135	0.210	0.182	0.219
FT7	ITG-L	0.044	0.011	0.011	0.058	0.020	0.029
F7	MFG-L	0.038	0.014	0.009	0.044	0.011	0.011
F7	ORBmid-L	0.177	0.050	0.045	0.067	0.012	0.015
F7	IFGoperc-L	0.015	0.011	0.006	0.010	0.013	0.005
F7	IFGtriang-L	0.147	0.144	0.102	0.358	0.391	0.351
F7	ORBinf-L	0.418	0.608	0.599	0.426	0.472	0.509
F7	TPOsup-L	0.153	0.151	0.208	0.064	0.084	0.089
F7	TPOmid-L	0.021	0.013	0.023	0.015	0.010	0.015
AF7	SFGdor-L	0.047	0.013	0.012	0.040	0.007	0.011
AF7	ORBsup-L	0.046	0.027	0.033	0.022	0.008	0.015
AF7	MFG-L	0.165	0.131	0.078	0.345	0.289	0.270
AF7	ORBmid-L	0.380	0.463	0.508	0.398	0.411	0.487
AF7	IFGtriang-L	0.105	0.063	0.043	0.059	0.077	0.052
AF7	ORBinf-L	0.236	0.301	0.318	0.125	0.206	0.163

SD distance = 30 mm		Circumferential SD pair			Vertical SD pair		
10/10 Points	AAL regions	0 year	1 year	2 year	0 year	1 year	2 year
Fp1	SFGdor-L	0.166	0.163	0.085	0.276	0.294	0.243
Fp1	ORBsup-L	0.137	0.252	0.255	0.165	0.245	0.290
Fp1	MFG-L	0.154	0.125	0.052	0.133	0.119	0.062
Fp1	ORBmid-L	0.265	0.341	0.391	0.261	0.275	0.283
Fp1	ORBinf-L	0.017	0.006	0.006	0.011	0.003	0.003
Fp1	SFGmed-R	0.035	0.003	0.005	0.017	0.002	0.004
Fp1	SFGmed-L	0.107	0.048	0.043	0.069	0.029	0.042
Fp1	ORBmed-R	0.023	0.004	0.014	0.011	0.002	0.007
Fp1	ORBmed-L	0.071	0.055	0.140	0.039	0.028	0.059
Fp2	SFGdor-R	0.199	0.190	0.141	0.332	0.367	0.283
Fp2	ORBsup-R	0.105	0.192	0.221	0.144	0.236	0.222
Fp2	ORBsup-L	0.011	0.001	0.002	0.006	0.001	0.001
Fp2	MFG-R	0.165	0.103	0.088	0.131	0.070	0.144
Fp2	ORBmid-R	0.172	0.311	0.349	0.149	0.164	0.238
Fp2	ORBinf-R	0.021	0.005	0.014	0.013	0.002	0.007
Fp2	SFGmed-R	0.139	0.060	0.037	0.111	0.063	0.032
Fp2	SFGmed-L	0.041	0.004	0.004	0.021	0.004	0.003
Fp2	ORBmed-R	0.088	0.119	0.115	0.056	0.073	0.049
Fp2	ORBmed-L	0.029	0.006	0.011	0.013	0.004	0.006
Fp2	REC-R	0.009	0.007	0.014	0.012	0.014	0.014
AF8	SFGdor-R	0.092	0.029	0.022	0.062	0.013	0.014
AF8	ORBsup-R	0.048	0.024	0.028	0.027	0.009	0.012
AF8	MFG-R	0.202	0.177	0.115	0.381	0.278	0.301
AF8	ORBmid-R	0.287	0.476	0.492	0.306	0.453	0.418
AF8	IFGtriang-R	0.140	0.099	0.096	0.074	0.080	0.106
AF8	ORBinf-R	0.202	0.191	0.237	0.132	0.163	0.145
F8	MFG-R	0.058	0.008	0.013	0.054	0.004	0.013
F8	ORBmid-R	0.148	0.044	0.049	0.058	0.006	0.020
F8	IFGoperc-R	0.023	0.012	0.013	0.015	0.020	0.010
F8	IFGtriang-R	0.192	0.181	0.238	0.349	0.364	0.460
F8	ORBinf-R	0.405	0.540	0.446	0.425	0.446	0.337
F8	STG-R	0.018	0.015	0.009	0.006	0.007	0.004

SD distance = 30 mm		Circumferential SD pair			Vertical SD pair		
10/10 Points	AAL regions	0 year	1 year	2 year	0 year	1 year	2 year
F8	TPOsup-R	0.106	0.161	0.195	0.061	0.122	0.125
F8	MTG-R	0.014	0.017	0.015	0.006	0.008	0.007
F8	TPOmid-R	0.010	0.013	0.015	0.011	0.015	0.018
FT8	IFGoperc-R	0.020	0.008	0.017	0.067	0.025	0.043
FT8	IFGtriang-R	0.058	0.015	0.037	0.066	0.020	0.034
FT8	ORBinf-R	0.211	0.043	0.057	0.075	0.017	0.021
FT8	ROL-R	0.017	0.012	0.017	0.040	0.058	0.048
FT8	STG-R	0.081	0.102	0.104	0.089	0.200	0.144
FT8	TPOsup-R	0.173	0.207	0.259	0.225	0.173	0.242
FT8	MTG-R	0.339	0.542	0.446	0.241	0.412	0.380
FT8	TPOmid-R	0.046	0.044	0.043	0.113	0.048	0.061
FT8	ITG-R	0.034	0.022	0.014	0.059	0.034	0.019
TP7	STG-L	0.061	0.075	0.043	0.254	0.217	0.102
TP7	MTG-L	0.542	0.827	0.812	0.438	0.557	0.610
TP7	ITG-L	0.390	0.096	0.144	0.297	0.223	0.285
T5	MOG-L	0.041	0.015	0.017	0.004	0.009	0.007
T5	IOG-L	0.017	0.003	0.006	0.006	0.003	0.005
T5	STG-L	0.031	0.030	0.017	0.134	0.116	0.040
T5	MTG-L	0.679	0.861	0.838	0.581	0.687	0.708
T5	ITG-L	0.225	0.081	0.117	0.252	0.144	0.225
PO7	MOG-L	0.410	0.482	0.535	0.297	0.505	0.577
PO7	IOG-L	0.017	0.007	0.012	0.143	0.021	0.039
PO7	ANG-L	0.027	0.047	0.013	0.121	0.145	0.049
PO7	MTG-L	0.510	0.451	0.430	0.359	0.317	0.324
PO7	ITG-L	0.020	0.008	0.007	0.066	0.009	0.007
O1	CUN-R	0.020	0.004	0.013	0.007	0.002	0.006
O1	CUN-L	0.040	0.014	0.024	0.022	0.012	0.015
O1	SOG-L	0.212	0.264	0.278	0.203	0.177	0.207
O1	MOG-L	0.649	0.690	0.666	0.639	0.765	0.743
O1	ANG-L	0.018	0.014	0.005	0.033	0.024	0.012
O1	MTG-L	0.038	0.007	0.004	0.002	0.003	0.002
O2	CAL-R	0.039	0.012	0.004	0.059	0.024	0.006

SD distance = 30 mm		Circumferential SD pair			Vertical SD pair		
10/10 Points	AAL regions	0 year	1 year	2 year	0 year	1 year	2 year
O2	CUN-R	0.174	0.199	0.106	0.065	0.093	0.051
O2	CUN-L	0.024	0.014	0.006	0.009	0.008	0.004
O2	SOG-R	0.297	0.477	0.438	0.412	0.584	0.487
O2	MOG-R	0.436	0.284	0.435	0.409	0.274	0.438
PO8	SOG-R	0.061	0.060	0.019	0.014	0.022	0.010
PO8	MOG-R	0.718	0.819	0.771	0.821	0.847	0.816
PO8	IOG-R	0.030	0.012	0.006	0.042	0.017	0.008
PO8	ANG-R	0.043	0.050	0.063	0.088	0.084	0.110
PO8	MTG-R	0.135	0.053	0.137	0.019	0.020	0.051
T6	MOG-R	0.230	0.233	0.147	0.082	0.160	0.108
T6	IOG-R	0.067	0.048	0.021	0.235	0.158	0.080
T6	ANG-R	0.022	0.047	0.040	0.096	0.104	0.098
T6	STG-R	0.017	0.016	0.029	0.027	0.010	0.023
T6	MTG-R	0.537	0.614	0.728	0.359	0.505	0.610
T6	ITG-R	0.119	0.033	0.025	0.153	0.045	0.050
TP8	STG-R	0.025	0.030	0.064	0.072	0.120	0.128
TP8	MTG-R	0.645	0.821	0.863	0.533	0.664	0.739
TP8	ITG-R	0.313	0.136	0.062	0.376	0.201	0.119
FC5	PreCG-L	0.077	0.136	0.123	0.170	0.178	0.128
FC5	MFG-L	0.037	0.012	0.011	0.030	0.020	0.014
FC5	IFGoperc-L	0.103	0.172	0.183	0.248	0.381	0.351
FC5	IFGtriang-L	0.371	0.376	0.364	0.220	0.202	0.262
FC5	ORBinf-L	0.047	0.008	0.009	0.041	0.015	0.017
FC5	ROL-L	0.091	0.146	0.150	0.053	0.091	0.079
FC5	PoCG-L	0.134	0.105	0.086	0.048	0.024	0.020
FC5	STG-L	0.065	0.020	0.030	0.032	0.014	0.018
FC5	TPOsup-L	0.055	0.023	0.040	0.129	0.071	0.106
FC3	PreCG-L	0.258	0.268	0.236	0.121	0.121	0.085
FC3	MFG-L	0.525	0.607	0.599	0.533	0.560	0.541
FC3	IFGoperc-L	0.075	0.071	0.094	0.131	0.156	0.176
FC3	IFGtriang-L	0.066	0.041	0.051	0.176	0.153	0.186
FC3	PoCG-L	0.062	0.006	0.014	0.018	0.004	0.005

SD distance = 30 mm		Circumferential SD pair			Vertical SD pair		
10/10 Points	AAL regions	0 year	1 year	2 year	0 year	1 year	2 year
FC1	PreCG-L	0.046	0.011	0.007	0.049	0.003	0.004
FC1	SFGdor-L	0.305	0.462	0.430	0.323	0.470	0.423
FC1	MFG-L	0.539	0.484	0.494	0.535	0.474	0.510
FC1	SMA-L	0.024	0.017	0.015	0.047	0.037	0.033
FC1	SFGmed-L	0.059	0.023	0.048	0.021	0.012	0.025
FC2	PreCG-R	0.091	0.011	0.011	0.028	0.005	0.006
FC2	SFGdor-R	0.293	0.411	0.320	0.301	0.348	0.305
FC2	MFG-R	0.408	0.483	0.603	0.477	0.549	0.634
FC2	SMA-R	0.060	0.026	0.015	0.110	0.073	0.029
FC2	SMA-L	0.011	0.002	0.002	0.015	0.002	0.002
FC2	SFGmed-R	0.079	0.064	0.045	0.028	0.021	0.022
FC2	SFGmed-L	0.013	0.002	0.003	0.008	0.001	0.002
FC2	PoCG-R	0.033	0.000	0.000	0.003	0.000	0.000
FC4	PreCG-R	0.231	0.290	0.219	0.187	0.195	0.113
FC4	SFGdor-R	0.017	0.002	0.005	0.015	0.002	0.004
FC4	MFG-R	0.578	0.613	0.692	0.542	0.641	0.667
FC4	IFGoperc-R	0.033	0.026	0.027	0.077	0.053	0.061
FC4	IFGtriang-R	0.052	0.043	0.044	0.127	0.096	0.146
FC4	PoCG-R	0.077	0.026	0.013	0.039	0.013	0.008
FC6	PreCG-R	0.056	0.094	0.088	0.164	0.247	0.124
FC6	MFG-R	0.051	0.016	0.024	0.059	0.026	0.038
FC6	IFGoperc-R	0.113	0.143	0.158	0.176	0.179	0.259
FC6	IFGtriang-R	0.345	0.315	0.449	0.206	0.136	0.357
FC6	ORBinf-R	0.025	0.003	0.003	0.026	0.004	0.005
FC6	ROL-R	0.057	0.077	0.060	0.057	0.117	0.070
FC6	PoCG-R	0.208	0.295	0.168	0.111	0.144	0.068
FC6	STG-R	0.078	0.041	0.031	0.079	0.088	0.037
FC6	TPOsup-R	0.027	0.010	0.013	0.071	0.046	0.037
FC6	MTG-R	0.025	0.005	0.004	0.038	0.012	0.005
F5	SFGdor-L	0.011	0.001	0.002	0.006	0.000	0.001
F5	MFG-L	0.291	0.332	0.332	0.369	0.270	0.273
F5	ORBmid-L	0.048	0.013	0.017	0.058	0.013	0.020

SD distance = 30 mm		Circumferential SD pair			Vertical SD pair		
10/10 Points	AAL regions	0 year	1 year	2 year	0 year	1 year	2 year
F5	IFGoperc-L	0.051	0.031	0.014	0.020	0.011	0.007
F5	IFGtriang-L	0.480	0.578	0.580	0.381	0.566	0.523
F5	ORBinf-L	0.081	0.038	0.048	0.150	0.135	0.171
F5	TPOsup-L	0.014	0.003	0.003	0.007	0.002	0.003
F3	SFGdor-L	0.065	0.019	0.035	0.054	0.023	0.025
F3	MFG-L	0.664	0.854	0.852	0.819	0.919	0.904
F3	IFGoperc-L	0.035	0.007	0.006	0.008	0.002	0.003
F3	IFGtriang-L	0.207	0.115	0.101	0.084	0.051	0.063
F1	SFGdor-L	0.211	0.405	0.373	0.403	0.469	0.478
F1	MFG-L	0.546	0.368	0.395	0.387	0.457	0.421
F1	SFGmed-R	0.056	0.015	0.027	0.040	0.006	0.013
F1	SFGmed-L	0.166	0.209	0.201	0.145	0.066	0.085
F2	SFGdor-R	0.252	0.440	0.448	0.491	0.612	0.544
F2	SFGdor-L	0.016	0.001	0.002	0.008	0.001	0.001
F2	MFG-R	0.342	0.276	0.330	0.274	0.243	0.358
F2	SFGmed-R	0.271	0.262	0.202	0.167	0.133	0.088
F2	SFGmed-L	0.097	0.019	0.017	0.034	0.011	0.008
F4	SFGdor-R	0.137	0.080	0.056	0.097	0.050	0.038
F4	MFG-R	0.633	0.845	0.840	0.775	0.877	0.862
F4	IFGoperc-R	0.028	0.004	0.004	0.005	0.001	0.001
F4	IFGtriang-R	0.170	0.066	0.096	0.092	0.068	0.095
F4	SFGmed-R	0.014	0.003	0.003	0.012	0.002	0.002
F6	SFGdor-R	0.019	0.001	0.002	0.011	0.001	0.001
F6	MFG-R	0.402	0.248	0.287	0.402	0.211	0.220
F6	ORBmid-R	0.033	0.013	0.021	0.055	0.022	0.021
F6	IFGoperc-R	0.063	0.040	0.013	0.019	0.011	0.006
F6	IFGtriang-R	0.407	0.664	0.654	0.368	0.649	0.703
F6	ORBinf-R	0.044	0.022	0.015	0.125	0.099	0.042
AF3	SFGdor-L	0.256	0.355	0.372	0.292	0.297	0.362
AF3	ORBsup-L	0.015	0.012	0.016	0.036	0.033	0.058
AF3	MFG-L	0.495	0.553	0.475	0.489	0.594	0.455
AF3	ORBmid-L	0.029	0.018	0.025	0.092	0.049	0.075

SD distance = 30 mm		Circumferential SD pair			Vertical SD pair		
10/10 Points	AAL regions	0 year	1 year	2 year	0 year	1 year	2 year
AF3	IFGtriang-L	0.017	0.004	0.004	0.008	0.002	0.002
AF3	SFGmed-R	0.033	0.002	0.006	0.013	0.002	0.004
AF3	SFGmed-L	0.137	0.053	0.096	0.053	0.021	0.039
AF4	SFGdor-R	0.296	0.413	0.371	0.423	0.459	0.360
AF4	ORBsup-R	0.012	0.005	0.008	0.029	0.016	0.027
AF4	MFG-R	0.394	0.435	0.517	0.348	0.442	0.512
AF4	ORBmid-R	0.022	0.008	0.016	0.059	0.029	0.056
AF4	IFGtriang-R	0.026	0.005	0.010	0.010	0.002	0.004
AF4	SFGmed-R	0.198	0.125	0.070	0.091	0.045	0.033
AF4	SFGmed-L	0.027	0.005	0.004	0.014	0.002	0.002
CP5	PoCG-L	0.081	0.023	0.018	0.030	0.011	0.010
CP5	SMG-L	0.258	0.460	0.398	0.451	0.497	0.417
CP5	ANG-L	0.060	0.085	0.055	0.005	0.011	0.015
CP5	STG-L	0.484	0.385	0.470	0.271	0.346	0.398
CP5	MTG-L	0.104	0.038	0.051	0.234	0.129	0.154
CP3	PreCG-L	0.022	0.008	0.010	0.075	0.010	0.011
CP3	PoCG-L	0.332	0.305	0.153	0.228	0.237	0.186
CP3	SPG-L	0.025	0.026	0.031	0.016	0.022	0.025
CP3	IPL-L	0.326	0.456	0.512	0.195	0.371	0.453
CP3	SMG-L	0.267	0.198	0.277	0.438	0.344	0.301
CP3	ANG-L	0.020	0.005	0.013	0.028	0.013	0.021
CP1	PreCG-L	0.159	0.098	0.111	0.239	0.180	0.173
CP1	SFGdor-L	0.013	0.003	0.005	0.022	0.008	0.008
CP1	PoCG-L	0.426	0.533	0.517	0.295	0.440	0.463
CP1	SPG-L	0.325	0.327	0.323	0.215	0.299	0.274
CP1	IPL-L	0.042	0.028	0.032	0.183	0.058	0.068
CP2	PreCG-R	0.143	0.058	0.075	0.133	0.066	0.128
CP2	SFGdor-R	0.020	0.003	0.005	0.030	0.006	0.012
CP2	PoCG-R	0.382	0.489	0.505	0.330	0.421	0.471
CP2	SPG-R	0.218	0.322	0.310	0.242	0.342	0.251
CP2	IPL-R	0.026	0.023	0.030	0.084	0.048	0.053
CP2	PCUN-R	0.141	0.078	0.045	0.042	0.036	0.021

SD distance = 30 mm		Circumferential SD pair			Vertical SD pair		
10/10 Points	AAL regions	0 year	1 year	2 year	0 year	1 year	2 year
CP2	PCL-R	0.038	0.021	0.025	0.098	0.074	0.057
CP4	PreCG-R	0.037	0.004	0.015	0.036	0.005	0.015
CP4	PoCG-R	0.326	0.239	0.272	0.281	0.285	0.341
CP4	SPG-R	0.049	0.030	0.016	0.024	0.025	0.013
CP4	IPL-R	0.466	0.676	0.661	0.450	0.638	0.585
CP4	SMG-R	0.055	0.014	0.021	0.148	0.019	0.033
CP4	ANG-R	0.058	0.037	0.014	0.051	0.026	0.012
CP6	PoCG-R	0.059	0.018	0.042	0.031	0.006	0.020
CP6	IPL-R	0.046	0.088	0.086	0.127	0.244	0.126
CP6	SMG-R	0.349	0.514	0.649	0.332	0.398	0.555
CP6	ANG-R	0.137	0.104	0.049	0.017	0.033	0.020
CP6	STG-R	0.216	0.219	0.143	0.140	0.212	0.214
CP6	MTG-R	0.185	0.055	0.030	0.340	0.106	0.064
P5	MOG-L	0.095	0.031	0.038	0.019	0.021	0.032
P5	IPL-L	0.015	0.048	0.038	0.151	0.210	0.143
P5	SMG-L	0.100	0.119	0.067	0.081	0.043	0.028
P5	ANG-L	0.257	0.559	0.534	0.272	0.405	0.470
P5	STG-L	0.244	0.126	0.138	0.076	0.052	0.029
P5	MTG-L	0.287	0.115	0.183	0.396	0.268	0.299
P3	PoCG-L	0.011	0.005	0.003	0.013	0.004	0.004
P3	SPG-L	0.104	0.106	0.084	0.109	0.135	0.090
P3	IPL-L	0.419	0.518	0.422	0.253	0.274	0.327
P3	SMG-L	0.084	0.016	0.008	0.015	0.005	0.003
P3	ANG-L	0.363	0.350	0.477	0.532	0.569	0.569
P1	PoCG-L	0.053	0.015	0.012	0.053	0.023	0.015
P1	SPG-L	0.631	0.820	0.787	0.631	0.800	0.801
P1	IPL-L	0.144	0.069	0.086	0.042	0.033	0.035
P1	ANG-L	0.086	0.060	0.077	0.209	0.125	0.120
P1	PCUN-R	0.020	0.006	0.009	0.009	0.003	0.006
P1	PCUN-L	0.045	0.026	0.027	0.018	0.010	0.017
P2	SOG-R	0.011	0.004	0.001	0.053	0.012	0.004
P2	PoCG-R	0.047	0.006	0.014	0.055	0.006	0.012

SD distance = 30 mm		Circumferential SD pair			Vertical SD pair		
10/10 Points	AAL regions	0 year	1 year	2 year	0 year	1 year	2 year
P2	SPG-R	0.482	0.610	0.639	0.466	0.673	0.701
P2	SPG-L	0.021	0.005	0.002	0.013	0.002	0.002
P2	IPL-R	0.077	0.025	0.038	0.071	0.036	0.058
P2	ANG-R	0.046	0.020	0.019	0.032	0.014	0.027
P2	PCUN-R	0.256	0.307	0.271	0.254	0.242	0.186
P2	PCUN-L	0.036	0.021	0.012	0.021	0.010	0.008
P4	SOG-R	0.026	0.005	0.002	0.009	0.003	0.002
P4	MOG-R	0.035	0.008	0.009	0.193	0.036	0.024
P4	PoCG-R	0.014	0.004	0.007	0.013	0.003	0.008
P4	SPG-R	0.159	0.292	0.169	0.171	0.269	0.202
P4	IPL-R	0.391	0.317	0.404	0.149	0.169	0.285
P4	ANG-R	0.353	0.366	0.403	0.449	0.514	0.475
P4	PCUN-R	0.011	0.007	0.004	0.007	0.003	0.003
P6	MOG-R	0.268	0.148	0.068	0.109	0.084	0.040
P6	IPL-R	0.044	0.065	0.088	0.147	0.099	0.139
P6	SMG-R	0.146	0.099	0.127	0.045	0.020	0.049
P6	ANG-R	0.340	0.615	0.665	0.507	0.702	0.652
P6	STG-R	0.049	0.008	0.006	0.009	0.003	0.004
P6	MTG-R	0.144	0.061	0.043	0.168	0.089	0.114
PO3	CUN-L	0.032	0.026	0.025	0.013	0.009	0.010
PO3	SOG-L	0.103	0.224	0.197	0.111	0.123	0.130
PO3	MOG-L	0.223	0.163	0.211	0.410	0.312	0.363
PO3	SPG-L	0.085	0.098	0.084	0.165	0.155	0.119
PO3	ANG-L	0.526	0.482	0.474	0.275	0.392	0.370
PO4	CUN-R	0.035	0.031	0.015	0.033	0.018	0.011
PO4	SOG-R	0.194	0.365	0.325	0.377	0.351	0.257
PO4	MOG-R	0.366	0.239	0.247	0.236	0.250	0.315
PO4	SPG-R	0.050	0.081	0.083	0.148	0.195	0.133
PO4	IPL-R	0.121	0.205	0.238	0.118	0.145	0.237
PO4	ANG-R	0.189	0.050	0.071	0.031	0.018	0.030
PO4	PCUN-R	0.026	0.019	0.016	0.038	0.017	0.015

Table S8 Summary of fiducial points whose MLCBR was completely consistent at some SD distances for the circumferential and vertical SD pairs across 0-yo, 1-yo, and 2-yo infants. Note: MLCBR for 2-yo was obtained by adult's optical properties of GM and WM. Adult's absorption coefficients are 0.019 mm^{-1} and 0.011 mm^{-1} for GM and WM, respectively. Adult's reduced scattering coefficients are 0.86 mm^{-1} and 4.16 mm^{-1} for GM and WM, respectively.

	Circumferential channel		Vertical channel	
Fiducial point	AAL region	SD distance (mm)	AAL region	SD distance (mm)
Cz	SMA-R	15		
AFz	SFGmed-R	20, 25, 30		
Fz	SFGmed-R	20		
CPz	PoCG-L	20, 25, 30		
POz	SPG-L	25, 30		
T3	MTG-L	10, 15, 20, 25	MTG-L	10, 15, 20, 25, 30
C5	STG-L	25, 30	PoCG-L	25, 30
C3	PoCG-L	10, 15, 20, 25, 30	PoCG-L	10, 15, 20, 25, 30
C1	PreCG-L	10, 15, 20, 25, 30	PreCG-L	10, 15, 20, 25, 30
C2	PreCG-R	10, 15, 20, 25, 30	PreCG-R	10, 15, 30
C4	PoCG-R	10, 15, 20, 25, 30	PoCG-R	10, 15, 20, 25, 30
C6			PoCG-R	10, 20, 25, 30
T4	MTG-R	10, 15, 20, 25, 30	MTG-R	10, 15, 20, 25, 30
FT7	TPOsup-L	10, 15, 20, 25	TPOsup-L	10, 15, 20, 25, 30
F7	ORBinf-L	10, 15, 20, 25, 30	ORBinf-L	10, 15, 20, 25, 30
AF7	ORBmid-L	10, 15, 20, 25, 30	ORBmid-L	10, 15, 20, 25, 30
Fp1	ORBmid-L	20, 25, 30		
Fp2			SFGdor-R	25, 30
AF8	ORBmid-R	10, 15, 20, 25, 30	ORBmid-R	10, 15
F8	ORBinf-R	10, 15, 20, 25, 30	ORBinf-R	10, 15, 20
FT8	MTG-R	10, 15, 20, 25, 30	MTG-R	10, 20, 25, 30
TP7	MTG-L	10, 15, 20, 25, 30	MTG-L	10, 15, 20, 25, 30
T5	MTG-L	10, 15, 20, 25, 30	MTG-L	10, 15, 20, 25, 30
O1	MOG-L	10, 15, 20, 25, 30	MOG-L	10, 15, 20, 25, 30
O2	SOG-R	10, 15, 20	SOG-R	10, 15, 20, 25, 30
PO8	MOG-R	10, 15, 20, 25, 30	MOG-R	10, 15, 20, 25, 30
T6	MTG-R	10, 15, 20, 25, 30	MTG-R	10, 15, 20, 25, 30
TP8	MTG-R	10, 15, 20, 25, 30	MTG-R	10, 15, 20, 25, 30
FC5	IFGtriang-L	20, 25, 30	IFGoperc-L	10, 20, 30

FC3	MFG-L	10, 15, 20, 25, 30	MFG-L	10, 15, 20, 25, 30
FC1	MFG-L	10, 15, 20, 25, 30	MFG-L	15, 20, 25, 30
FC2	MFG-R	10, 15, 20, 25, 30	MFG-R	10, 15, 20, 25, 30
FC4	MFG-R	10, 15, 20, 25, 30	MFG-R	10, 15, 20, 25, 30
FC6	IFGtriang-R	30	IFGoperc-R	10
F5	IFGtriang-L	10, 15, 20, 25, 30	IFGtriang-L	10, 15, 20, 25, 30
F3	MFG-L	10, 15, 20, 25, 30	MFG-L	10, 15, 20, 25, 30
F1			SFGdor-L	10, 15, 20, 25, 30
F2	SFGdor-R	10, 15	SFGdor-R	10, 15, 20, 25, 30
F4	MFG-R	10, 15, 20, 25, 30	MFG-R	10, 15, 20, 25, 30
F6	IFGtriang-R	10, 15, 20, 25, 30	IFGtriang-R	10, 15, 20, 25
AF3	MFG-L	15, 20, 25, 30	MFG-L	15, 20, 25, 30
AF4	MFG-R	30		
CP5			SMG_L	30
CP3	IPL-L	20		
CP1	PoCG-L	10, 15, 20, 25, 30	PoCG-L	10, 15, 20, 25, 30
CP2	PoCG-R	10, 15, 20, 25, 30	PoCG-R	10, 15, 20, 25, 30
CP4	IPL-R	10, 15, 20, 25, 30	IPL-R	10, 15, 20, 25, 30
CP6	SMG-R	10, 15, 20, 25, 30	SMG-R	10, 15, 20, 25
P5	ANG-L	10, 15, 20, 25	ANG-L	10, 15, 20
P3	ANG-L	10, 15	ANG-L	10, 15, 20, 25, 30
P1	SPG-L	10, 15, 20, 25, 30	SPG-L	10, 15, 20, 25, 30
P2	SPG-R	10, 15, 20, 25, 30	SPG-R	10, 15, 20, 25, 30
P4	ANG-R	10, 15, 20, 25	ANG-R	10, 15, 20, 25, 30
P6	ANG-R	10, 15, 20, 25, 30	ANG-R	10, 15, 20, 25, 30
PO3	ANG-L	10, 15, 20, 25, 30		
